# Disparities in model-based cost-effectiveness analyses of tuberculosis diagnosis: A systematic review

**DOI:** 10.1371/journal.pone.0193293

**Published:** 2018-05-09

**Authors:** T. I. Armina Padmasawitri, Gerardus W. Frederix, Bachti Alisjahbana, Olaf Klungel, Anke M. Hövels

**Affiliations:** 1 Division of Pharmacoepidemiology and Clinical Pharmacology, Utrecht Institute of Pharmaceutical Sciences, Utrecht University, Utrecht, The Netherlands; 2 Pharmacology and Clinical Pharmacy Research Group, School of Pharmacy, Institut Teknologi Bandung, Bandung, Indonesia; 3 Julius Centre for Health Sciences and Primary Care, University Medical Centre, Utrecht, The Netherlands; 4 TB-HIV Research Centre, Medical Faculty, Padjadjaran University, Hasan Sadikin Hospital, Bandung, Indonesia; University Medical Center Groningen, NETHERLANDS

## Abstract

**Background:**

Structural approach disparities were minimally addressed in past systematic reviews of model-based cost-effectiveness analyses addressing Tuberculosis management strategies. This review aimed to identify the structural approach disparities in model-based cost-effectiveness analysis studies addressing Tuberculosis diagnosis and describe potential hazards caused by those disparities.

**Methods:**

A systematic search to identify studies published before October 2015 was performed in five electronic databases. After removal of duplication, studies’ titles and abstracts were screened based on predetermined criteria. The full texts of potentially relevant studies were subsequently screened and excluded when they did not address active pulmonary Tuberculosis diagnosis. Quality of the studies was assessed using the “Philips’ checklist.” Various data regarding general information, cost-effectiveness results, and disease modeling were extracted using standardized data extraction forms. Data pertaining to models’ structural approaches were compared and analyzed qualitatively for their applicability in various study settings, as well as their potential influence on main outcomes and cost-effectiveness conclusion.

**Results:**

A total of 27 studies were included in the review. Most studies utilized a static model, which could underestimate the cost-effectiveness of the diagnostic tools strategies, due to the omission of indirect diagnosis effects, i.e. transmission reduction. A few structural assumption disparities were found in the dynamic models. Extensive disparities were found in the static models, consisting of varying structural assumptions regarding treatment outcomes, clinical diagnosis and empirical treatment, inpatient discharge decision, and re-diagnosis of false negative patients.

**Conclusion:**

In cost-effectiveness analysis studies addressing active pulmonary Tuberculosis diagnosis, models showed numerous disparities in their structural approaches. Several structural approaches could be inapplicable in certain settings. Furthermore, they could contribute to under- or overestimation of the cost-effectiveness of the diagnosis tools or strategies. They could thus lead to ambiguities and difficulties when interpreting a study result. A set of recommendations is proposed to manage issues related to these structural disparities.

## Introduction

The current Tuberculosis (TB) epidemic is mainly driven by untreated or improperly treated TB cases. Largely, those cases are caused by challenges in diagnosis, [1] and to reduce them, a rapid and accurate diagnosis process is required. This need is answered by the development of various novel diagnostic tools, such as the new generation of smear microscopy, e.g. Light Emitting Diode (LED) smear microscopy, and liquid culture. Both have higher sensitivity compared to their older generation tools, i.e. Ziehl-Niessen (ZN) smear microscopy and solid culture. [2,3] Another example is the new generation Nucleic Acid Amplification Test (NAAT), i.e. Xpert.MTB/Rif (Xpert) which has a significantly higher sensitivity compare to ZN microscopy, a rapid turnaround time for result, and ability to simultaneously detect drug resistant TB (i.e. rifampicin resistant strain). [3] Another novel diagnostic tool is the urine lipoarabinomannan (LAM) assay, which can be delivered at a point-of-care, although its high sensitivity is limited to TB cases with HIV comorbidity. [4]

These novel diagnostic tools often have higher cost than the existing tools. They may also require extensive resources, such as, in the case of Xpert, stabile electricity and highly trained laboratory personnel. Hence, their implementation is often hindered by budget constraints. [5] To reconcile policy and budget constraints, that is, to locate the diagnosis strategy which produces maximum health benefit at an affordable cost, decision making can be guided by the valuable information generated from model-based cost-effectiveness analyses.

In the last five years, the number of model-based cost-effectiveness analyses addressing TB diagnosis has increased tremendously. [6] This progress is, however, often outweighed by inconsistencies in modeling practices. These inconsistencies contribute to disparities among studies results, which consequently reduce studies’ comparability and transferability. [7] This situation creates complications in utilizing study results to guide decision making. Hence, identifying these inconsistencies is critical. [8]

Past systematic reviews identified modeling practice inconsistencies in cost-effectiveness analyses of TB management and intervention. They documented several methodological inconsistencies, [6,9–11] including variations in cost measurement method, choice of health outcome measures (e.g. QALY, DALY, or natural units), and study perspective (e.g. health system or societal). These reviews did not identify inconsistencies pertaining to the structure of the model used to depict disease progression. A more recent systematic review described various structure of models used to predict the cost-effectiveness of TB screening strategies and addressed their quality. [12] Although it highlighted the influence of several parameters, such as tool’s accuracy, it did not analyze the potential influence of different structural approaches on the cost-effectiveness estimates. [12] As shown by studies in other disease areas, models’ structural approaches disparities, such as different choices of health states (e.g. inclusion or exclusion of latent disease state), may substantially influence cost-effectiveness results. [13,14] Strong evidence of this influence was shown by a recent study which measured the cost-effectiveness of various TB interventions using multi models. [15] All models in the study utilized the same methodology and input parameters; however, disparities appeared in their cost-effectiveness results. These disparities could mostly be explained by the differences in the models’ structural approaches.

To the best of our knowledge, structural approach disparities in TB diagnosis model-based cost-effectiveness analyses are minimally addressed. Hence, in this study, a systematic review of model-based cost-effectiveness analyses addressing active pulmonary TB diagnosis was conducted to identify structural approach disparities and describe potential hazards caused by those disparities.

## Methods

A systematic literature review was performed to identify model-based cost-effectiveness analyses addressing active pulmonary TB diagnosis, which were published before October 2015. The review was performed and reported following the Preferred Reporting Items for Systematic reviews and Meta-Analyses (PRISMA) guideline. [16]

### Search strategy and selection process

Electronic search was performed in several databases, namely PubMed, EMBASE, Centre for Reviews and Dissemination (CRD), Cost-effectiveness Analysis (CEA) Registry, and EconLit, using a broad search term, i.e.: ("Tuberculosis") AND ("cost" OR "economic") AND ("model" OR "mathematical model”). An example of a detailed search strategy and the search date for each database is detailed in S1 File.

After removal of duplication, the title and the abstract of the studies were screened by one reviewer (TIAP) based on predetermined criteria. A study was included in the review if: 1.) it evaluated the cost-effectiveness of active pulmonary TB diagnosis process, 2.) it was published in English, 3.) it was a full economic evaluation (both economic and health consequences were evaluated), 4.) it utilized a model to generate outcome (including decision-analytic, Markov and dynamic transmission model), and 5.) its cost effectiveness result was extractable and adjustable to the current value. A study was excluded following exclusion criteria detailed in Table 1. The study’s full text report was referenced when the abstract contained ambiguous information.

**Table 1:** Exclusion criteria and the number of excluded studies per criteria.

Exclusion Criteria	Number of excluded studies
*Exclusion criteria for title and abstract screening*	
Studies was performed on animal subject	55
The focus of study was not pulmonary TB (assessing non-TB mycobacterium disease, or other related diseases such as HIV)	253
The study was not an economic evaluation (e.g. clinical trial, policy analysis)	333
The study was published in languages other than English	39
The detailed result could not be accessed	43
The study was not a full economic evaluation (e.g. cost analysis, quality of life measurement) or did not use model to generate outcome	222
The study did not address cost-effectiveness of TB diagnosis tools/strategy	140
**Total excluded studies**	**1085**
*Excluded Study Objectives (following full text screening)*	
The study addressed extra-pulmonary TB diagnosis	1 [56]
The study addressed optimization of diagnosis sample collection	1 [57]
The study addressed strain typing or mainly drug-resistant identification	3 [58–60]
The study addressed screening in non-symptomatic subject	5 [61–65]
The cost-effectiveness result could not be extracted and adjusted to the current value	4 [66–69]
**Total Excluded Studies**	**14**

Following the initial screening, the full texts of the included studies were further screened. At this stage, studies were excluded when their main objective did not focus on active pulmonary TB diagnosis. This was done to remain consistent with the objective of the review. The excluded study objectives are detailed in Table 1.

A sample of 50 studies was taken from all studies found during the search process. The sample was re-screened by a different author (AMH) to validate the screening process. Articles excluded due to language restriction and inaccessible detailed results were analyzed further to avoid exclusion of relevant studies.

### Quality assessment

The framework for quality assessment of decision analytic models (Philips’ checklist) [17] was utilized to assess the quality of the included studies. Philips’ checklist is the recommended tool for quality assessment of model-based studies. Several items in the Philips’ checklist were modified to accommodate specific quality characteristics pertaining to diagnosis. [18]

The quality assessment for each study was performed by two reviewers (TIAP and AMH; or TIAP and GWF). Discrepancies between the two reviewers were resolved through a discussion. When the discrepancies could not be resolved, a third reviewer was involved. Details on our approach to utilize Philips’ checklist for quality assessment can be found in S1 File.

The quality assessment results were analyzed qualitatively. The assessment items that were associated with the structural approaches of the model were highlighted.

### Data extraction and analysis

Data extraction was performed on items related to the following topics: 1.) general information, 2.) main outcomes, and 3.) TB progression modeling. It was performed using standardized forms, developed in Microsoft Excel™ guided by previous studies. [18–20] The forms and complete extracted data can be found in S2–S4 Tables of the supporting information. The data extraction was performed by one reviewer (TIAP). However, the results were discussed with other authors, mainly authors who performed quality assessment on the studies (AMH or GWF).

The general information covered the study’s characteristics, including study’s methodological approaches, choice of modeling framework, and cost-effectiveness conclusion. It also included the settings in which the analysis was performed. Study settings were categorized as high burden TB settings if they were listed by World Health Organization (WHO) as among the 22 high TB burden and 41 high TB/HIV burden countries. [21] Settings were categorized as moderate or low burden settings based on the claim of the studies.

Extracted main outcomes were Incremental Cost Effectiveness ratio (ICER) and/or Average Cost Effectiveness Ratio (ACER). ICERs or ACERs published in United States dollars (USD) were adjusted to 2015 value using the Consumer Price Index (CPI). [22] ICERs or ACERs published in other currencies were adjusted to 2015 value using the CPI followed by conversion to USD using Purchase Power Parity (PPP). [23–27] The adjustment method followed the recommendation from WHO. [28]

TB progression modelling topic covered the inclusion/exclusion of disease and setting characteristics in the model, as well as structural assumptions. Structural assumptions encompassed all study’s assumptions which influenced the structure of its model.

A pilot study performed following the development of the data extraction forms, tested the applicability of the forms. Its result was discussed among authors and necessary changes, as well as additional fields were incorporated into the forms.

The extracted data were analyzed qualitatively. The cost-effectiveness conclusions and main cost-effectiveness outcomes of similar studies (i.e. those performed in the same setting and addressed similar diagnostic tools) were assessed to find variability. The methodological approaches of the studies were assessed to confirm inconsistencies found in previous reviews. In the main analysis, models’ structural approaches were assessed to find variability. Their applicability in various settings and potential influence on the cost-effectiveness result were also analyzed. The structural approaches assessed were the choice of modelling framework, the inclusion/exclusion of disease and setting characteristics in the model, and the structural assumptions.

## Results

**Fig 1:**
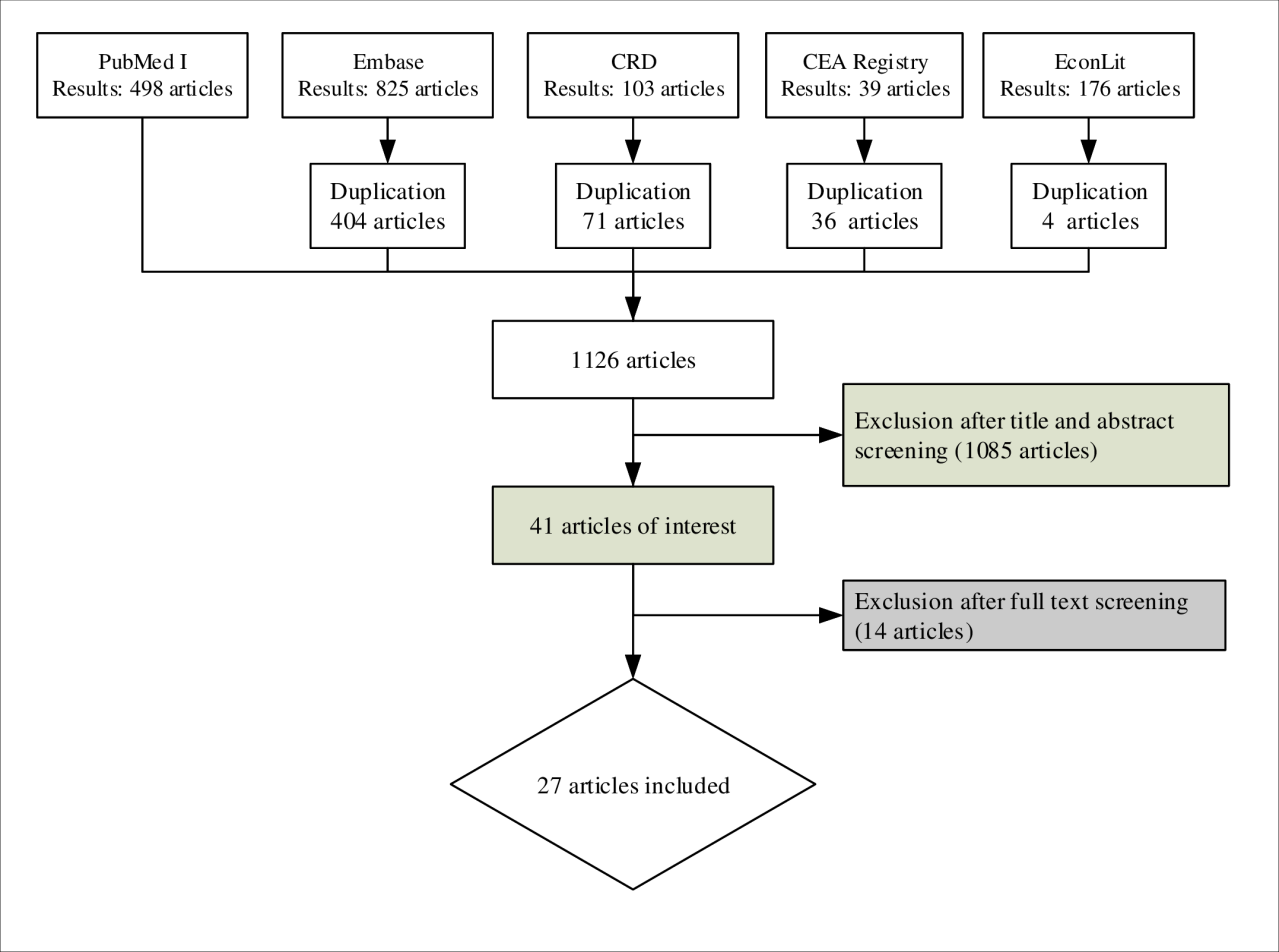
The study search and selection process.

After removing duplications, the search in five electronic databases identified 1126 articles. Of these, only 27 fulfilled the inclusion criteria and were included in the review. [29–55] This article selection process is depicted in Fig 1.

In total, 1099 studies were excluded. The number of studies excluded for each exclusion criterion is given in Table 1.

Most studies (78%) were performed in high burden TB settings, i.e. Brazil, [34,35,38,47,48] India, [36,40,42,50,52,53] several countries in Southern Africa (Botswana, Lesotho, Namibia, South Africa and Swaziland), [31,35,43,51–53] and East Africa (Kenya, Malawi, Uganda, Tanzania and Zambia). [30,35,41,44,46,49,51–53,55] One study performed analysis in a high burden region, i.e. Sub-Saharan Africa. [29] The remaining studies were performed in low to moderate burden settings, i.e. Finland, [45] UK, [39] US, [32,33,37] and Hong Kong P.R. China. [54] More than half of the studies (63%) were performed within 2011–2015. [29,32,36,38–43,48–55] The earliest study dated from 1998. [46]

The included studies evaluated the cost-effectiveness of various active pulmonary TB diagnosis tools and strategies. Two studies evaluated the negative impact of serology based testing for active disease diagnosis in high burden settings. [36,42]

**Fig 2:**
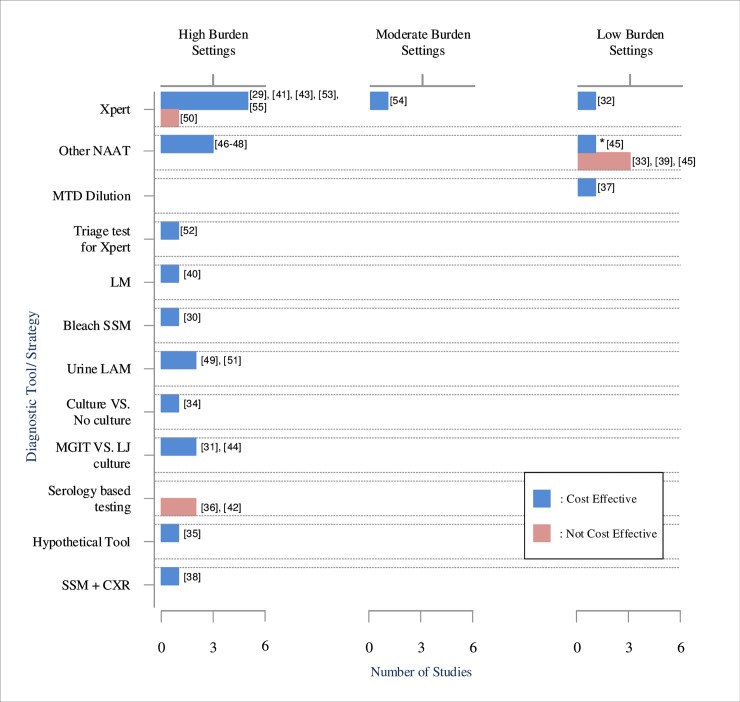
Cost-effectiveness conclusion of novel diagnosis strategies. NAAT = Nucleic Acid Amplification Technique, MTD = Mycobacterium Tuberculosis Direct Test, LM = Light-Emitting Diode Microscopy, SSM = Sputum Smear Microscopy, LAM = Lipoarabinomanan assay, MGIT = Mycobacteria Growth Indicator Tube, LJ = Löwenstein–Jensen, CXR = Chest X-Ray. * Secondary analysis by Rajahlati et al. showed different cost-effectiveness conclusion from the primary analysis (NAAT for all TB presumptive cases). NAAT was cost effective when applied selectively on smear positive cases due to its high sensitivity and the high rate of true positive in this group. [45].

Almost all studies concluded that novel diagnosis tools or strategies were cost effective compared to the best available diagnosis, as depicted in Fig 2. Exceptions were found in three studies assessing NAAT (e.g. Xpert and Mycobacterium Tuberculosis Direct Test or MTD) in low-burden settings, [33,39,45] two studies evaluating serology based testing in high burden setting, [36,42] and one study investigating TB diagnosis in private and public health sector. [50]

**Fig 3:**
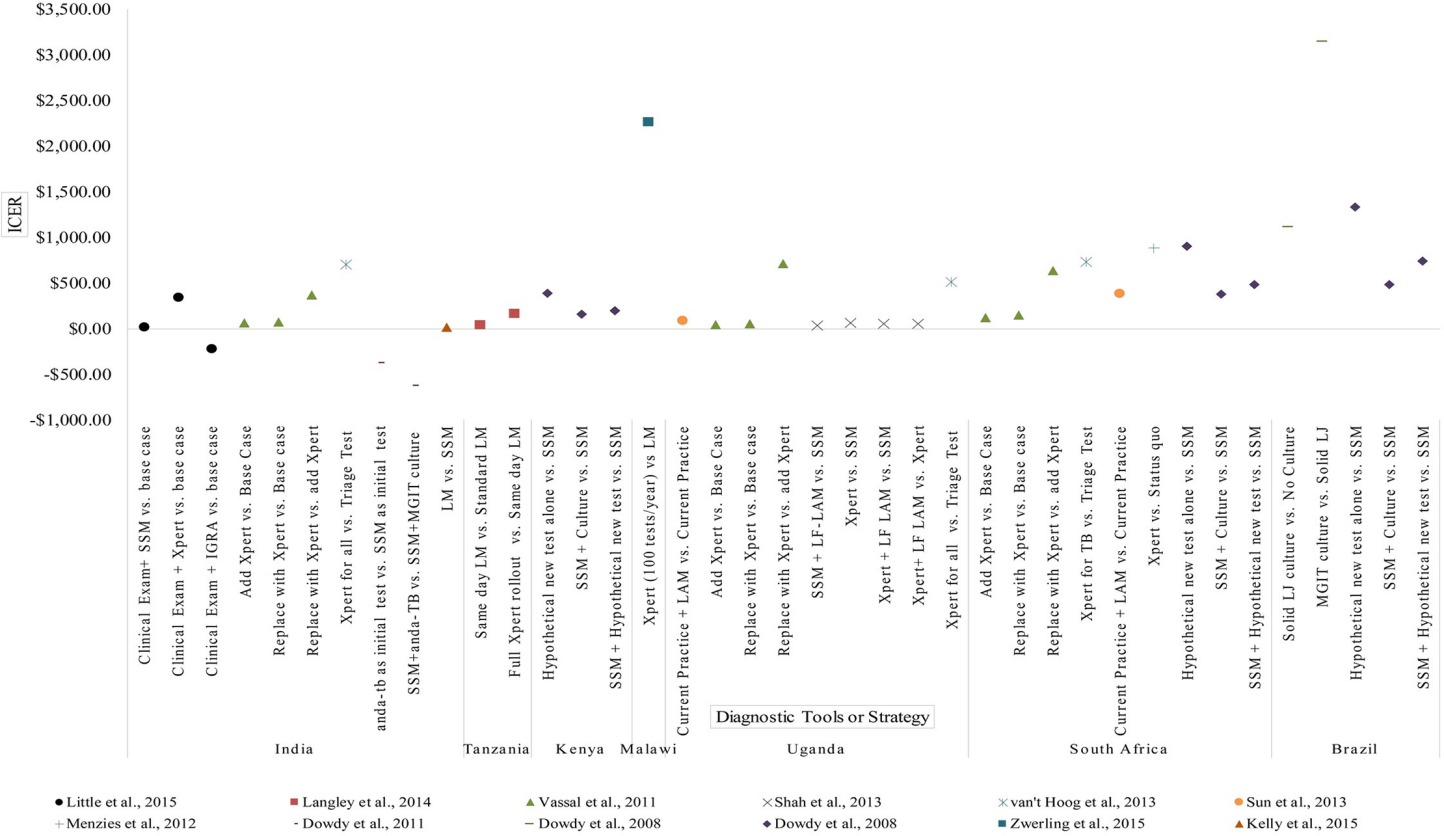
ICER of studies which used DALY (A). DALY = Disability Adjusted Life Years, SSM = Sputum Smear Microscopy, Xpert = Xpert MTB/RIF, IGRA = Interferon-Gamma Release Assay (e.g. anda-TB), MGIT = Mycobacteria Growth Indicator Tube, LM = Light-Emitting Diode Microscopy, LF-LAM = Lateral Flow Urine Lipoarabinomannan Assay, LJ = Löwenstein–Jensen.

**Fig 4:**
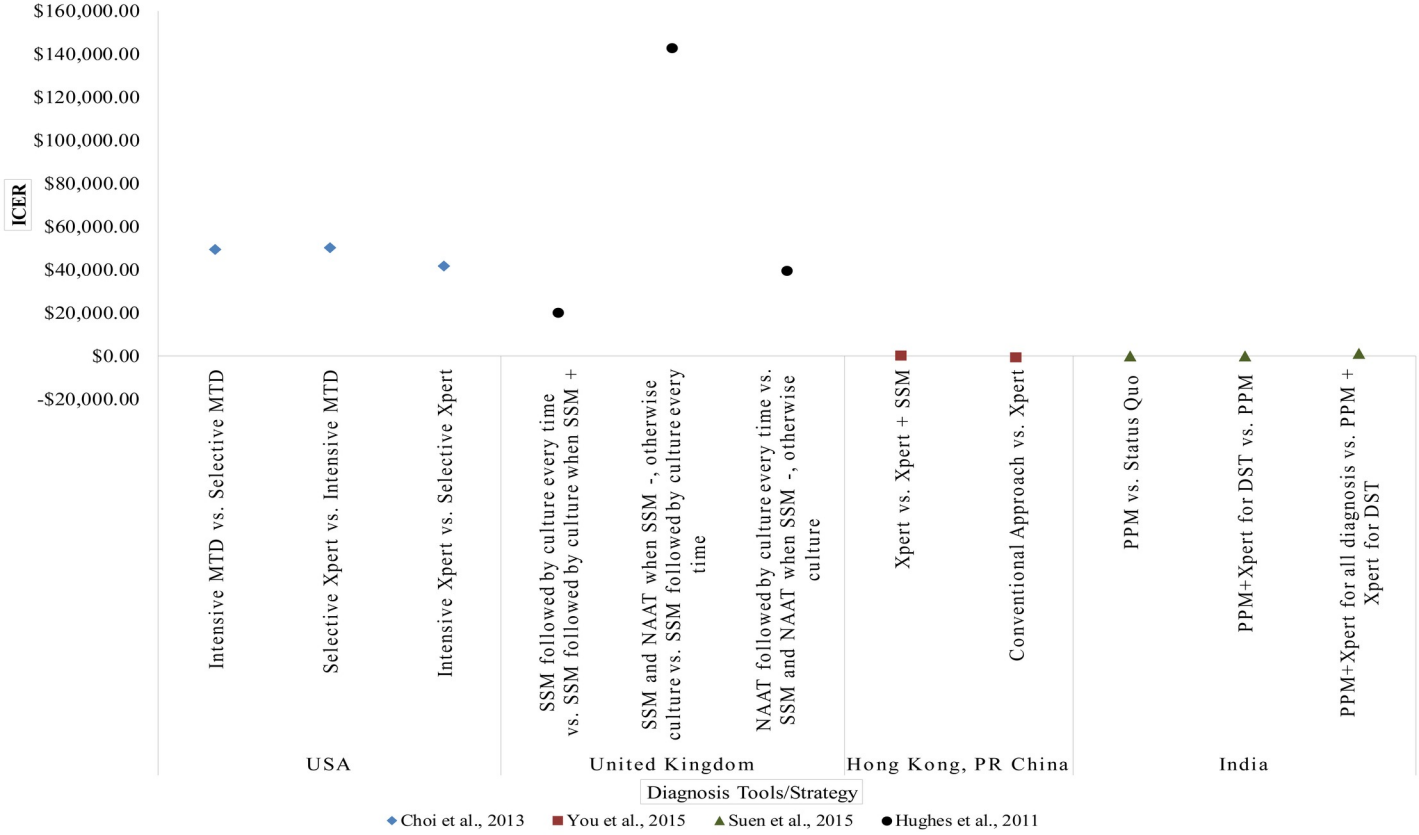
ICER of studies which used QALY (B). QALY = Quality Adjusted Life Years, MTD = Mycobacterium tuberculosis Direct Test, Xpert = Xpert MTB/RIF, SSM = Sputum Smear Microscopy, NAAT = Nucleic Acid Amplification Technique, PPM = Public Private Mix Program.

Despite the high degree of agreement in cost-effectiveness conclusion, ICER and/or ACER value for similar tools or strategies varied between studies and settings. This can be seen in Figs 3 and 4, which depicts the ICER of various diagnosis strategies from studies utilizing generic health outcomes measurement, i.e. Disability Adjusted Life Years (DALY) and Quality Adjusted Life Years (QALY). The adjusted cost-effectiveness outcome for all included studies can be found in S3 Table.

Two studies which evaluated the cots-effectiveness of implementing Xpert as an initial test for all TB cases in South Africa exemplified the variations of cost-effectiveness outcome. [43,53] Both studies, published one year apart, utilized similar comparator and Xpert test price. However, after adjustment to 2015 value, one study predicted an ICER value of USD 749.82, [43] while the other predicted an ICER of USD 149.55. [53]

As detailed in Table 2, the included studies utilized various methodological approaches. Several studies were found to neglect certain good quality attributes, especially those associated with the models’ structural approach. Around half of the studies failed to report structural assumptions and their justification. [29–34,36–38,40,42,44,46–49,51] Moreover, none of the studies performed adequate structural uncertainty analysis. All studies also failed to report adequately the synthesis process of the diagnostic tools’ accuracy. A complete quality assessment of the studies can be found in S1 Table.

**Table 2:** Methodological variations and quality attributes found in the included studies.

Items	Adherence and Non-Adherence to Good Quality Attributes
1. Study perspective	Only 7.4% of the studies adopted a societal perspective. [48,50] 7.4% of the studies did not mention the study perspective, but included patients’ cost. [46,47]
	One study included patients' travel cost, although the study perspective was health service provider. [30]
2. Cost input	Inconsistencies between study perspective and cost input were observed. Of the 16 studies [29,32–36,38–43,49,51,52,55] taking a health system perspective 8 omitted the overhead cost. [29,33–35,38,39,42,51]
	Other potentially relevant costs, e.g. hospitalization cost and HIV comorbidity treatment cost, were omitted (mainly due to unavailability of data) without proper justification. [51,54]
3. Health outcome measurement	DALY (44%), [34–36,40–43,49,51–53,55] QALY (15%), [32,39,50,54] others (case detected or correct diagnosis, early exclusion of TB, death averted), [29–31,33,37,38,44–48]
	Most studies did not detail the DALY calculation method; however, several disclosed the omission of age weighting. [41,42,55] Methods to derive utility value were mostly undisclosed. In some cases, utility was derived from outdated sources (published in 1988 and 1998) and did not utilize a currently acceptable method for Health-Related Quality of Life (HRQOL) measurement. [32,54,70–72]
4. Data synthesis method	Information regarding pre-model data analysis, including methods to derive diagnostic tool accuracy, was not disclosed adequately in all studies.
5. Uncertainty consideration	52% of the studies conducted univariate, multivariate sensitivity analysis, as well as PSA. [29,32,34,39,40,42,43,49–55]
	None of the studies adequately addressed methodological and structural uncertainty. Methodological uncertainty exploration was limited to investigating the impact of various discounting rates. [31,34–36,55]

**Fig 5:**
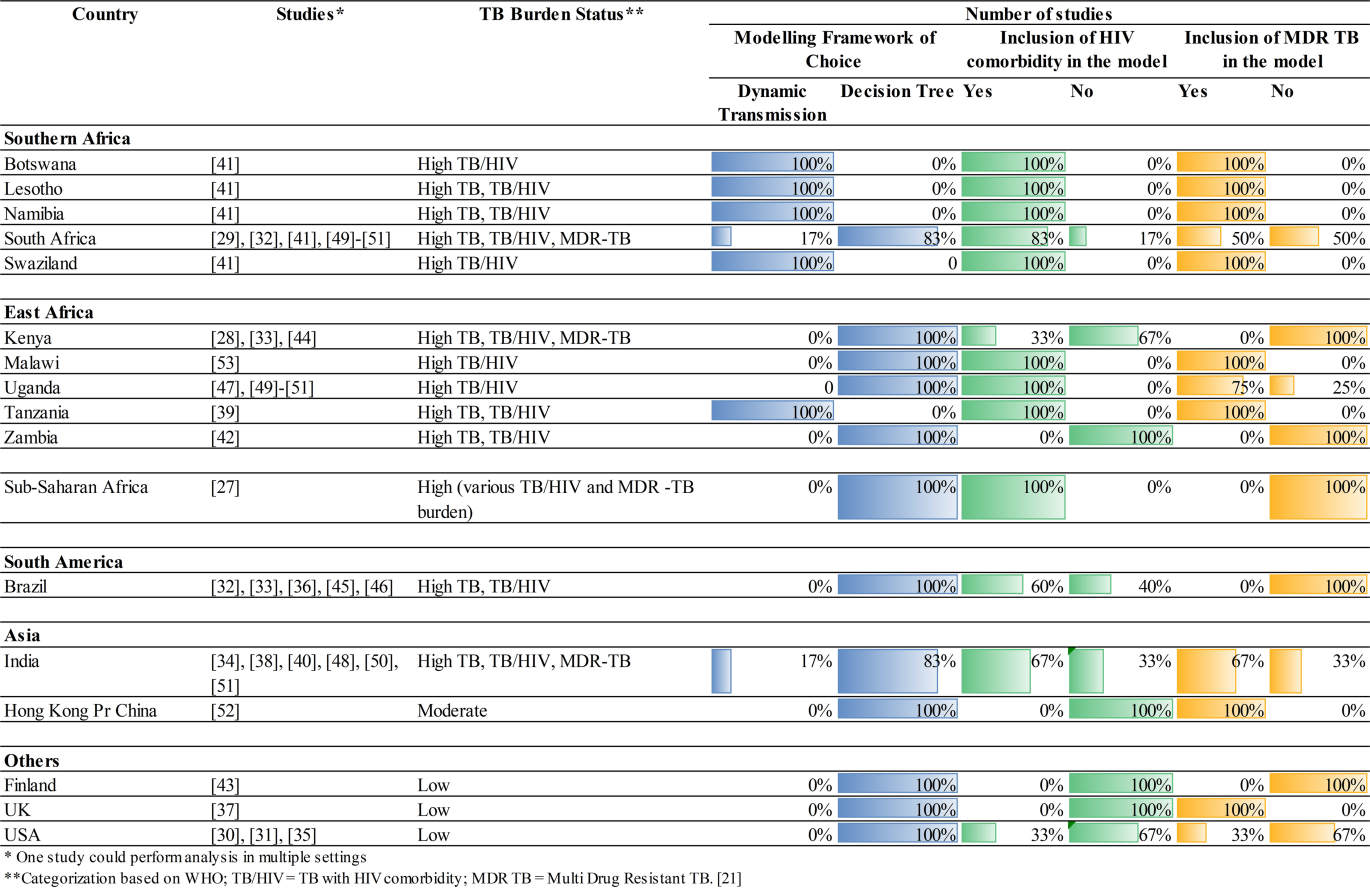
Variations of modeling framework, HIV comorbidity and MDR TB inclusion.

Disparities in the models’ structural approaches were also found among the studies, including different choices of modeling framework. The static model framework, i.e. single cohort Decision Analytic Model (DAM) consisted of a decision tree, was most commonly utilized by the studies (89%). [29–40,42,44–49,51–55] One study utilized DAM consisted of a decision tree in combination with a Markov model to simulate re-diagnosis of false-negative patients. [34] The dynamic model framework was used by three studies which conducted their analysis in high burden settings. [41,43,50] Studies’ choices of modeling framework are summarized in Fig 5.

As detailed in Fig 5, studies generally included important setting characteristics, i.e. Human Immunodeficiency Virus (HIV) comorbidity and Multi Drug Resistance (MDR) TB. The inclusion of these characteristics reflected the disease burden in the study settings. However, these characteristics were omitted by several studies conducted in important settings with three overlapping burdens (high prevalence of TB, TB-HIV comorbidity, and MDR TB), i.e. India and South Africa. [31,35,36,40,50,51] Studies that modeled HIV comorbidity differed in modeling the influence of HIV on TB progression, e.g. influence of HIV towards smear status.

Two studies included setting characteristic in addition to HIV comorbidity and MDR TB, i.e. different quality of TB care in public and private sector. [43,50] One study, in which the setting was dominated by sub-standard private sector care, showed that Xpert alone without a program to transfer patients from private to public sector was not cost effective. [50] Another study in the same setting, which did not consider this characteristic, concluded that Xpert alone was cost effective. [53]

Studies using a dynamic model framework showed few structural assumptions disparities. One of the studies omitted the active transmission process of MDR TB strain. [41].

In contrast, the 24 static models showed extensive structural assumption disparities. These included different assumptions regarding treatment outcomes, clinical diagnosis and empirical treatment, inpatient discharge decision, and re-diagnosis of false negative patients. The disparities are detailed in Table 3.

**Table 3:** Variations of structural assumptions in static models.

No.	Structural Component	Variations of Assumption
1	Treatment outcome	Diagnosed patients were all successfully treated (8/24 studies). [32,34,35,39,40,42,47,54]
		Treatment failure was also considered (5/24 studies). [29,36,51–53]
		Treatment failures as well as loss to follow up were considered (2/24studies). [49,55]
		Treatment outcome was not modeled (9/24 studies). [30,31,33,37,38,44–46,48]
2	Clinical diagnosis and Empirical Treatment	A fixed proportion of patients received clinical diagnosis and empirical treatment, which usually had a low accuracy. This proportion was not changed by the availability of diagnostic tools/strategy with better accuracy (3/24 studies). [34,42,51]
		Several studies tested several assumptions regarding the influence of diagnostic tool/strategy with better accuracy on clinical diagnosis and empirical treatment in sensitivity analyses (2/24 studies). [52,53]
		Decision to perform clinical diagnosis and empirical treatment could be corrected by the result of diagnostic tool with higher accuracy (1/24 studies). [32]
		Diagnostic tools/strategy with better accuracy, reduced or eliminated the need to perform clinical diagnosis and empirical treatment (4/24 studies). [29,46,49,54]
		Clinical diagnosis and empirical treatment was not considered or the impact of diagnostic tool/strategy with better accuracy on empirical treatment was not detailed (14/24 studies). [30,31,33,35–40,44,45,47,48,55]
3	Discharge decision from inpatient care (Applied in studies conducted in low burden settings)	Negative result from a rapid and highly accurate novel diagnostic tool was sufficient to release patients from respiratory isolation. However, it was not clear whether the result was sufficient to discharge patients from inpatient care (1/4 studies). [33]
		Result from rapid and highly accurate novel diagnostic tool was sufficient to discharge patients from respiratory isolation and inpatient care. (2/4 studies). [32,45]
		TB patients were not managed as inpatient (1/4 studies). [39]
4	Re-diagnosis of false negative patients	Modeled the reintroduction of false negative patient to the health system for a second diagnosis (6/24 studies). [34,40,49,52,53,55] One study calculated the cost for false negative. [47]
		Reintroduction of false negative patient was not possible (17/24 studies). [29–33,35–39,42,44–46,48,51,54]

## Discussion

The wide range in the cost-effectiveness ratio (ICER and/or ACER) observed in the included studies could be the result of different study settings and populations. However, it could also have been influenced by modeling practice inconsistencies. As reported in one study, modeling practice inconsistencies, such as utilization of different methodological and structural approaches, were strongly related to the wide range of results. [7] Modeling practice inconsistencies identified by previous reviews, i.e. variations in methodological approaches, [6,9–11] were still prevalent among the included studies. The included studies were also afflicted by quality issues pertaining to structural approaches identified by a previous review, such as failure to address structural uncertainty. [12] In addition, the studies also showed extensive disparities in the structural approaches.

As in previous reviews, [6,10] a static model framework, which omitted TB transmission process, was the preferred options among the included studies. This is understandable, since static models development is relatively straightforward and can be easily understood by non-modelers, including decision makers. In contrast, dynamic models require complicated mathematical computation. Furthermore, their development is challenged by the difficulties in modeling the transmission process itself [73] especially when data and knowledge regarding the transmission process is limited.

However, the use of static models to address infectious disease intervention strategies may potentially underestimate the indirect effect of the strategies in preventing secondary cases. [73,74] This issue was recognized by almost all studies which utilized a static model framework; however, they argued that the underestimation would result in a more conservative estimate. The argument should be considered carefully, since the underestimation may not only affect health benefit but also the demand of intervention and its related cost. [74]

The use of a dynamic model framework is recommended for investigating an infectious disease management strategy that influences the disease transmission process. [75] Diagnostic strategies may influence the TB transmission process through mitigating the main infection source, e.g. untreated active disease consisting of undiagnosed (e.g. diagnostic loss to follow up) and false negative cases. They may also influence transmission process due to their operational aspect, such as a rapid process to obtain diagnosis result. The rapid process causes shorter delay in starting treatment, which consequently causes shorter period of infectiousness. [76] The influences of diagnosis strategies on the TB transmission process were consistently observed in all included studies which utilized dynamic models. [41,43,50]

The ongoing TB transmission process in high burden TB settings is an important factor that might hinder countries progressing toward TB control goals. [77] The impact of a diagnostic strategy on the ongoing transmission process is better explained by a dynamic model framework; hence, the use of such framework may be more beneficial for high burden settings.

A dynamic model framework may also be useful in facilitating long term analysis. Studies utilizing a dynamic model framework in a setting with a high prevalence of TB and HIV comorbidity could identify budget increase in the long term (10 to 20 years), not only for TB management, but also for Anti-Retroviral Treatment (ART), due to the higher survival rate of patients. [41,43] This finding was not observed in the static models. [43]

HIV comorbidity and MDR TB were included in the model governed by underlying assumptions driven by currently limited knowledge and data. [77] Thus, we found various assumptions regarding HIV influence on TB as well as the MDR TB transmission process. Furthermore, these characteristics were excluded in several relevant settings. This exclusion potentially influences study result, e.g. by overestimating the effectiveness of diagnosis tools with lower accuracy in HIV positive individuals. An evidence of this influence was shown by a recent multi-model study. [78] In the study, the inclusion and exclusion of HIV interaction with TB in the models led to differences in TB outcome projections.

Other important setting characteristics include various key drivers of TB epidemics and health system characteristics, such as domination of sub-standard private sector TB care. [79] As shown by one study, including a sub-standard private sector in the model could influence cost-effectiveness results. [50] Unfortunately, currently, data and knowledge for this characteristic are limited. One example of this limitation is the uncertainty regarding the impact of shifting patients from private sector to the high quality public sector care. [15] Another example is the uncertainty surrounding the number of TB cases managed in private sector due to underreporting. [80] More research into this characteristic will be valuable to inform model construction.

Other disparities found concerned the structural assumptions, which were more prevalent among the static models. Assuming a successful treatment outcome for all correct diagnosis was applied by several studies. This assumption would not apply in settings with significant numbers of unsuccessful treatment outcome such as treatment loss-to-follow-up. Assuming only successful treatment outcome in such settings may lead to an overestimation of the cost-effectiveness. This was proven by an empirical study in South Africa which showed that extensive loss to follow up mitigated the benefit of an accurate diagnosis by a novel tool. [81]

Another structural assumption disparity concerned the practice of clinical diagnosis, consisting of additional diagnosis, such as Chest X-ray, followed by an empirical TB treatment. It is usually performed in highly suspected TB cases which obtain negative results for the main diagnosis tests such as smear microscopy.

As argued by one study, the effectiveness of novel diagnosis tools could have been overestimated by modeling studies when clinical diagnosis practice was underestimated. [82] Novel diagnosis tools’ effectiveness is influenced by the number of additional diagnosed TB cases, which would have been undiagnosed by the existing diagnosis practice. When a rigorous clinical diagnosis practice is applied, as found in several high burden settings, [82,83] most TB cases will be diagnosed and treated under the existing diagnosis practice. Consequently, this will minimize the number of additional cases diagnosed by the novel diagnosis tools and lower their effectiveness. [82,83]

Another study argued that assuming clinical diagnosis practice to be unchanged by the availability of novel diagnostic tools with better accuracy could underestimate the effectiveness of the novel tools. [41] This was due to the expectation that novel tools would increase physicians’ certainty of a negative result; thus, reducing the need to perform additional clinical diagnosis which could introduce false positive cases due to its low specificity. Unfortunately, the influences of novel diagnosis tools on clinical diagnosis practice have not been fully understood. [83] However, clinical diagnosis practice had a significant influence on novel tool’s cost-effectiveness, as shown by a recent empirical study in South Africa. [81] Thus, it is important to consider this practice in the model.

In settings where TB is managed in an inpatients-setting, the assumption regarding decision to discharge from hospital or isolation can also be influential. The assumption, for instance, that a patient could be discharged solely based on Nucleic Acid Amplification Technique (NAAT) negative result, could lead to a positive cost-effectiveness for NAAT use in low burden TB settings. [32, 45] The speed with which NAAT result was obtained leads logically to a faster decision to discharge, thus minimizing hospital stay and its related cost.

Disparities were also found in assumptions around the false negative re-diagnosis process. Since most settings do not actively screen for TB, the re-diagnosis process may rely heavily on individuals’ discretion to seek another diagnosis. In high burden settings, this may be challenged by several circumstances such as long distances between home and the health center. Hence, the re-diagnosis process could have been overestimated, and could eventually cause diagnosis tools’ or strategies’ effectiveness overestimation.

Despite its important findings, this review could have overlooked some relevant studies, such as those published in languages other than English and those with inaccessible detailed result. However, additional analysis performed to studies excluded based on these two criteria did not result in additional inclusion of studies. The complete description of the additional analysis can be found in S1 File.

A meta-analysis could be performed on the cost and health benefit estimates of the reviewed studies to produce a single estimate of ICER. However, the single estimate might be futile since it is considered non-transferable to any setting. [84–86] An ICER estimate addresses an isolated problem in certain health-system, thus its component cannot be freely transferred to different settings. This transferability issue has resulted in the absence of a recommended meta-analysis method for cost-effectiveness analyses results. [86] Furthermore, a meta-analysis was not instrumental in achieving the aim of the study.

Bias could be introduced during the screening process, which was done by only one author. However, this risk of bias was reduced by performing validation. It showed that the level of agreement among two authors for abstract and title screening reached 88%, and a consensus was reached following a short deliberation. Bias could also be introduced during data extraction, since it was also conducted by one author. It was, however, reduced through extensive discussions among the co-authors; especially with those who also analyzed the studies for quality assessment.

This review included studies conducted in multiple settings and covered a variety of diagnostic tools and strategies. It also managed to confirm past systematic review findings. Thus, inclusion of more studies in this review would most likely substantiate current findings.

PRISMA guideline mandates systematic review to assess the individual study’s risk of bias. Indication of bias was shown by the extensive disparities of cost-effectiveness result among studies with similar diagnostic strategies and settings. This bias could be caused by methodological inconsistencies, including structural approach disparities. Another source of bias, which is not discussed in this review, is input parameters. Input parameters may have reliability issue. For example, several settings in Southeast Asia showed a high burden of TB, but population screening found that actual TB prevalence was significantly higher than what had been estimated through TB program case notification. [80] Another example of this reliability issue is the lack of transparency in reporting the method to synthesis diagnostic tools’ accuracy.

The structural approaches of the models were often reported inadequately. This practice could cause difficulties for decision makers when assessing the suitability of an approach to their setting, which emphasizes the need to report structural approaches and their justification transparently. In addition, model validation should be reported to ensure decision makers’ confidence in the model. This can be done following a recent guidance on model validation reporting. [87] Another important practice, often omitted, is to address uncertainties introduced by different choices of structural approaches. An example of this was shown in a recent study that used multi models and summarized the models’ cost-effectiveness results. [15] A summary of practice and reporting recommendation to manage issues related to model’s structural disparities can be found in Table 4.

**Table 4:** Recommendation of approaches to manage issues related to structural disparities.

Items	Recommendation
Modeling Framework	Dynamic transmission model should be utilized for planning purposes and in situations where the impact of TB diagnosis strategy towards transmission process is an important aspect to consider.
Structural Assumptions:	
1. Treatment Outcome	Omission of certain treatment outcome may cause overestimation of cost-effectiveness; hence it should be justified clearly; e.g. omission of treatment failure in low burden settings due to the high rate of treatment success.
2. Clinical Diagnosis Practice	Assumptions regarding clinical diagnosis practice should consider aspects which will influence its loss/benefit, i.e. accuracy of clinical diagnosis, the likelihood of TB cases to be treated under clinical diagnosis practice and novel diagnosis tools, as well as changes in clinician’s behavior regarding clinical diagnosis practice upon the introduction of novel diagnostic tools. [83]
3. Discharge decision from inpatient care or isolation	When TB is managed as inpatient, studies should report the details regarding discharge decision, importantly the diagnostic results which leads to the decision; e.g. discharge decision is based on negative result from rapid diagnosis or only possible if mycobacterium culture is negative. The common practice in clinical settings should be considered.
4. Re-diagnosis of false negative patients	Assumption regarding re-diagnosis of false negative patients should be detailed and justified; e.g. re-diagnosis of false negative patients is possible for a certain period through routine active case findings performed in the settings or due to symptoms escalation.
5. HIV comorbidity and MDR TB	Studies should state whether HIV comorbidity and MDR TB are considered in their model. Influence of HIV on TB progression should be detailed (e.g. higher rate of smear negative in HIV or lower diagnosis accuracy in HIV positive patients) and clinical grounds for such assumptions should be mentioned. Consequences of MDR TB should be detailed (e.g. higher treatment failure, higher mortality rate). Studies should also disclose whether MDR TB transmission is considered or not, and clinical grounds for such assumption should be mentioned (e.g. omission is based on lower transmission rate of MDR TB compare to drug sensitive strain, due to the fitness cost of the drug resistant strain).
6. Other setting characteristics	Other important setting characteristics which may influence TB epidemics can be considered in the model, e.g. dominance of sub-standard private practice or inpatient care for TB management. Inclusion or omission of such characteristics should be justified and reported.
Structural uncertainty	Uncertainty caused by inclusion or omission of certain structural approaches should be assessed systematically through structural uncertainty analysis. Examples of methods used to address structural uncertainty are scenario analysis and multi-models analysis. [15,88]
Model Validation	Relevant model validation process, such as face validation to assess suitability of the model to represent the underlying clinical process of TB, [87] should be performed and reported. This will help to ensure decision makers’ confidence on the model and study’s result.

Modeling practice inconsistencies, found in the past and in the current review, highlight the need for a clear standard for study conduct and reporting, especially in high burden TB settings, which mostly consists of Low-Middle Income Countries. Moreover, the standard should be enforced by important stakeholders, such as shown by The Bill and Melinda Gates Foundation, which developed a case-reference containing general methodologic specifications and reporting standards. [89] A disease-specific standard may also give additional benefit to model’s structure development.

## Conclusion

This systematic review identified extensive structural approach disparities in model-based cost-effectiveness analyses addressing TB diagnosis strategies. It shows that several structural approaches could be inapplicable in certain settings. Furthermore, certain approaches could potentially contribute to under- or overestimation of the cost-effectiveness of a diagnosis tool or strategy. Eventually, they will lead to ambiguities and difficulties when interpreting the study result. This issue can be managed by reporting the model’s structural approaches and their justification transparently. Furthermore, this lack of clarity may also be reduced by addressing and reporting model’s validity and structural uncertainty.

## Supporting information

S1 File Details of the search and screening strategy, and quality assessment approach. (PDF)

S2 File Completed PRISMA checklist. (PDF)

S1 Table Quality assessment of the included studies. (PDF)

S2 Table Data extraction for general information. (PDF)

S3 Table Data extraction for main outcomes. (PDF)

S4 Table Data extraction of tuberculosis progression modeling approaches. (PDF)
