# Supplementary material for: Disparities in model-based cost-effectiveness analyses of tuberculosis diagnosis: A systematic review
Source: PLoS One. 2018 May 9;13(5):e0193293. doi: 10.1371/journal.pone.0193293 (PMC5942841; doi:10.1371/journal.pone.0193293)
Supplement: S1 File — (PDF) [file pone.0193293.s001.pdf]

Search Strategy

As stated in the main article, we utilized a wide search terms to identify relevant published model-based cost effectiveness analyses, i.e. ("Tuberculosis") AND ("cost" OR "economic") AND ("model" OR "mathematical model"). An example of a complete search string used in PubMed is as follow:

- ("Tuberculosis" OR "Tuberculosis" [MeSH Terms]) AND
- ("Cost-Benefit Analysis" [mh] OR (cost AND effective*) OR (cost AND utility)) AND
- ((model*) OR model, economic [MeSH Terms])

The search date for each database is as the following:

- PubMed : July 8th, 2015;
- EMBASE : July 28th, 2015;
- Centre for Reviews and Dissemination (CRD) : July 29th, 2015;
- Cost-effectiveness Analysis (CEA) Registry : August 6th, 2015;
- EconLit : August 6th, 2015

Exclusion of Studies based on Language Restriction and Accessibility

Further analysis was performed to studies excluded due the use of non-English language in the article. These studies were published in Chinese (3), Japanese (15), French (5), German (1), Norwegian (1), Polish (2), Portuguese (2), Romanian (2), Russian (3), and Spanish (5). Out of these studies, based on their title and abstract, three studies were regarded as possibly relevant. [1–3] However, these studies were published before 1980. Hence, the studies were most likely to address outdated diagnostic tools or strategy, and might be performed without following currently acceptable modeling practice. They were also not conducted in important TB settings. Thus, the studies were not included in the review. Other studies were not considered relevant (studies addressed history and policy of TB management, cost-effectiveness/benefit analysis of screening, contact investigation, and treatment program, as well as performing epidemiological studies).

Articles excluded based on inaccessible detailed result were also analyzed. Most of these studies were published in a proceeding for a poster or oral presentation. Out of the 43 articles, five articles were possibly relevant. [4–8]Three out of these five articles were fully published with a different title and included in the review. [9–11] One study was fully published; however it was excluded since it did not conduct a full economic analysis. [12] Another study was not fully published, however it was excluded based on its abstract since it addressed latent TB screening in high risk individuals.[7] Other studies were not consider relevant (studies were not a full economic analysis, addressed cost-effectiveness of screening, prevention, treatment, and TB control program in general, as well as performing policy analysis and epidemiological study)

Quality Assessment Approach

We performed quality assessment on all of the included studies utilizing "The framework for quality assessment of decision analytic models" (Philips' checklist). The checklist was modified according to previous study to allow assessment on the quality characteristics pertaining to diagnosis intervention.

Each economic evaluation study was assessed by two reviewers. Disparities between the two reviewers were resolved through a discussion. When it could not be resolved, a third reviewer was involved.

There were four possible answers for the questions in the checklist, i.e. "Y", "N", "?", and "N/A". The answer "Y" was given when the study fulfilled the quality characteristics in question, while "N" was given when the study did not fulfill it. The answer "?" was given when there was limited amount of data or information to assess the quality characteristics in question. When the quality characteristic was regarded as not applicable to the decision problem, model type, or type of disease, the answer "N/A" was given.

References

1. Bungeţianu G, Ticău S. [Decision model regarding the discovery and diagnosis of pulmonary tuberculosis in adults]. Sante Publique (Bucur). 1979;22: 83–95.
2. Arantes GR. [Application of a mathematical model for choosing the best combination of instruments for the detection and treatment of pulmonary tuberculosis]. Rev Saude Publica. 1978;12: 455–470.
3. Bungetianu G, Plopeanu D, Siminel M, Rusu G. The radiographic (rph) method of tuberculosis detection, appraised by means of two parameters: epidemiological and economic. Sante Publ. 1976;19: 51–64.
4. Sun D, Dorman S, Shah M, Manabe, Y., Dowdy, D. Cost-effectiveness of lateral-flow urine LAM for TB diagnosis in HIV-positive South African adults. J Int AIDS Soc. 2012;15: 253–254.
5. You J, Lui G, Kam KM, Lee N. PIN83 - Xpert Mtb/Rif Assay for Rapid Diagnosis in Patients with Suspected Tuberculosis in Hong Kong - a Cost-Effectiveness Analysis. Value Health. 2014;17: A678. doi:10.1016/j.jval.2014.08.2524
6. ACTS Abstracts. Clin Transl Sci. 2014;7: 202–276. doi:10.1111/cts.12171
7. Capocci SJ, Sewell J, Smith C, Cropley I, Bhagani S, Morris S, et al. S58 Prospective Health Economic Evaluation Of Different Recommended Strategies For Tb Testing In A Contemporary Hiv Positive Cohort. Thorax. 2014;69: A32–A32. doi:10.1136/thoraxjnl-2014-206260.64
8. Pai, M., Dowdy, D, Steingart, K. Widespread abuse of serological testing for active TB in India: More costly and less effective. Am J Respir Crit Care Med. 2011;183.
9. Sun D, Dorman S, Shah M, Manabe YC, Moodley VM, Nicol MP, et al. Cost utility of lateral-flow urine lipoarabinomannan for tuberculosis diagnosis in HIV-infected African adults. Int J Tuberc Lung Dis Off J Int Union Tuberc Lung Dis. 2013;17: 552–558. doi:10.5588/ijtld.12.0627
10. You JHS, Lui G, Kam KM, Lee NLS. Cost-effectiveness analysis of the Xpert MTB/RIF assay for rapid diagnosis of suspected tuberculosis in an intermediate burden area. J Infect. 2015;70: 409–414. doi:10.1016/j.jinf.2014.12.015
11. Dowdy DW, Steingart KR, Pai M. Serological testing versus other strategies for diagnosis of active tuberculosis in India: a cost-effectiveness analysis. PLoS Med. 2011;8: e1001074. doi:10.1371/journal.pmed.1001074
12. Adelman MW, Kurbatova E, Wang YF, Leonard MK, White N, McFarland DA, et al. Cost Analysis of a Nucleic Acid Amplification Test in the Diagnosis of Pulmonary Tuberculosis at an Urban Hospital with a High Prevalence of TB/HIV. PLOS ONE. 2014;9: e100649. doi:10.1371/journal.pone.0100649
