# Supplementary material for: Disparities in model-based cost-effectiveness analyses of tuberculosis diagnosis: A systematic review
Source: PLoS One. 2018 May 9;13(5):e0193293. doi: 10.1371/journal.pone.0193293 (PMC5942841; doi:10.1371/journal.pone.0193293)
Supplement: S1 Table — (PDF) [file pone.0193293.s003.pdf]

S1 Table 1. Quality Assessment Result of The Included Studies (1)

	Authors	Kelly *et al.*,2015[1]	Little *et al.*, *2015*[2]	Suen *et al.*, *2015*[3]	You *et al.*, 2015[4]	Zwerling *et al.*, 2015[5]	Langley *et al.*, 2014[6]	Schmid *et al.*, 2014[7]	Choi *et al.*, 2013[8]	Guerra *et al*, 2013[9]	Shah *et al*, 2013[10]
**S**	**Structure**										
**S1**	**Statement of decision problem/objective**										
1	Is there a clear statement of the decision problem?	Y	Y	Y	Y	Y	Y	Y	Y	Y	Y
2	Is the objective of the evaluation and model specified and consistent with the stated decision problem?	Y	Y	Y	Y	Y	Y	Y	Y	Y	Y
3	Is the primary decision maker specified?	N	N	N	Y	N	N	N	N	N	N
**S2**	**Statement of scope/perspective**										
1	Is the perspective of the model stated clearly?	Y	Y	Y	Y	Y	Y	Y	Y	Y	Y
2	Are the model inputs consistent with the stated perspective?	Y	N	N	N	Y	Y	N	Y	Y	Y
3	Has the scope of the model been stated and justified?	Y	N	Y	Y	N	N	N	Y	N	N
4	Are the outcomes of the model consistent with the perspective, scope, and overall objective of the model?	Y	Y	Y	Y	Y	Y	Y	Y	Y	Y
**S3**	**Rationale for Structure**										
1	Has the evidence regarding model structure been described?	Y	Y	Y	N	N	Y	Y	N	Y	Y
2	Is the structure of the model consistent with a coherent theory of the health condition under evaluation?	N	N	Y	N	N	Y	N	N	N	N
3	Have any competing theories regarding model structure been considered?	N	N	N	N	N	N	N	N	N	N
4	Are the sources of data used to develop the structure of the model specified?	Y	Y	Y	N	N	Y	Y	N	Y	Y
5	Are the causal relationships described by the model structure justified appropriately?	Y	N	Y	Y	N	Y	N	Y	N	Y
**S4**	**Structural Assumption**										
1	Are the structural assumptions transparent and justified?	N	N	Y	Y	Y	Y	N	N	N	N

	Authors	Kelly *et al.*,2015[1]	Little *et al., 2015*[2]	Suen *et al., 2015*[3]	You *et al.*, 2015[4]	Zwerling *et al.*, 2015[5]	Langley *et al.*, 2014[6]	Schmid *et al.*, 2014[7]	Choi *et al.*, 2013[8]	Guerra *et al*, 2013[9]	Shah *et al*, 2013[10]
2	Are the structural assumptions reasonable given the overall objective, perspective, and scope of the model?	?	?	Y	Y	Y	Y	?	?	?	Y
**S5**	**Strategies / Comparators**										
1	Is there a clear definition of the options under evaluation?	Y	Y	Y	Y	Y	Y	N	Y	Y	Y
2	Have all feasible and practical options been evaluated?	N/A	N/A	N/A	N/A	N/A	N/A	N/A	N/A	N/A	N/A
3	Is there justification for the exclusion of feasible options?	N/A	N/A	N/A	N/A	N/A	N/A	N/A	N/A	N/A	N/A
**S6**	**Model Type**										
1	Is the chosen model type appropriate given the decision problem and specified causal relationships within the model?	Y	Y	Y	Y	Y	Y	Y	Y	Y	Y
**S7**	**Time Horizon**										
1	Is the time horizon of the model sufficient to reflect all important differences between options?	N/A	N/A	Y	N/A	N/A	Y	N/A	N/A	N/A	N/A
2	Is the time horizon of the model, the duration of treatment, and treatment effect described and justified?	N/A	N/A	Y	N/A	N/A	Y	N/A	N/A	N/A	N/A
3	Has a lifetime horizon been used?	N/A	N/A	Y	N/A	N/A	N	N/A	N/A	N/A	N/A
4	If not, has a shorter time horizon been justified?	N/A	N/A	N/A	N/A	N/A	Y	N/A	N/A	N/A	N/A
**S8**	**Disease States / Pathways**										
**1**	Do the disease states (state transition model) or the pathways (decision-tree model) reflect the underlying biological process of the disease in question and the impact of interventions?	N	N	Y	N	N	Y	N	N	N	N
**S9**	**Cycle Length**										
1	Is the cycle length defined and justified in terms of the natural history of disease?	N/A	N/A	Y	N/A	N/A	Y	N/A	N/A	N/A	N/A
**D**	**Data**										
**D1**	**Data Identification**										

	Authors	Kelly *et al.*,2015[1]	Little *et al., 2015*[2]	Suen *et al., 2015*[3]	You *et al.*, 2015[4]	Zwerling *et al.*, 2015[5]	Langley *et al.*, 2014[6]	Schmid *et al.*, 2014[7]	Choi *et al.*, 2013[8]	Guerra *et al*, 2013[9]	Shah *et al*, 2013[10]
1	Are the data identification methods transparent and appropriate given the objectives of the model?	N	N	N	Y	N	N	N	N	N	N
2	Where choices have been made between data sources, are these justified appropriately?	?	?	?	Y	?	?	?	?	?	?
3	Has particular attention been paid to identifying data for the important parameters in the model?	?	?	?	?	?	?	?	?	?	?
4	Has the process of selecting key parameters been justified and systematic methods used to identify the most appropriate data?	N	N	N	Y	N	N	N	N	N	N
5	Has the quality of the data been assessed appropriately?	?	?	?	?	?	?	?	?	?	?
6	Where expert opinion has been used, are the methods described and justified?	N/A	N/A	N/A	N/A	N/A	N/A	N/A	N/A	N/A	N/A
**D2**	**Premodel Data Analysis**										
1	Are the premodel data analysis methodologies based on justifiable statistical and epidemiological techniques?	?	?	?	?	?	?	?	?	?	?
**D2a**	**Baseline Data**										
1	Is the choice of baseline data described and justified?	?	?	?	?	?	?	?	?	?	?
2	Are transition probabilities calculated appropriately?	?	?	?	?	?	?	?	?	?	?
3	Has a half cycle correction been applied to cost and outcome?	N/A	N/A	N/A	N/A	N/A	N/A	N/A	N/A	N/A	N/A
4	If not, has this omission been justified?	N/A	N/A	N/A	N/A	N/A	N/A	N/A	N/A	N/A	N/A
**D2b**	**Treatment Effects and Diagnostic Accuracy***										
1	If relative diagnostic accuracies have been derived from trial data, have they been synthesized using appropriate techniques?*	?	?	?	?	?	?	?	?	?	?
2	Have the methods and assumptions used to extrapolate diagnostic accuracy to final outcomes been documented and justified?*	?	?	?	?	?	?	?	?	?	?

	Authors	Kelly *et al.*,2015[1]	Little *et al., 2015*[2]	Suen *et al., 2015*[3]	You *et al.*, 2015[4]	Zwerling *et al.*, 2015[5]	Langley *et al.*, 2014[6]	Schmid *et al.*, 2014[7]	Choi *et al.*, 2013[8]	Guerra *et al*, 2013[9]	Shah *et al*, 2013[10]
3	Have alternative assumptions been explored through sensitivity analysis?	N	N	Y	N	N	N	N	N	N	Y
4	Have assumptions regarding the continuing effect of treatment once treatment is complete been documented and justified?	N/A	N/A	N/A	N/A	N/A	N/A	N/A	N/A	N/A	N/A
5	Have alternative assumptions been explored through sensitivity analysis?	N/A	N/A	N/A	N/A	N/A	N/A	N/A	N/A	N/A	N/A
**D2c**	**Quality-of-Life Weights (utilities)**										
1	Are the utilities incorporated into the model appropriate?	N/A	N/A	?	N	N/A	N/A	N/A	N	N/A	N/A
2	Is the source for the utility weights referenced?	N/A	N/A	Y	Y	N/A	N/A	N/A	Y	N/A	N/A
3	Are the methods of derivation for the utility weights justified?	N/A	N/A	?	N	N/A	N/A	N/A	N	N/A	N/A
**D3**	**Data Incorporation**										
1	Have all data incorporated into the model been described and referenced in sufficient detail?	Y	Y	Y	Y	Y	Y	N	Y	N	Y
2	Has the use of mutually inconsistent data been justified (i.e., are assumptions and choices appropriate)?	N/A	N/A	N/A	N/A	N/A	N/A	N/A	N/A	N/A	N/A
3	Is the process of data incorporation transparent?	Y	Y	Y	N	Y	Y	Y	Y	N	N
4	If data have been incorporated as distributions, has the choice of distribution for each parameter been described and justified?	Y	Y	Y	N	Y	N/A	N/A	N	N/A	N
5	If data have been incorporated as distributions, is it clear that second-order uncertainty is reflected?	Y	Y	Y	Y	Y	N/A	N/A	Y	N/A	Y
**D4**	**Assessment of Uncertainty**										
1	Have the four principal types of uncertainty been addressed?	N	N	N	N	N	N	N	N	N	N
2	If not, has the omission of particular forms of uncertainty been justified?	N	N	N	N	N	N	N	N	N	N

	Authors	Kelly *et al.*,2015[1]	Little *et al., 2015*[2]	Suen *et al., 2015*[3]	You *et al.*, 2015[4]	Zwerling *et al.*, 2015[5]	Langley *et al.*, 2014[6]	Schmid *et al.*, 2014[7]	Choi *et al.*, 2013[8]	Guerra *et al*, 2013[9]	Shah *et al*, 2013[10]
**D4a**	**Methodological**										
1	Have methodological uncertainties been addressed by running alternative versions of the model with different methodological assumptions?	N	N	N	N	N	N	N	N	N	N
**D4b**	**Structural**										
1	Is there evidence that structural uncertainties have been addressed via sensitivity analysis?	N	N	N	N	N	N	N	N	N	N
**D4c**	**Heterogeneity**										
1	Has heterogeneity been dealt with by running the model separately for different subgroups?	N	N	Y	N	N	N	N	N	N	Y
**D4d**	**Parameter**										
1	Are the methods of assessment of parameter uncertainty appropriate?	Y	Y	Y	Y	Y	Y	N	Y	N	Y
2	Has probabilistic sensitivity analysis been done? If not, has this been justified?	Y	Y	Y	Y	Y	N	N	Y	N	Y
3	If data are incorporated as point estimates, are the ranges used for sensitivity analysis stated clearly and justified?	Y	Y	Y	Y	Y	Y	N	N	N	N
**C**	**Consistency**										
**C1**	**Internal Consistency**										
1	Is there evidence that the mathematical logic of the model has been tested thoroughly before use?	N	N	Y	N	N	Y	N	N	N	N
**C2**	**External Consistency**										
1	Are the conclusions valid given the data presented?	Y	Y	Y	Y	Y	Y	N	Y	Y	Y
2	Are any counterintuitive results from the model explained and justified?	N/A	N/A	N/A	N/A	N/A	N/A	N/A	N/A	N/A	N/A
3	If the model has been calibrated against independent data, have any differences been explained and justified?	N/A	N/A	Y	N/A	N/A	Y	N/A	N/A	N/A	N/A
4	Have the results of the model been compared with those of previous models and any differences in results explained?	Y	N	N	N	Y	Y	N	N	Y	Y

S1 Table 2. Quality Assessment Result of The Included Studies (2)

	Authors	Sun *et al.*, 2013[11]	van't Hoog *et al.*, 2013[12]	Abimbola *et al.*, 2012[13]	Menzies *et al.*, 2012[14]	Dowdy *et al.*, 2011[15]	Hughes *et al.*, 2011[16]	Vassal *et al.*, 2011[17]	Chihota *et al.*, 2010[18]	Bonnet *et al.*, 2010[19]	Scherer *et al.*, 2009[20]
**S**	**Structure**										
**S1**	**Statement of decision problem/objective**										
1	Is there a clear statement of the decision problem?	Y	Y	Y	Y	Y	Y	Y	Y	Y	Y
2	Is the objective of the evaluation and model specified and consistent with the stated decision problem?	Y	Y	Y	Y	Y	Y	Y	Y	Y	Y
3	Is the primary decision maker specified?	N	N	N	N	N	Y	N	Y	Y	N
**S2**	**Statement of scope/perspective**										
1	Is the perspective of the model stated clearly?	Y	Y	Y	Y	Y	Y	Y	Y	Y	N
2	Are the model inputs consistent with the stated perspective?	N	Y	Y	Y	Y	N	Y	Y	N	N/A
3	Has the scope of the model been stated and justified?	N	N	N	Y	N	Y	N	N	N	N
4	Are the outcomes of the model consistent with the perspective, scope, and overall objective of the model?	Y	Y	Y	Y	Y	Y	Y	Y	Y	N/A
**S3**	**Rationale for Structure**										
1	Has the evidence regarding model structure been described?	Y	Y	N	Y	Y	N	Y	N	N	N
2	Is the structure of the model consistent with a coherent theory of the health condition under evaluation?	N	N	N	Y	N	N	N	N	N	N
3	Have any competing theories regarding model structure been considered?	N	N	N	N	N	N	N	N	N	N
4	Are the sources of data used to develop the structure of the model specified?	Y	Y	N	Y	Y	N	Y	N	N	N

	Authors	Sun *et al.*, 2013[11]	van't Hoog *et al.*, 2013[12]	Abimbola *et al.*, 2012[13]	Menzies *et al.*, 2012[14]	Dowdy *et al.*, 2011[15]	Hughes *et al.*, 2011[16]	Vassal *et al.*, 2011[17]	Chihota *et al.*, 2010[18]	Bonnet *et al.*, 2010[19]	Scherer *et al.*, 2009[20]
5	Are the causal relationships described by the model structure justified appropriately?	Y	Y	N	Y	Y	Y	Y	N	N	N
**S4**	**Structural Assumption**										
1	Are the structural assumptions transparent and justified?	N	Y	N	Y	N	Y	Y	N	N	N
2	Are the structural assumptions reasonable given the overall objective, perspective, and scope of the model?	?	Y	?	Y	?	Y	Y	?	?	?
**S5**	**Strategies / Comparators**										
1	Is there a clear definition of the options under evaluation?	N	Y	Y	Y	Y	Y	Y	Y	Y	Y
2	Have all feasible and practical options been evaluated?	N/A	N/A	N/A	N/A	N/A	N/A	N/A	N/A	N/A	N/A
3	Is there justification for the exclusion of feasible options?	N/A	N/A	N/A	N/A	N/A	N/A	N/A	N/A	N/A	N/A
**S6**	**Model Type**										
1	Is the chosen model type appropriate given the decision problem and specified causal relationships within the model?	Y	Y	Y	Y	Y	Y	Y	Y	Y	Y
**S7**	**Time Horizon**										
1	Is the time horizon of the model sufficient to reflect all important differences between options?	N/A	N/A	N/A	Y	N/A	N/A	N/A	N/A	N/A	N/A
2	Is the time horizon of the model, the duration of treatment, and treatment effect described and justified?	N/A	N/A	N/A	Y	N/A	N/A	N/A	N/A	N/A	N/A
3	Has a lifetime horizon been used?	N/A	N/A	N/A	N	N/A	N/A	N/A	N/A	N/A	N/A
4	If not, has a shorter time horizon been justified?	N/A	N/A	N/A	Y	N/A	N/A	N/A	N/A	N/A	N/A
**S8**	**Disease States / Pathways**										
**1**	Do the disease states (state transition model) or the pathways (decision-tree model) reflect the underlying biological process of the disease in question and the impact of interventions?	N	N	N	Y	N	N	N	N	N	N

	Authors	Sun *et al.*, 2013[11]	van't Hoog *et al.*, 2013[12]	Abimbola *et al.*, 2012[13]	Menzies *et al.*, 2012[14]	Dowdy *et al.*, 2011[15]	Hughes *et al.*, 2011[16]	Vassal *et al.*, 2011[17]	Chihota *et al.*, 2010[18]	Bonnet *et al.*, 2010[19]	Scherer *et al.*, 2009[20]
**S9**	**Cycle Length**										
1	Is the cycle length defined and justified in terms of the natural history of disease?	N/A	N/A	N/A	Y	N/A	N/A	N/A	N/A	N/A	N/A
**D**	**Data**										
**D1**	**Data Identification**										
1	Are the data identification methods transparent and appropriate given the objectives of the model?	N	N	N	N	N	Y	N	N	N	N
2	Where choices have been made between data sources, are these justified appropriately?	?	?	?	?	?	?	?	?	?	?
3	Has particular attention been paid to identifying data for the important parameters in the model?	?	?	?	?	?	?	?	?	?	?
4	Has the process of selecting key parameters been justified and systematic methods used to identify the most appropriate data?	N	N	N	N	N	Y	N	N	N	N
5	Has the quality of the data been assessed appropriately?	?	?	?	?	?	?	?	?	?	?
6	Where expert opinion has been used, are the methods described and justified?	N/A	N/A	N/A	N/A	N/A	N	N/A	N/A	N/A	N/A
**D2**	**Premodel Data Analysis**										
1	Are the premodel data analysis methodologies based on justifiable statistical and epidemiological techniques?	?	?	?	?	?	?	?	?	?	?
**D2a**	**Baseline Data**										
1	Is the choice of baseline data described and justified?	?	?	?	Y	?	?	?	?	?	?
2	Are transition probabilities calculated appropriately?	?	?	?	Y	?	?	?	?	?	?
3	Has a half cycle correction been applied to cost and outcome?	N/A	N/A	N/A	N/A	N/A	N/A	N/A	N/A	N/A	N/A
4	If not, has this omission been justified?	N/A	N/A	N/A	N/A	N/A	N/A	N/A	N/A	N/A	N/A
**D2b**	**Treatment Effects and Diagnostic Accuracy***										

	Authors	Sun *et al.*, 2013[11]	van't Hoog *et al.*, 2013[12]	Abimbola *et al.*, 2012[13]	Menzies *et al.*, 2012[14]	Dowdy *et al.*, 2011[15]	Hughes *et al.*, 2011[16]	Vassal *et al.*, 2011[17]	Chihota *et al.*, 2010[18]	Bonnet *et al.*, 2010[19]	Scherer *et al.*, 2009[20]
1	If relative diagnostic accuracies have been derived from trial data, have they been synthesized using appropriate techniques?*	?	?	?	?	?	?	?	?	?	?
2	Have the methods and assumptions used to extrapolate diagnostic accuracy to final outcomes been documented and justified?*	?	?	?	?	?	?	?	?	?	?
3	Have alternative assumptions been explored through sensitivity analysis?	Y	Y	Y	Y	N	N	Y	N	N	N
4	Have assumptions regarding the continuing effect of treatment once treatment is complete been documented and justified?	N/A	N/A	N/A	N/A	N/A	N/A	N/A	N/A	N/A	N/A
5	Have alternative assumptions been explored through sensitivity analysis?	N/A	N/A	N/A	N/A	N/A	N/A	N/A	N/A	N/A	N/A
**D2c**	**Quality-of-Life Weights (utilities)**										
1	Are the utilities incorporated into the model appropriate?	N/A	N/A	N/A	N/A	N/A	Y	N/A	N/A	N/A	N/A
2	Is the source for the utility weights referenced?	N/A	N/A	N/A	N/A	N/A	Y	N/A	N/A	N/A	N/A
3	Are the methods of derivation for the utility weights justified?	N/A	N/A	N/A	N/A	N/A	Y	N/A	N/A	N/A	N/A
**D3**	**Data Incorporation**										
1	Have all data incorporated into the model been described and referenced in sufficient detail?	Y	Y	Y	Y	Y	Y	Y	N	N	Y
2	Has the use of mutually inconsistent data been justified (i.e., are assumptions and choices appropriate)?	N/A	N/A	N/A	N/A	N/A	N/A	N/A	N/A	N/A	N/A
3	Is the process of data incorporation transparent?	Y	Y	Y	Y	Y	Y	Y	N	N	Y
4	If data have been incorporated as distributions, has the choice of distribution for each parameter been described and justified?	Y	Y	Y	Y	N/A	Y	Y	N/A	N/A	N/A
5	If data have been incorporated as distributions, is it clear that second-order uncertainty is reflected?	Y	Y	Y	Y	N/A	Y	Y	N/A	N/A	N/A
**D4**	**Assessment of Uncertainty**										

	Authors	Sun *et al.*, 2013[11]	van't Hoog *et al.*, 2013[12]	Abimbola *et al.*, 2012[13]	Menzies *et al.*, 2012[14]	Dowdy *et al.*, 2011[15]	Hughes *et al.*, 2011[16]	Vassal *et al.*, 2011[17]	Chihota *et al.*, 2010[18]	Bonnet *et al.*, 2010[19]	Scherer *et al.*, 2009[20]
1	Have the four principal types of uncertainty been addressed?	N	N	N	N	N	N	N	N	N	N
2	If not, has the omission of particular forms of uncertainty been justified?	N	N	N	N	N	N	N	N	N	N
**D4a**	**Methodological**										
1	Have methodological uncertainties been addressed by running alternative versions of the model with different methodological assumptions?	N	N	N	N	N	N	N	N	N	N
**D4b**	**Structural**										
1	Is there evidence that structural uncertainties have been addressed via sensitivity analysis?	N	N	N	N	N	N	N	N	N	N
**D4c**	**Heterogeneity**										
1	Has heterogeneity been dealt with by running the model separately for different subgroups?	N	Y	N	N	N	N	Y	N	N	Y
**D4d**	**Parameter**										
1	Are the methods of assessment of parameter uncertainty appropriate?	Y	Y	Y	Y	N	Y	Y	N	N	N
2	Has probabilistic sensitivity analysis been done? If not, has this been justified?	Y	Y	Y	Y	N	Y	Y	N	N	N
3	If data are incorporated as point estimates, are the ranges used for sensitivity analysis stated clearly and justified?	Y	Y	Y	Y	Y	Y	Y	N	N	N
**C**	**Consistency**										
**C1**	**Internal Consistency**										
1	Is there evidence that the mathematical logic of the model has been tested thoroughly before use?	N	N	N	Y	N	N	N	N	N	N
**C2**	**External Consistency**										
1	Are the conclusions valid given the data presented?	Y	Y	Y	Y	Y	Y	Y	Y	Y	Y
2	Are any counterintuitive results from the model	N/A	N/A	N/A	N/A	N/A	N/A	Y	N/A	N/A	N/A

	Authors	Sun *et al.*, 2013[11]	van't Hoog *et al.*, 2013[12]	Abimbola *et al.*, 2012[13]	Menzies *et al.*, 2012[14]	Dowdy *et al.*, 2011[15]	Hughes *et al.*, 2011[16]	Vassal *et al.*, 2011[17]	Chihota *et al.*, 2010[18]	Bonnet *et al.*, 2010[19]	Scherer *et al.*, 2009[20]
	explained and justified?										
3	If the model has been calibrated against independent data, have any differences been explained and justified?	N/A	N/A	N/A	Y	N/A	N/A	N/A	N/A	N/A	N/A
4	Have the results of the model been compared with those of previous models and any differences in results explained?	N	N	N	Y	N	Y	N/A	N	N	Y

S1 Table 3. Quality Assessment Result of The Included Studies (3)

	Authors	Dowdy *et al.*, 2008 (1)[21]	Dowdy *et al.*, 2008 (2)[22]	Guerra *et al.*, 2008[23]	Mueller *et al.*, 2008[24]	Rajahlati *et al.*, 2004[25]	Dowdy *et al.*, 2003[26]	Roos *et al.*, 1998[27]
**S**	**Structure**							
**S1**	**Statement of decision problem/objective**							
1	Is there a clear statement of the decision problem?	Y	Y	Y	Y	Y	Y	Y
2	Is the objective of the evaluation and model specified and consistent with the stated decision problem?	Y	Y	Y	Y	Y	Y	Y
3	Is the primary decision maker specified?	N	N	N	N	N	Y	N
**S2**	**Statement of scope/perspective**							
1	Is the perspective of the model stated clearly?	Y	Y	Y	Y	N	Y	N
2	Are the model inputs consistent with the stated perspective?	N	N	N	Y	N/A	N	N
3	Has the scope of the model been stated and justified?	N	N	N	N	N	Y	N
4	Are the outcomes of the model consistent with the perspective, scope, and overall objective of the model?	Y	Y	Y	Y	N/A	Y	N
**S3**	**Rationale for Structure**							
1	Has the evidence regarding model structure been described?	N	N	N	N	Y	N	N
2	Is the structure of the model consistent with a coherent theory of the health condition under evaluation?	N	N	N	N	N	N	N
3	Have any competing theories regarding model structure been considered?	N	N	N	N	N	N	N
4	Are the sources of data used to develop the structure of the model specified?	N	N	Y	N	Y	N	N
5	Are the causal relationships described by the model structure justified appropriately?	N	Y	N	N	Y	N	N
**S4**	**Structural Assumption**							
1	Are the structural assumptions transparent and justified?	N	Y	N	N	Y	N	N
2	Are the structural assumptions reasonable given the overall objective, perspective, and scope of the model?	?	Y	?	?	Y	?	?
**S5**	**Strategies / Comparators**							
1	Is there a clear definition of the options under evaluation?	Y	Y	N	Y	Y	Y	N
2	Have all feasible and practical options been evaluated?	N/A	N/A	N/A	N/A	N/A	N/A	N/A
3	Is there justification for the exclusion of feasible options?	N/A	N/A	N/A	N/A	N/A	N/A	N/A

	Authors	Dowdy *et al.*, 2008 (1)[21]	Dowdy *et al.*, 2008 (2)[22]	Guerra *et al.*, 2008[23]	Mueller *et al.*, 2008[24]	Rajahlati *et al.*, 2004[25]	Dowdy *et al.*, 2003[26]	Roos *et al.*, 1998[27]
**S6**	**Model Type**							
1	Is the chosen model type appropriate given the decision problem and specified causal relationships within the model?	Y	Y	Y	?	Y	Y	?
**S7**	**Time Horizon**							
1	Is the time horizon of the model sufficient to reflect all important differences between options?	Y	N/A	N/A	N/A	N/A	N/A	N/A
2	Is the time horizon of the model, the duration of treatment, and treatment effect described and justified?	Y	N/A	N/A	N/A	N/A	N/A	N/A
3	Has a lifetime horizon been used?	Y	N/A	N/A	N/A	N/A	N/A	N/A
4	If not, has a shorter time horizon been justified?	N/A	N/A	N/A	N/A	N/A	N/A	N/A
**S8**	**Disease States / Pathways**							
**1**	Do the disease states (state transition model) or the pathways (decision-tree model) reflect the underlying biological process of the disease in question and the impact of interventions?	N	N	N	N	N	N	N
**S9**	**Cycle Length**							
1	Is the cycle length defined and justified in terms of the natural history of disease?	N	N/A	N/A	N/A	N/A	N/A	N/A
**D**	**Data**							
**D1**	**Data Identification**							
1	Are the data identification methods transparent and appropriate given the objectives of the model?	N	N	N	N	Y	N	N
2	Where choices have been made between data sources, are these justified appropriately?	?	?	?	?	?	?	?
3	Has particular attention been paid to identifying data for the important parameters in the model?	?	?	?	?	?	?	?
4	Has the process of selecting key parameters been justified and systematic methods used to identify the most appropriate data?	N	N	N	N	N	N	N
5	Has the quality of the data been assessed appropriately?	?	?	?	?	?	?	?
6	Where expert opinion has been used, are the methods described and justified?	N/A	N/A	N/A	N/A	N/A	N/A	N
**D2**	**Premodel Data Analysis**							

	Authors	Dowdy *et al.*, 2008 (1)[21]	Dowdy *et al.*, 2008 (2)[22]	Guerra *et al.*, 2008[23]	Mueller *et al.*, 2008[24]	Rajahlati *et al.*, 2004[25]	Dowdy *et al.*, 2003[26]	Roos *et al.*, 1998[27]
1	Are the premodel data analysis methodologies based on justifiable statistical and epidemiological techniques?	?	?	?	?	?	?	?
**D2a**	**Baseline Data**							
1	Is the choice of baseline data described and justified?	?	?	?	?	?	?	?
2	Are transition probabilities calculated appropriately?	?	?	?	?	?	?	?
3	Has a half cycle correction been applied to cost and outcome?	N	N/A	N/A	N/A	N/A	N/A	N/A
4	If not, has this omission been justified?	N	N/A	N/A	N/A	N/A	N/A	N/A
**D2b**	**Treatment Effects and Diagnostic Accuracy***							
1	If relative diagnostic accuracies have been derived from trial data, have they been synthesized using appropriate techniques?*	?	?	?	?	?	?	?
2	Have the methods and assumptions used to extrapolate diagnostic accuracy to final outcomes been documented and justified?*	?	?	?	?	?	?	?
3	Have alternative assumptions been explored through sensitivity analysis?	N	N	Y	N	N	N	N
4	Have assumptions regarding the continuing effect of treatment once treatment is complete been documented and justified?	N/A	N/A	N/A	N/A	N/A	N/A	N/A
5	Have alternative assumptions been explored through sensitivity analysis?	N/A	N/A	N/A	N/A	N/A	N/A	N/A
**D2c**	**Quality-of-Life Weights (utilities)**							
1	Are the utilities incorporated into the model appropriate?	N/A	N/A	N/A	N/A	N/A	N/A	N/A
2	Is the source for the utility weights referenced?	N/A	N/A	N/A	N/A	N/A	N/A	N/A
3	Are the methods of derivation for the utility weights justified?	N/A	N/A	N/A	N/A	N/A	N/A	N/A
**D3**	**Data Incorporation**							
1	Have all data incorporated into the model been described and referenced in sufficient detail?	Y	Y	Y	Y	Y	N	N
2	Has the use of mutually inconsistent data been justified (i.e., are assumptions and choices appropriate)?	N/A	N/A	N/A	N/A	N/A	N/A	N/A
3	Is the process of data incorporation transparent?	Y	Y	N	N	Y	N	N
4	If data have been incorporated as distributions, has the choice of distribution for each parameter been described and justified?	Y	N/A	N/A	N/A	N/A	N/A	N/A
5	If data have been incorporated as distributions, is it clear that second-order uncertainty is reflected?	Y	N/A	N/A	N/A	N/A	N/A	N/A

	Authors	Dowdy *et al.*, 2008 (1)[21]	Dowdy *et al.*, 2008 (2)[22]	Guerra *et al.*, 2008[23]	Mueller *et al.*, 2008[24]	Rajahlati *et al.*, 2004[25]	Dowdy *et al.*, 2003[26]	Roos *et al.*, 1998[27]
**D4**	**Assessment of Uncertainty**							
1	Have the four principal types of uncertainty been addressed?	N	N	N	N	N	N	N
2	If not, has the omission of particular forms of uncertainty been justified?	N	N	N	N	N	N	N
**D4a**	**Methodological**							
1	Have methodological uncertainties been addressed by running alternative versions of the model with different methodological assumptions?	N	N	N	N	N	N	N
**D4b**	**Structural**							
1	Is there evidence that structural uncertainties have been addressed via sensitivity analysis?	N	N	N	N	N	N	N
**D4c**	**Heterogeneity**							
1	Has heterogeneity been dealt with by running the model separately for different subgroups?	N	Y	N	N	N	N	N
**D4d**	**Parameter**							
1	Are the methods of assessment of parameter uncertainty appropriate?	Y	Y	Y	N	Y	Y	N
2	Has probabilistic sensitivity analysis been done? If not, has this been justified?	Y	Y	N	N	N	N	N
3	If data are incorporated as point estimates, are the ranges used for sensitivity analysis stated clearly and justified?	Y	Y	Y	N	N	N	N
**C**	**Consistency**							
**C1**	**Internal Consistency**							
1	Is there evidence that the mathematical logic of the model has been tested thoroughly before use?	N	N	N	N	N	N	N
**C2**	**External Consistency**							
1	Are the conclusions valid given the data presented?	Y	Y	Y	Y	Y	N	N
2	Are any counterintuitive results from the model explained and justified?	N/A	Y	N/A	N/A	N/A	N/A	N/A
3	If the model has been calibrated against independent data, have any differences been explained and justified?	N	N/A	N/A	N/A	N/A	N/A	N/A

	Authors	Dowdy *et al.*, 2008 (1)[21]	Dowdy *et al.*, 2008 (2)[22]	Guerra *et al.*, 2008[23]	Mueller *et al.*, 2008[24]	Rajahlati *et al.*, 2004[25]	Dowdy *et al.*, 2003[26]	Roos *et al.*, 1998[27]
4	Have the results of the model been compared with those of previous models and any differences in results explained?	Y	N	N	N	N	N	N

References

1. Kelly V, Sagili KD, Satyanarayana S, Reza LW, Chadha SS, Wilson NC. Cost-utility analysis of LED fluorescence microscopy in the diagnosis of pulmonary tuberculosis in Indian settings. Int J Tuberc Lung Dis Off J Int Union Tuberc Lung Dis. 2015;19: 696–701. doi:10.5588/ijtld.14.0203

2. Little KM, Pai M, Dowdy DW. Costs and Consequences of Using Interferon-γ Release Assays for the Diagnosis of Active Tuberculosis in India. PloS One. 2014;10: e0124525. doi:10.1371/journal.pone.0124525

3. Suen S-C, Bendavid E, Goldhaber-Fiebert JD. Cost-effectiveness of improvements in diagnosis and treatment accessibility for tuberculosis control in India. Int J Tuberc Lung Dis Off J Int Union Tuberc Lung Dis. 2015;19: 1115–1124, i–xv. doi:10.5588/ijtld.15.0158

4. You JHS, Lui G, Kam KM, Lee NLS. Cost-effectiveness analysis of the Xpert MTB/RIF assay for rapid diagnosis of suspected tuberculosis in an intermediate burden area. J Infect. 2015;70: 409–414. doi:10.1016/j.jinf.2014.12.015

5. Zwerling AA, Sahu M, Ngwira LG, Khundi M, Harawa T, Corbett EL, et al. Screening for tuberculosis among adults newly diagnosed with HIV in sub-Saharan Africa: a cost-effectiveness analysis. J Acquir Immune Defic Syndr 1999. 2015; doi:10.1097/QAI.0000000000000712

6. Langley I, Lin H-H, Egwaga S, Doulla B, Ku C-C, Murray M, et al. Assessment of the patient, health system, and population effects of Xpert MTB/RIF and alternative diagnostics for tuberculosis in Tanzania: an integrated modelling approach. Lancet Glob Health. 2014;2: e581-591. doi:10.1016/S2214-109X(14)70291-8

7. Schmid KB, Scherer L, Barcellos RB, Kuhleis D, Prestes IV, Steffen RE, et al. Smear plus Detect-TB for a sensitive diagnosis of pulmonary tuberculosis: a cost-effectiveness analysis in an incarcerated population. BMC Infect Dis. 2014;14: 678. doi:10.1186/s12879-014-0678-x

8. Choi HW, Miele K, Dowdy D, Shah M. Cost-effectiveness of Xpert® MTB/RIF for diagnosing pulmonary tuberculosis in the United States. Int J Tuberc Lung Dis Off J Int Union Tuberc Lung Dis. 2013;17: 1328–1335. doi:10.5588/ijtld.13.0095

9. Guerra RL, Dorman SE, Luiz RR, Conde MB. Cost-effectiveness of routine diagnostic evaluation of pulmonary tuberculosis in a primary care unit in Brazil. Int J Tuberc Lung Dis Off J Int Union Tuberc Lung Dis. 2013;17: 1336–1340. doi:10.5588/ijtld.13.0073

10. Shah M, Dowdy D, Joloba M, Ssengooba W, Manabe YC, Ellner J, et al. Cost-effectiveness of novel algorithms for rapid diagnosis of tuberculosis in HIV-infected individuals in Uganda. AIDS Lond Engl. 2013;27: 2883–2892. doi:10.1097/QAD.0000000000000008

11. Sun D, Dorman S, Shah M, Manabe YC, Moodley VM, Nicol MP, et al. Cost utility of lateral-flow urine lipoarabinomannan for tuberculosis diagnosis in HIV-infected African adults. Int J Tuberc Lung Dis Off J Int Union Tuberc Lung Dis. 2013;17: 552–558. doi:10.5588/ijtld.12.0627

12. van't Hoog AH, Cobelens F, Vassall A, van Kampen S, Dorman SE, Alland D, et al. Optimal triage test characteristics to improve the cost-effectiveness of the Xpert MTB/RIF assay for TB diagnosis: a decision analysis. PloS One. 2013;8: e82786. doi:10.1371/journal.pone.0082786

13. Abimbola TO, Marston BJ, Date AA, Blandford JM, Sangrujee N, Wiktor SZ. Cost-effectiveness of tuberculosis diagnostic strategies to reduce early mortality among persons with advanced HIV infection initiating antiretroviral therapy. J Acquir Immune Defic Syndr 1999. 2012;60: e1-7. doi:10.1097/QAI.0b013e318246538f

14. Menzies NA, Cohen T, Lin H-H, Murray M, Salomon JA. Population health impact and cost-effectiveness of tuberculosis diagnosis with Xpert MTB/RIF: a dynamic simulation and economic evaluation. PLoS Med. 2012;9: e1001347. doi:10.1371/journal.pmed.1001347

15. Dowdy DW, Steingart KR, Pai M. Serological testing versus other strategies for diagnosis of active tuberculosis in India: a cost-effectiveness analysis. PLoS Med. 2011;8: e1001074. doi:10.1371/journal.pmed.1001074

16. Hughes R, Wonderling D, Li B, Higgins B. The cost effectiveness of Nucleic Acid Amplification Techniques for the diagnosis of tuberculosis. Respir Med. 2012;106: 300–307. doi:10.1016/j.rmed.2011.10.005

17. Vassall A, van Kampen S, Sohn H, Michael JS, John KR, den Boon S, et al. Rapid diagnosis of tuberculosis with the Xpert MTB/RIF assay in high burden countries: a cost-effectiveness analysis. PLoS Med. 2011;8: e1001120. doi:10.1371/journal.pmed.1001120

18. Chihota VN, Grant AD, Fielding K, Ndibongo B, van Zyl A, Muirhead D, et al. Liquid vs. solid culture for tuberculosis: performance and cost in a resource-constrained setting. Int J Tuberc Lung Dis Off J Int Union Tuberc Lung Dis. 2010;14: 1024–1031.

19. Bonnet M, Tajahmady A, Hepple P, Ramsay A, Githui W, Gagdnidze L, et al. Added value of bleach sedimentation microscopy for diagnosis of tuberculosis: a cost-effectiveness study. Int J Tuberc Lung Dis Off J Int Union Tuberc Lung Dis. 2010;14: 571–577.

20. Scherer LC, Sperhacke RD, Ruffino-Netto A, Rossetti ML, Vater C, Klatser P, et al. Cost-effectiveness analysis of PCR for the rapid diagnosis of pulmonary tuberculosis. BMC Infect Dis. 2009;9: 216. doi:10.1186/1471-2334-9-216

21. Dowdy DW, Lourenço MC, Cavalcante SC, Saraceni V, King B, Golub JE, et al. Impact and cost-effectiveness of culture for diagnosis of tuberculosis in HIV-infected Brazilian adults. PloS One. 2008;3: e4057. doi:10.1371/journal.pone.0004057

22. Dowdy DW, O'Brien MA, Bishai D. Cost-effectiveness of novel diagnostic tools for the diagnosis of tuberculosis. Int J Tuberc Lung Dis Off J Int Union Tuberc Lung Dis. 2008;12: 1021–1029.

23. Guerra RL, Hooper NM, Baker JF, Alborz R, Armstrong DT, Kiehlbauch JA, et al. Cost-effectiveness of different strategies for amplified Mycobacterium tuberculosis direct testing for cases of pulmonary tuberculosis. J Clin Microbiol. 2008;46: 3811–3812. doi:10.1128/JCM.01682-08

24. Mueller DH, Mwenge L, Muyoyeta M, Muvwimi MW, Tembwe R, McNerney R, et al. Costs and cost-effectiveness of tuberculosis cultures using solid and liquid media in a developing country. Int J Tuberc Lung Dis Off J Int Union Tuberc Lung Dis. 2008;12: 1196–1202.

25. Rajalahti I, Ruokonen EL, Kotomäki T, Sintonen H, Nieminen MM. Economic evaluation of the use of PCR assay in diagnosing pulmonary TB in a low-incidence area. Eur Respir J. 2004;23: 446–451.

26. Dowdy DW, Maters A, Parrish N, Beyrer C, Dorman SE. Cost-Effectiveness Analysis of the Gen-Probe Amplified Mycobacterium Tuberculosis Direct Test as Used Routinely on Smear-Positive Respiratory Specimens. J Clin Microbiol. 2003;41: 948–953. doi:10.1128/JCM.41.3.948-953.2003

27. Roos BR, van Cleeff MR, Githui WA, Kivihya-Ndugga L, Odhiambo JA, Kibuga DK, et al. Cost-effectiveness of the polymerase chain reaction versus smear examination for the diagnosis of tuberculosis in Kenya: a theoretical model. Int J Tuberc Lung Dis Off J Int Union Tuberc Lung Dis. 1998;2: 235–241.
