# Supplementary material for: Disparities in model-based cost-effectiveness analyses of tuberculosis diagnosis: A systematic review
Source: PLoS One. 2018 May 9;13(5):e0193293. doi: 10.1371/journal.pone.0193293 (PMC5942841; doi:10.1371/journal.pone.0193293)
Supplement: S2 Table — (PDF) [file pone.0193293.s004.pdf]

S2 Table 1. Data Extraction for General Information (1)

Information \ Author		Kelly *et al.*[1]	Little *et al.*[2]	Suen *et al.*[3]	You *et al.*[4]	Zwerling *et al.*[5]	Langley *et al.*[6]
Type of PE Analysis		Cost-utility Analysis	Cost-utility Analysis	Cost-utility Analysis	Cost-utility Analysis	Cost-utility Analysis	Cost-utility Analysis
Publication Year		2015	2015	2015	2015	2015	2014
Country		India	India	India	PR China, Hong Kong	Malawi	Tanzania
Objective		To compare the cost and benefit of employing different smear microscopy tools for smear positive Tuberculosis detection	To estimate the cost and consequences of utilizing IGRA for active pulmonary TB diagnosis in adults	To evaluate the cost effectiveness of Xpert and Public-Private mix (PPM), used alone or in combination	To evaluate the potential economic and clinical outcomes of inpatient TB presumptive cases rapid diagnosis with Xpert from the perspective of Hong Kong healthcare providers	To explore the cost-effectiveness of TB on-demand screening in low-income countries of sub-Saharan Africa (screening was performed on patients with any TB symptoms)	To assess the effects of new WHO endorsed diagnosis options (Xpert and light emitting diode (LED) fluorescence microscopy) at patient, health system, and population level
Type of Model (as stated in the study)		Decision Tree Model	Decision Analysis Model	Dynamic Transmission Microsimulation Model	Decision Tree Model	Decision Analytic Model	Discrete-event Simulation (operational component) linked with epidemic model / dynamic transmission model
Perspective		Health System (TB program)	Health system (private and public TB-control sector)	Societal	Health service providers	Health system (TB Program)	Health system
Base/Reference Case		Ziehl Neesen (ZN) microscopy	Existing standard care for TB diagnosis without microbiology test (e.g. smear microscopy)	Fragmented public and private TB care provider, sputum smear microscopy (SSM) as initial test, as well as culture and Drug Sensitivity Testing (DST) for MDR TB testing	Sputum smear microscopy, followed by clinical diagnosis including empirical treatment for smear negative cases	Ziehl Neesen (ZN) smear microscopy based on physician's discretion	Ziehl Neesen (ZN) smear microscopy
Comparative Strategies	1	LED fluorescence microscopy	Base case augmented with smear microscopy	Xpert for DST (SSM as initial test)	Sputum smear microscopy, followed by Xpert testing for smear negative cases	Patients having at least one TB symptom was diagnosed using LED fluorescence microscopy	Replace ZN microscopy with LED fluorescence microscopy
	2		Base case augmented with IGRA	Xpert for initial diagnosis and DST, only in public sector health center	All patients tested with single sputum Xpert.	Patients having at least one TB symptom was diagnosed using Xpert	LED fluorescence microscopy with 2 sputum samples collected on the same day
	3		Base case augmented with Xpert	PPM (referral of TB suspect from private provider to public sector health center).			Xpert for all presumptive TB patient (full roll-out)
	4		Base case augmented with mycobacterial culture (not discussed extensively since culture has no significant impact on treatment decision)	PPM combined with Xpert for DST			Xpert for known HIV-positive cases
	5			PPM combined with Xpert for initial diagnosis and DST			Xpert for HIV-positive cases with additional HIV testing
	6						Xpert for smear-negative and known HIV-positive cases
	7						Xpert for smear-negative and HIV-positive cases with additional HIV testing
Study population		Cohort of adult with presumptive TB, presenting in the study sites	One million Indian adults with presumptive active pulmonary TB (hypothetical cohort)	Representative of the entire population of India (all ages, followed from birth until death)	Hypothetical cohort adult patients hospitalized for presumptive active PTB based on symptom and Chest X-Ray	People receiving a new HIV diagnosis	Patient with presumptive TB (adult active pulmonary TB), with known and unknown HIV status

Information		Author: Kelly *et al.*[1]	Little *et al.*[2]	Suen *et al.*[3]	You *et al.*[4]	Zwerling *et al.*[5]	Langley *et al.*[6]
Type of Cost Incorporated	Direct	Diagnosis and treatment cost	Diagnosis and treatment cost (including empirical treatment cost and MDR treatment cost in the Xpert arm; no inclusion of HIV treatment cost)	TB specific cost : diagnosis and treatment; non-TB health-related cost (age and sex specific background medical expenditure)	Diagnosis (including cost for empirical treatment) and treatment cost (including second line TB treatment; excluding hospitalization cost)	Diagnosis and treatment cost (including HIV treatment cost)	Diagnosis (investment and running cost) and treatment cost (standard and MDR TB, as well as HIV treatment cost)
	Indirect	Overhead cost (associated with diagnostics)				Overhead cost	Overhead cost was assumed to be unaffected by the change of diagnostic algorithm
Health Outcomes Measures		DALY, case detected	DALY averted (*no age weighting)*, number of secondary transmission (calculated with certain assumptions from undiagnosed patients), number of false positive cases diagnosed, number of TB case (true positive) treated, and deaths	QALY, TB prevalence, Incidence	One year mortality rate, QALY gained	DALY averted (*no age weighting*)	DALY averted (*no age weighting)*, number of TB events averted, and additional number of people on ART
Discount Rate (per year)		Undiscounted (short term analysis)	3%	3%	3%	3%	3%
Time horizon (as stated in the report)		1 year	N/A	Lifetime	N/A	ART costs and DALY was calculated over patients' lifetime.	10 years
Analysis of Parameter uncertainty		Univariate and PSA	Univariate, multivariate (two-way sensitivity analysis), PSA	Univariate, Multivariate, PSA	Univariate and PSA	Univariate, multivariate (two-way sensitivity analysis, and PSA	Univariate
Main Result		LED fluorescence microscopy resulted in a higher total cost, but also detected more TB cases which caused more DALYs averted.	1. IGRA yielded similar number of true positive as Xpert, while producing more false positive (3.3 false positive diagnosed for every true positive).<br>2. As a consequence, healthcare cost for IGRA was higher than other diagnosis arms 3. PSA : IGRA was more costly than Xpert (including MDR treatment) in 89% of the simulations	1. All intervention reduce future TB incidence and prevalence compare to status quo. Combination of Xpert and PPM resulted in the highest reduction.<br>3. Xpert and PPM produced almost the same amount of case notification; at a lower cost for PPM. Therefore PPM dominated Xpert options.<br>4. Combination Xpert+PPM resulted in a large total cost but also produced large additional benefit, due to the increase number of patient receiving effective TB and MDR-TB care.<br>5. Most scenarios in sensitivity analysis did not alter main result. Except when private provider intake was < 10%, which caused Xpert for all diagnosis strategy more cost effective than PPM and Xpert combination strategy.	1. Control group was the most costly option with the lowest QALY gain and highest mortality rate.<br>2. Single sputum Xpert produced higher QALY gain and lower mortality rate compare to smear plus Xpert.<br>3. Single sputum Xpert remain the most cost effective options when smear sensitivity ≤74%. Above the threshold, smear microscopy plus Xpert was more cost effective.	1. Due to the high influence of test volume towards cost per test, analysis is performed under three different test volume scenarios, i.e. low, observed, and high volume.<br>2. Under the low volume scenario, the ICER for both LED and Xpert exceeded the threshold.<br>3. Under high volume scenario, ICER dropped below threshold, and Xpert became more cost effective.<br>4. In sensitivity analysis, the main drivers of costs were TB test volume and TB prevalence.<br>5. LED was preferred in each test volume scenario when TB prevalence was low, and Xpert dominated when prevalence was high.<br>6. Eliminating ART cost (but maintaining benefit had limited impact (contrary to prior result).	1. Full roll-out of Xpert produced the greatest effect on at patient, health-system, and population level.<br>2. The diagnosis strategy option which targeted the use of Xpert on HIV positive population was dominated by same-day LED florescence and full roll-out of Xpert. 3. Novel diagnosis tools caused an increase in survival, which was followed by increase in cost. In 10 years analysis, there would be an increase of 25% to current TB budget and an increment in HIV budget of $ 8.6 million (for ART treatment).

Author / Information	**Kelly *et al.***[1]	**Little *et al.***[2]	**Suen *et al.***[3]	**You *et al.***[4]	**Zwerling *et al.***[5]	**Langley *et al.***[6]
Conclusion	LED fluorescence microscopy was cost effective for diagnosing sputum smear positive TB in adults in India (specifically in high workload medical college setting).	Active pulmonary TB diagnosis using IGRA in high burden setting, such as India, resulted in extensive overtreatment of non-infected individual as well as substantial incremental cost with little health gain.	Health system innovation is complementary to technological innovation. PPM program could provide benefit on its own. When combined with Xpert, it could add more benefit by increasing the access of TB patients in India to this new technology.	Employing a single sputum test with Xpert during initial assessment of hospitalized patients with presumptive active PTB appeared to be highly cost-effective in Hong Kong.	TB test volume and TB prevalence were the main cost drivers of TB diagnosis using Xpert and LED for newly diagnosed patients with HIV (presenting with at least one TB symptom). Xpert would be highly cost effective in high volume and TB prevalence settings.	It was predicted that full roll-out of Xpert was cost effective and could potentially reduce TB burden; however a substantial amount of fund would be required to translate it into clinical practice.
Study sponsored by manufacturer? (Yes/No)	No	No	No	No	No	No
Impact of diagnostic test included in the model	Diagnostic tool with higher sensitivity caused faster treatment initiation and better outcome (higher DALY averted).	Diagnostic tools with low specificity generated false positive cases which increased treatment cost. While those with higher sensitivity caused faster treatment initiation.	Diagnosis tools with higher sensitivity and faster turnaround time caused faster initiation with appropriate treatment and reduced source of infection.	In the sequential diagnosis strategy, the initial test with lower sensitivity cause high volume for subsequent test and add to the cost. Correct detection result in early treatment with the correct regimen (including MDR). Undiagnosed TB will be treated based on culture result but this is categorized as delayed treatment which caused higher mortality rate	Diagnostic tool with higher sensitivity caused faster treatment initiation; thus preventing fatality from untreated or wrongly treated TB (e.g. MDR TB treated with first line drug).	Diagnostic tool with higher sensitivity caused faster treatment initiation. Shorter turnaround time reduced transmission/infectious period and number of lost to follow up. False negative also contributed as source of transmission. False positive increased treatment cost. Higher survival due to faster detection and treatment for TB caused increased cost.
Impact of diagnostic test towards Health System included in the model (e.g. additional personnel, sending sample)	None	None	PPM increased access to a better TB care provided by public sector for TB patients who often sought care in private sector.	None	None	Rapid novel diagnosis tool caused reduction of health center visit, reduction in the number of sample to test as well as laboratory staff time.
Testing drop out included in the model? (Yes/No)	Not modeled directly, however the study assumed that only 72% of those with positive test result received treatment.	Yes (same rate of testing drop out for every diagnostic tools)	No	No	No	Yes
Diagnostic Care Setting	Demonstration Study Site (medical colleges)\	Public and private healthcare settings (IGRA is only performed in private lab)	Public and private provider	Hospital	Clinic and hospital where the demonstration study was conducted	Diagnostic districts in Tanzania (5 different diagnostic district with different characteristics, but all had high prevalence of HIV)
Adverse Event of Treatment Modeled (Yes/No) (**if treatment is consider as the impact of diagnosis)*	No	No	No	No	No	No
**Data Source**						
Pathogenic/epidemiologic	Data from the National TB Program	Published literature - epidemiological studies	Published literature (RNTCP performance report, observational study)	Published literature (clinical study of several diagnostic tools in Hong Kong, global epidemiology on TB drug resistance surveillance (Hong Kong region))	Published data (from demonstration study and site report)	Published literature (WHO report), Data from TB program (NTLP operational data and annual report), Data from Tanzania Ministry of health
Test Characteristic	Published literature (Systematic Review)	Published literature- Systematic review	Published literature (meta - analysis, demonstration/observational studies)	Published literature - systematic review, clinical diagnostic guideline validation study, prospective study	Published literature (policy statement, demonstration studies)	Published literature - Systematic review and Demonstration Trial
Effectiveness Measures (e.g. utility weight, etc.)	Published literature	Published literature - WHO report	Published literature	Published literature	Published Literature	Published literature - WHO report

Author<br>Information	**Kelly *et al.***[1]	**Little *et al.***[2]	**Suen *et al.***[3]	**You *et al.***[4]	**Zwerling *et al.***[5]	**Langley *et al.***[6]
Cost	Demonstration study and TB program data	Published literature-systematic review (cost for sputum smear), survey (IGRA), published price, WHO report	Published literature (report, global drug facility product catalogue, RNTCP document)	Hong Kong Gazette	Published Literature and demonstration study	Data from NTLP and Tanzania Ministry of Health, FIND negotiated price

S2 Table 2. Data Extraction for General Information (2)

Author / Information		**Schmid *et al.***[7]	**Choi *et al.***[8]	**Guerra *et al.***[9]	**Shah *et al.***[10]	**Sun *et al.***[11]	**van't Hoog *et al.***[12]
Type of PE Analysis		Cost-effectiveness Analysis	Cost Utility Analysis	Cost Effectiveness Analysis	Cost Utility Analysis	Cost Utility Analysis	Cost Utility Analysis
Publication Year		2014	2013	2013	2013	2013	2013
Country		Brazil	USA	Brazil	Uganda	South Africa and Uganda	India, Uganda, and South Africa
Objective		To evaluate, in an incarcerated population, the performance and accuracy, as well as cost effectiveness of several active pulmonary TB diagnosis algorithm comprised of smear, culture and Detect-TB	To evaluate the cost-effectiveness of incorporating Xpert into TB diagnostic algorithms	To estimate and compare the cost effectiveness of different strategies available in the primary health care unit in Rio de Janeiro City, Brazil	Determine the cost effectiveness of novel diagnosis algorithm combining sputum Xpert with urinary LF-LAM (lateral-flow lipoarabinomannan) for detecting active TB in people living with HIV	To determine the cost effectiveness of adding lateral-flow lipoarabinomannan (LF-LAM) to current active TB diagnosis strategy for highly immunocompromised adults	To inform the development of a Triage Test by exploring the combinations of sensitivity, specificity, and cost of a hypothetical triage test at which it could be more cost effective than Xpert for all presumptive TB cases
Type of Model (as stated in the study)		Decision Analytic Model	Decision Analytic Model	Decision Analytic Model	Decision Analytic Model	Decision Analytic Model	Decision Analytic Method
Perspective		Societal	Health system	Health system	Health system	Health system (public-sector TB program)	Health system (TB program)
Base/Reference Case		Smear microscopy	No molecular testing (conventional diagnostic: CXR, clinical evaluation, and smear microscopy)		Ziehl Neesen (ZN) smear microscopy	Smear microscopy, clinical judgement, and existing array of available additional diagnostic test	Xpert for all
Comparative Strategies	1	Culture	Selective MTD (conventional diagnostic and employing MTD to one sample if smear result was positive)	Smear microscopy following a Chest X-Ray result which was suggestive of TB	ZN smear microscopy and LF-LAM	LF-LAM added to the base case	Hypothetical 'triage' test for all, followed by Xpert, when 'triage' test result was positive
	2	Smear microscopy plus culture	Intensive MTD (conventional diagnostic and MTD, regardless smear result)	Smear microscopy followed by Chest X-Ray, when only 1 out of two sample was smear positive or 2 samples were smear negative	Xpert, followed by DST confirmation of rifampicin resistance	Substitution of smear microscopy with Xpert and addition of LF-LAM	
	3	Detect-TB (locally developed NAAT) alone	Selective Xpert (conventional diagnostic and employing Xpert to one sample if smear result was positive)	Chest X-Ray and smear microscopy was performed at the same visit.	Xpert and LF-LAM, followed by DST confirmation of rifampicin resistance		
	4	Smear microscopy plus Detect-TB	Intensive Xpert (conventional diagnostic and Xpert, regardless smear result)				

Author / Information		Schmid *et al.*[7]	Choi *et al.*[8]	Guerra *et al.*[9]	Shah *et al.*[10]	Sun *et al.*[11]	van't Hoog *et al.*[12]
Study population		Hypothetical cohort of 1000 patients, with HIV or not, admitted to a prison of Brazil.	Individuals with presumptive pulmonary TB disease in the United States	Patients attending a TB clinic at a primary health care unit in Rio de Janeiro City, adults (≥ 18 years old), with TB symptoms (cough more than 2 weeks or at least one other symptom presumptive of TB)	HIV infected individuals presenting with sign/symptoms of active TB disease in Uganda, including pulmonary, extra-pulmonary, and disseminated forms of TB (hypothetical cohort)	Hospitalized adults with known HIV infection CD4+ T cell count < 100 cells/mm^3 and clinical suspicion of TB	Cohort of 10.000 individuals with presumptive TB who require diagnostic testing
Type of Cost Incorporated	Direct	Diagnosis and treatment cost (Excluding HIV treatment cost), Cost incurred by patient (travel and food)	Diagnosis cost (staff, equipment and consumables) and treatment cost (including standard and MDR TB treatment cost, but not HIV treatment cost)	Diagnosis cost	Diagnosis cost (staff, equipment and consumables) and treatment cost (including standard and MDR TB treatment cost; in the base case HIV treatment cost was not included, but a secondary analysis which includes antiretroviral cost was conducted)	Diagnosis and treatment cost (Excluding HIV treatment cost)	Diagnosis and treatment cost (Excluding HIV treatment cost)
	Indirect	Income loss	Overhead Cost (5% and 10% of the total cost for each test system)		Overhead cost		Overhead cost (maintained from model source)
Health Outcomes Measures		Correctly diagnosed TB case	QALY accrued per patient	Patients with correct TB diagnosis, probability of correct TB diagnosis	DALY averted, TB case detected, and TB death averted	DALY averted, TB case treated, false positive treated	DALY averted *(using standard formulation, no information on age weighting)*, TB case detected
Discount Rate (per year)		3%	3%	Undiscounted	3%	3%	3%
Time horizon (as stated in the report)		Lifetime	Time frame: 1 year, analytical time horizon: life expectancy	N/A	1 year for estimation of costs and immediate effects; it was then extended to the life expectancy of cohort	cohort's lifetime	N/A
Analysis of Parameter uncertainty		Univariate (TB prevalence, accuracy, and variable cost)	Univariate, PSA	Univariate (limited to TB prevalence)	Univariate, PSA	Univariate, Multivariate (three-way sensitivity analysis), PSA	Univariate, PSA
Main Result		1. Smear alone strategy was both less expensive and less effective than other diagnosis options<br>2. Smear plus Detect-TB produced lower ICER compare to the other options	1. Implementing 'intensive Xpert' (full rollout) increased cost but also resulted in the most QALYs experienced per patient.<br>2. The 'no molecular testing' algorithm was dominated by all strategies incorporating MTD or Xpert.<br>3. Based on USA Willingness to Pay threshold, 'intensive Xpert' was highly cost effective compare to MTD selective Xpert.	1. The cost for strategies incorporating secondary diagnosis for negative result was higher than simultaneous smear microscopy and chest X-ray strategy.<br>2. Strategies with secondary diagnostic test required three visits, hence there were additional cost for multiple consultations.<br>3. Lowest probability of correct TB diagnosis was obtained from the smear microscopy following suggestive Chest X-ray strategy.	1. The study found increasing diagnostic options consequences (number of TB case detected and cost) in the following order: smear, smear + LF LAM, Xpert, Xpert + LF - LAM.<br>2. Based on the per capita GDP threshold, Xpert plus LF-LAM was considered highly cost effective compare to Xpert alone.<br>3. Life expectancy following TB treatment, as well as cost and effect for HIV/ART treatment influenced Xpert ICER.	1. Addition of urine LAM resulted in 80 additional true-positive and 25 additional false-positive.<br>2. The probability of acceptability at willingness to pay equal to GDP, was > 99.8% in both countries.<br>3. ICER was sensitive to life expectancy after TB cure.	1. When Triage sensitivity was 100% relative to Xpert, a 60% reduction of cost can be achieved if specificity was 85% and cost per patient was $2.<br>2. Lower sensitivity of triage test yielded ICER which favored Xpert strategy without triage test.<br>3. In settings with a lower willingness to pay threshold, several specificity and price combinations of a triage test with lower sensitivity would be attractive.

Author<br>**Information**	**Schmid *et al.***[7]	**Choi *et al.***[8]	**Guerra *et al.***[9]	**Shah *et al.***[10]	**Sun *et al.***[11]	**van't Hoog *et al.***[12]
Conclusion	Despite report showing the high cost of PCR, Detect-TB proved to be the most cost-effective option for TB diagnosis when used in combination with smear microscopy.	Implementation of Xpert for the diagnosis of PTB in The United States was cost-effective.	Sputum smear and Chest X-ray for all PTB suspect was the most cost effective option. Although disparity of cost effectiveness between options was small, the strategy showed benefit by reducing the number of visit.	The addition of LF-LAM to the TB diagnosis algorithm for HIV patients (presenting with TB symptoms) was highly cost-effective compare to Xpert or Smear alone.	Urine LAM, a true point-of-care testing, was likely to be both effective and cost effective when used to diagnose TB for severely immunocompromised, hospitalized African adults in high burden setting (lower and upper-middle income country)	Employing a triage test to select persons for confirmatory testing with Xpert could substantially improve the affordability of Xpert for TB diagnosis, particularly a triage test with high sensitivity and modest specificity, which yielded high case finding.
Study sponsored by manufacturer? (Yes/No)	No	No	No	No	No	No
Impact of diagnostic test included in the model	Diagnostic tool with higher accuracy yielded higher number of correctly diagnosed TB.	Diagnostic tool with higher sensitivity caused faster treatment initiation as well as shorter length of hospitalization and isolation. False negative experienced higher mortality due to delayed treatment. False positive cases increased treatment cost, due to unnecessary hospitalization, isolation, and treatment.	Correct determination of active TB status (treatment was not included in the model)	Diagnostic tool with higher sensitivity caused faster treatment initiation. False positive increased treatment cost. Availability of diagnostic tools with better accuracy reduced clinical diagnosis and empiric treatment.	Diagnostic tool with higher sensitivity caused faster treatment initiation. Untreated false negative cases would experience high mortality rate. False positive cases increased treatment cost.	Triage test with high sensitivity reduced the number of specimen required to be tested by Xpert, and caused cost reduction.
Impact of diagnostic test towards Health System included in the model (e.g. additional personnel, sending sample)	None	None	None	None	None	None
Testing drop out included in the model? (Yes/No)	No	No	Loss to follow up is recorded in the parent study but no consequences was included in the CE model	No	No	No
Diagnostic Care Setting	Inpatient and outpatient setting for incarcerated population.	Not detailed	Primary healthcare unit	Based on parent study: hospital and Infection Disease Institute	Inpatients	Not detailed
Adverse Event of Treatment Modeled (Yes/No) (**if treatment is consider as the impact of diagnosis)*	N/A	Yes (disutility due to treatment hepatotoxicity)	N/A	No	Yes (death from TB treatment toxicities)	No
**Data Source**						
Pathogenic/epidemiologic	Published literature (epidemiology study in the setting), Demonstration Trial	Published study, CDC report	Parent study data, local prevalence study	Trial (parent study)	Trial Data; Published literature (mortality)	WHO report, assumption (smear positive TB prevalence), demonstration trial
Test Characteristic	Demonstration trial	Assumption, Published Literature	Parent study data	Trial (parent study)	Trial Data; Published literature	Demonstration trial, hypothetical (Triage test)
Effectiveness Measures (e.g. utility weight, etc.)	Demonstration trial	Published literature	Parent study data	Published literature - WHO report	Published Literature - WHO report	Published literature - WHO report

Author<br>Information	**Schmid *et al.***[7]	**Choi *et al.***[8]	**Guerra *et al.***[9]	**Shah *et al.***[10]	**Sun *et al.***[11]	**van't Hoog *et al.***[12]
Cost	Demonstration trial, Published literature (duration of stay in hospital and number of test and consultation, etc.)	Direct observation, local health department invoice and budget records, published literature, manufacturer quotations, published estimates (wage and salary)	Parent study data, SIGTAP-SUS	Trial (parent study), published literature	Published Literature	Demonstration trial, published literature

S2 Table 3. Data Extraction for General Information (3)

Author<br>Information	**Abimbola *et al.***[13]	**Menzies *et al.***[14]	**Dowdy *et al.***[15]	**Hughes *et al.***[16]	**Vassall *et al.***[17]	**Chihota *et al.***[18]
Type of PE Analysis	Cost Effectiveness Analysis	Cost Utility Analysis	Cost Utility Analysis (main outcome: cost/DALY averted)	Cost Utility Analysis	Cost Utility Analysis	Cost Effectiveness Analysis
Publication Year	2012	2012	2011	2011	2011	2010
Country	Sub Saharan African	Botswana, Lesotho, Namibia, South Africa, and Swaziland	India	UK	India, Uganda, and South Africa	South Africa
Objective	To evaluate the cost effectiveness of improved diagnosis strategy for TB early detection, as well as death reduction in advance HIV patients, presenting for ART initiation	To quantify the potential health and economic consequences of introducing Xpert in five Southern African countries characterized by high prevalence of HIV infection and extant multidrug resistance	To better understand the economic and epidemiological consequences of serological testing for active TB in India	Evaluate the cost effectiveness of currently available diagnostic strategies for routine diagnosis of TB in the NHS, to inform resource allocation policy	To assess whether Xpert resulted in improvement of the cost effectiveness of TB care in low and middle income settings	To conduct cost effectiveness analysis of TB smear negative diagnosis using several culture method, i.e. MGIT, MGIT+LJ versus LJ alone, as well as various species identification method (biochemical assays, cording and anti-MPB64 assay)
Type of Model (as stated in the study)	Decision Analytic Model	Dynamic Transmission Model	Decision Analytic Model	Decision Tree Model	Decision Analytic Model	Decision Tree Model
Perspective	Health system	Health system	Health system (private and public TB-control sector)	Health System	Health service	Health service providers
Base/Reference Case	Smear microscopy followed by chest X-Ray when smear result was negative	Status Quo Scenario:<br>-. initial sputum smear microscopy<br>-. sputum culture for smear negative cases, if previously had been treated for TB or when clinical suspicion was strong<br>-. DST for treatment experienced patient who was tested positive for TB	**First Stage analysis:** combination of tests and clinical strategies, without microbiological test<br>**Second Stage analysis:** sputum smear microscopy as initial test		Sputum smear microscopy, followed by clinical diagnosis for smear negative cases (might include chest X-ray and empirical antibiotic treatment)	LJ culture, followed by species identification using biochemical assay, anti - MPB64, or cording

Information		Abimbola *et al.*[13]	Menzies *et al.*[14]	Dowdy *et al.*[15]	Hughes *et al.*[16]	Vassall *et al.*[17]	Chihota *et al.*[18]
Comparative Strategies	1	Mycobacterial culture testing for undiagnosed patient based on smear microscopy and Chest X-ray	Xpert as initial test:<br>-. When result is positive, but rifampicin resistance was negative, patient received first line treatment<br>-. Rifampicin resistance was confirmed with DST. Treatment with second line drugs was based on DST result.	**First stage analysis 1:**<br>Smear microscopy added to base case<br>**Second stage analysis 1:**<br>Serology testing was added for smear negative cases	Smear microscopy, followed by culture testing when smear was positive	Sputum smear microscopy, followed by Xpert for smear negative case	MGIT culture followed by species identification using, biochemical assay, anti -MPB64, or cording
	2	Xpert as the only test		**First stage analysis 2:**<br>Serology testing added to base case<br>**Second stage analysis 2:**<br>Automatic Liquid Culture was added for smear negative cases	Smear microscopy, followed by culture testing when smear was negative	Single sputum Xpert for all, as initial test	MGIT and LJ culture, followed by species identification using biochemical assay, anti -MPB64, or cording
	3				Smear microscopy, followed by culture testing every time		
	4				Smear microscopy and NAAT, culture was conducted when discrepancy between results exist		
	5				Smear microscopy, followed by NAAT for smear positive, otherwise culture		
	6				Smear microscopy, followed by NAAT for smear negative, otherwise culture		
	7				NAAT followed by culture in every situation		
	8				NAAT only		
	9				NAAT, followed by culture when NAAT positive		
	10				NAAT, followed by culture when NAAT negative		
	11				Sputum Smear microscopy and NAAT followed by Culture every time		
Study population		A group of patient (HIV +) eligible for ART based on the presence of clinical illness and/or CD4 cell count of < 200 cell/microliter. Patient has TB symptoms such as cough, fever, weight loss, or night sweats	Adult population, HIV positive or negative with various CD4+ count status, with prior or no experience of TB treatment	Hypothetical cohort of 1.5 million adult with presumptive TB in India presenting for diagnosis (have access to serological test)	Population was not mentioned explicitly, but could be implied from the model that population was adult with presumptive TB in UK	Hypothetical cohort of 10.000 individuals suspected of having TB	TB suspect, enrolled from routine mine health service and from Thibela TB study (median age 43 years)
Type of Cost Incorporated	Direct	Diagnosis and treatment cost (Including HIV treatment cost)	Diagnosis and treatment cost (standard and MDR TB, as well as HIV treatment cost)	Diagnosis and treatment cost (Excluding HIV treatment cost)	Diagnosis and treatment cost (including second line TB treatment and follow up management)	Diagnosis and treatment cost (including diagnostic antibiotic empirical treatment, standard and MDR TB treatment; excluding HIV treatment cost)	Diagnosis cost (excluding cost for specimen collection, transport to laboratory and return of results)
	Indirect		Overhead cost (managerial overhead cost)	Overhead cost		Overhead cost	Overhead cost (e.g. maintenance, cleaning, administrative staff)

Author<br>Information	Abimbola *et al.*[13]	Menzies *et al.*[14]	Dowdy *et al.*[15]	Hughes *et al.*[16]	Vassall *et al.*[17]	Chihota *et al.*[18]
Health Outcomes Measures	Death averted	DALY averted *(calculation method is undisclosed*), life-years saved, population-level epidemiological outcomes (prevalence, incidence, mortality, MDR TB prevalence, annual infection risk)	DALY averted (*calculation method is undisclosed*), secondary TB cases, additional TB cases treated, additional false positive treated	QALY, secondary infection	DALY averted (*using standard formulation, no information on age weighting*), total TB cases detected, total MDR TB cases detected	*M. tuberculosis* case identified
Discount Rate (per year)	Undiscounted	3%	3%	Undiscounted (short term analysis, i.e. the first year following presenting for diagnosis)	3%	3%
Time horizon (as stated in the report)	First 6 months following initiation of ART	10 and 20 years	Costs and effects of TB treatment occur during the first year after presentation. The outcomes which occurred that year were discounted over the cohort's lifetime (analytical time horizon)	1 year	N/A	N/A
Analysis of Parameter uncertainty	Univariate, PSA	Univariate, PSA	Univariate and Multivariate (Two & Three was sensitivity analysis)	Univariate and PSA	Univariate, Multivariate (Two way), and PSA	Univariate (MGIT throughput, overhead cost, contamination rate of MGIT)
Main Result	1. Compare to the current practice, diagnosis with culture resulted in higher healthcare utilization costs, while Xpert resulted in lower costs.<br>2. Although culture was the most effective diagnosis in averting early deaths (detect 86 cases, while Xpert detect 78 cases), it was not the strategy with the lowest cost.	1. The positive predictive value for RIF resistance by Xpert in the study setting was low; however number of false positive was countered by Drug Sensitivity Testing confirmation.<br>2. 10 years after the introduction of Xpert prevalence would be lower by 186 per 100.000 population.<br>3. The absolute number of MDR cases would also be lower by 25%.<br>4. Implication of Xpert introduction was extended to the cost for HIV treatment (higher survival caused higher demand for ART treatment).	1. Adding sputum smear to the base case would diagnose 44% of the undetected cases.<br>2. Serology detected more TB cases with the expense of increased number of false positive<br>3. Smear detected cases were regarded as more infectious therefore it averted more DALY and secondary cases compared to serology.<br>4. When conducted only to smear negative, culture was more cost effective than serology.<br>5. In sensitivity analysis serology testing was not cost effective compared to smear microscopy in all scenarios.	1. Cost difference was mainly driven by cost of test.<br>2. At UK threshold, strategy incorporating sputum smear microscopy followed by culture was the most cost effective option<br>3. NAAT use alongside with SSM could be cost effective when prevalence of TB during pre-test was above 46%.	1. Xpert use was associated with increased case findings as well as cost.<br>2. All of the ICER for strategies incorporating Xpert were below willingness to pay. 3. Increased proportion of TB and MDR TB would affect Xpert cost effectiveness negatively (higher treatment cost).<br>4. Counterintuitive result: cost effectiveness of Xpert did not improve with increase HIV prevalence (relationship between HIV and Xpert was weaker than expected)	1. MGIT yield for mycobacteria was higher than LJ. 2. MGIT identified 151 and 225 culture positive for smear positive and smear negative respectively; while LJ identified positive culture of 144 and 145, for smear positive and smear negative respectively.<br>3. Among culture-positive identified by MGIT, only 22,7% was identified as *M. tuberculosis;* the majority was identified as NTM (Non Tuberculosis Mycobacterium)
Conclusion	Diagnosis with culture and Xpert were cost effective at reducing early mortality during the first 6 months of ART compared with the current practice.	Introduction of Xpert changed TB morbidity and mortality (by improving case finding and treatment), and created limited impact towards long term transmission dynamic. Adoption of Xpert was reasonable, based on conventional threshold; however, the additional financial burden would be substantial.	As an initial test, serology increased per patient cost, generated more DALY and false positive cases. In area where high quality sputum smear was available, adding automated liquid culture (MGIT) was more effective and least costly compared to serology	At low tuberculosis prevalence, NAAT may not be cost effective; however it showed potential when employed in setting with higher prevalence.	Xpert was cost effective for TB diagnosis compare to a base case (smear microscopy and clinical diagnosis for smear negative) in low middle income countries, due to its' ability to increase case finding.	MGIT produced higher yield and faster result at a relatively higher cost. The cost effectiveness was sensitive to NTM contamination. Both cording and anti-MPB64, were effectively comparable to standard biochemical assay for species identification at a lower cost.
Study sponsored by manufacturer? (Yes/No)	No	No	No	No	No	No
**Modeling Details**						

Author / Information	**Abimbola *et al.***[13]	**Menzies *et al.***[14]	**Dowdy *et al.***[15]	**Hughes *et al.***[16]	**Vassall *et al.***[17]	**Chihota *et al.***[18]
Impact of diagnostic test included in the model	Diagnostic tool with higher sensitivity caused faster treatment initiation, thus preventing fatality from untreated TB.	Diagnostic tool with higher sensitivity caused less false negative cases and shorter time to initiate correct treatment. Shorter turnaround time of diagnosis tool reduced transmission/infectious period and number of lost to follow up. Less numbers of false negative also contributed to the reduced transmission. False positive increased treatment cost. Higher survival due to faster detection and treatment caused increased in cost for Anti-retroviral therapy in HIV positive patients.	Diagnosis strategy with low specificity generated substantial number of false positive cases which increased treatment cost. Diagnostic tool with higher sensitivity resulted in faster treatment initiation. Numbers of secondary cases were predicted from undiagnosed patient (false negative) and loss to follow up.	Novel diagnosis tools caused faster initiation of correct treatment for true positive (including MDR treatment), but could add unnecessary treatment for false positive (depending on specificity). Secondary infection happened due to delay treatment in false negative patient.	Diagnostic tool with higher sensitivity caused faster correct treatment initiation. Numbers of false positive cases increased treatment cost.	Culture with better sensitivity could identify more mycobacterium but not necessarily exclude NTM. Species identification method confirmed culture result. No impact on treatment was modeled
Impact of diagnostic test towards Health System included in the model (e.g. additional personnel, sending sample)	None	None	None	None	None	None
Testing drop out included in the model? (Yes/No)	No	Yes	Yes (longer turnaround time create larger loss to follow up in diagnosis)	No	No	No
Diagnostic Care Setting	Not detailed	Not detailed	Public and private healthcare settings	Outpatient	Not detailed	Mine health service
Adverse Event of Treatment Modeled (Yes/No) (**if treatment is consider as the impact of diagnosis)*	No	No	No	Yes (toxicity due to anti TB)	No	N/A
**Data Source**						
Pathogenic/epidemiologic	Published literature	WHO unpublished data, United Nations Population Division for projection	Published literature - epidemiological study	Expert Opinion (pre-test prevalence)	WHO report, model assumption, demonstration study	Demonstration study
Test Characteristic	Published literature	Published literature	Published literature - Meta analysis, WHO report	Published Literature (Systematic Review)	Published literature - Demonstration study, model assumption	Demonstration study
Effectiveness Measures (e.g. utility weight, etc.)	Published literature data)	Published Literature	Published literature - WHO report	Published literature	Published literature - Epidemiological studies, WHO report	Demonstration study
Cost	Published literature (South Africa cost which is considered the high end of cost that would incur in the sub-Saharan Africa region)	Published literature (WHO price reporting mechanism, WHO-CHOICE project)	Published literature - WHO training material, economic evaluation study	Published literature (including from Department of Health)	**Diagnostic cost**: Demonstration trial (Observation of practice, financial report, interview) **Treatment cost**: WHO, published literature	Demonstration study

S2 Table 4. Data Extraction for General Information (4)

Information		Bonnet *et al.*[19]	Scherer *et al*[20]	Dowdy *et al.* (1)[21]	Dowdy *et al.* (2)[22]	Guerra *et al.*[23]
Type of PE Analysis		Cost Effectiveness Analysis	Cost Effectiveness Analysis	Cost Utility Analysis (main outcome: cost/DALY averted)	Cost Utility Analysis (main outcome: cost/DALY averted)	Cost Effectiveness Analysis
Publication Year		2010	2009	2008	2008	2008
Country		Kenya	Brazil	Brazil	South Africa, Brazil, Kenya	USA
Objective		To compare the relative cost effectiveness of different microscopy approaches, combining direct smear and smear after overnight NaOCl sedimentation, in Kenya.	To investigate the cost-effectiveness of a home-made colorimetric PCR to diagnose TB, in parallel with the use of ZN smear microscopy	To estimate the impact and cost-effectiveness of mycobacterial culture for the diagnosis of TB in an urban setting in Latin America	To analyze the potential cost-effectiveness of adding a hypothetical new point-of-care test to the current TB diagnostic approach in three high burden countries	To compare the cost effectiveness of different specimen dilution algorithms for MTD testing during evaluation of PTB suspect
Type of Model (as stated in the study)		Decision Tree Model	Decision Analytic Model	Decision Analytic Model, Markov Process (to model re-diagnosis of false negative)	Decision Analytic Model	Decision Analytic Model
Perspective		Health service provider	Not clearly stated	Health system (public-sector TB control program)	Health System (hypothetical TB program in The Ministry of Health)	Health service providers (laboratory)
Base/Reference Case				Sputum smear microscopy in local laboratory (without sputum culture)	Combination of test and clinical strategies, without any microbiology test	
Comparative Strategies	1	D1 + D2 : direct smear on the first specimen, followed by seconds specimen testing when the first one was negative	Smear microscopy used with culture	Sputum smear and culture testing with Solid LJ media	Hypothetical new 'point-of-care' testing	"CDC strategy" : conventional MTD testing was performed to all specimen; negative result was retested with internal amplification control
	2	B1 : bleach smear microscopy on first specimen	Smear microscopy used with in-house PCR (PCR colorimetric dot-blot assay)	Sputum smear and culture testing with Liquid MGIT media	Sputum smear microscopy	"Simultaneous strategy" : both conventional and dilution technique for MTD testing was performed to all specimen simultaneously
	3	B1 + B2 : bleach smear microscopy on first specimen, followed on bleach for the second specimen if the first one was negative			Sputum smear microscopy plus hypothetical new 'point-of-care' testing	"Smear positive dilution strategy" : dilution technique for MTD testing was performed to all smear-positive specimen, while conventional technique was employed to smear negative
	4	D1 + B1 : direct smear microscopy on the first specimen, bleach smear microscopy on the first specimen when the first one was negative			Sputum smear microscopy plus sputum culture	"Sequential dilution strategy": conventional MTD testing was performed to all specimen, followed by dilution technique of MTD testing for negative or unequivocal result
	5	B1 + D2 : Bleach smear microscopy on the first specimen, direct smear on the second specimen when the first one was negative				

Author<br>Information		Bonnet *et al.*[19]	Scherer *et al*[20]	Dowdy *et al.* (1)[21]	Dowdy *et al.* (2)[22]	Guerra *et al.*[23]
	6	D1 + B2 : Direct smear microscopy on first specimen, bleach smear microscopy on the second if the first one was negative				
	7	D1 + B1 + D2 : Direct smear microscopy on first specimen; bleach microscopy on first and direct smear on second specimen if the first smear was negative				
	8	D1 + D2 + B2 : Direct smear microscopy on first specimen, direct smear on the second if the first one was negative, bleach microscopy on the second if result was still negative				
	9	D1 + B1 + B2 : Direct smear microscopy on the first specimen, bleach microscopy on the first if the first one was negative, and bleach microscopy on the second if the result was still negative				
	10	B1 + D2 + B2 : Bleach smear microscopy on first specimen and direct smear microscopy on second. Bleach on second when 2 previous smears were negative				
Study population		TB suspects presenting with cough (≥ 2 weeks) in an urban clinic in Nairobi, Kenya	Adults with presumptive PTB (reporting cough of more than 3 weeks) referred to TB and HIV Reference Center	Hypothetical cohort of HIV-positive patients presenting to municipal health clinics with symptoms of pulmonary TB (e.g. three weeks of cough)	Adults with presumptive TB initiated on TB diagnostic workup	Individuals, from whom sputum sample was collected and tested by Maryland Department of Health and Mental Hygiene
Type of Cost Incorporated	Direct	Diagnosis cost (limited to smear microscopy; additional diagnosis to detect smear negative or non TB case is not included), patient transport cost (during demonstration study, certain amount of patient's transport cost was covered by the clinic)	Diagnosis and treatment cost (the report did not mention HIV treatment), cost incurred by patient (travel, and food)	Diagnosis and treatment cost (Excluding HIV treatment cost)	Diagnosis and treatment cost (Excluding HIV treatment cost, as well as physician visit and hospitalization)	Diagnosis cost
	Indirect		Income loss			
Health Outcomes Measures		Smear positive detection rate	Total number of accurately diagnosed and treated, total number of incorrectly diagnosed and incorrectly untreated or treated (false negative and false positive)	DALY averted , incremental TB diagnosed, deaths averted, secondary infections (calculated from undiagnosed and untreated cases)	DALY averted, TB infections prevented, secondary TB infection averted	Correct pulmonary TB diagnosis
Discount Rate (per year)		Undiscounted	Undiscounted	3%	3%	Undiscounted

Author<br>Information	Bonnet *et al.*[19]	Scherer *et al*[20]	Dowdy *et al.* (1)[21]	Dowdy *et al.* (2)[22]	Guerra *et al.*[23]
Time horizon (as stated in the report)	N/A	N/A	Lifetime of the cohort	1 Year from presentation	N/A
Analysis of Parameter uncertainty	Univariate (monthly salary of lab technician, patient transport cost), multivariate (using data set from another settings)	Univariate (TB prevalence, sensitivity, specificity, and variable costs)	Univariate and PSA	Univariate, multivariate (three-way sensitivity analysis)	Univariate
Main Result	1. All strategies which incorporated bleached smear significantly detected more cases than base case strategy.<br>2. Strategy B1+B2 dominated other strategies. \<br>3. Doubling the transport cost, lower the cost effectiveness of strategies which required multiple visit.	1. Total cost for strategy incorporating PCR was 3.8 times lower than smear microscopy plus culture. 2. The lower cost was mainly due to lower income lost (due to faster turnaround time of PCR), and treatment cost (shorter hospital stay for PCR strategy).<br>3. Overall cost per accurately diagnosed cases of TB was lower for PCR.	1. Implementing solid culture would avert 8 TB deaths and 17 secondary infections but would trigger false treatment in additional 44 patients.<br>2. Replacing solid media with MGIT would avert one further death but would generate 35 further inappropriate treatments.<br>3. ICER of MGIT was higher than solid media (due to lower incremental DALY of MGIT compare to incremental DALY of solid media compare to base case)<br>4. Higher number of patients detected per week (higher throughput) lead to a better ICER profile due to lower cost.	1. As initial test, sputum smear was more cost-effective than a new test with 70% sensitivity, 95% specificity, and price of $ 20 in all three countries.<br>2. Adding a new test to sputum microscopy increased the yield of DALY averted by sputum smear alone, and also averted more secondary infection.<br>3. In all scenarios, adding new test to sputum was more costly but also more effective that adding TB culture.	1. The 'smear positive dilution' strategy yielded the lowest cost per correct diagnosis<br>2. The strategy remained cost effective compared reference case, even when the proportion of smear positive and prevalence of TB in smear positive were very low (0.1)
Conclusion	Sputum smear microscopy alternatives showed limited increase in laboratory's workload and could potentially be implemented in TB diagnosis program.	Strategy incorporating sputum smear plus PCR dot-blot to diagnose TB was potentially cost effective especially in a hospital setting of developing countries with high TB burden. The strategy increased sensitivity, negative predictive value, and improved ability to rule out pulmonary TB.	TB culture for HIV patients in Urban Brazil was potentially effective and cost-effective. It needed to be supported by strong communications between lab, clinic, and patients.	Novel diagnostic test for Pulmonary TB was potentially cost-effective. Cost effectiveness depended most strongly on specificity and price of new test, as well as discount rate. It was also maximized in areas currently equipped with weak diagnosis infrastructures.	In the laboratory setting, an MTD testing strategy which incorporated the dilution specifically to smear positive sample was more cost-effective than the other strategies.
Study sponsored by manufacturer? (Yes/No)	No	No	No	Yes	No
**Modeling Details**					
Impact of diagnostic test included in the model	Since no gold standard was used, only an increase in smear positive yield can be measured without determining possibility of false positive case	Diagnostic tool with higher sensitivity caused faster treatment initiation. False positive increased treatment cost.	Diagnostic tool with higher sensitivity caused faster treatment initiation. False positive increased treatment cost.	Diagnostic tool with higher sensitivity caused faster treatment initiation. Shorter turnaround time reduced loss to follow up	Generate correct diagnosis (no treatment is modeled)
Impact of diagnostic test towards Health System included in the model (e.g. additional personnel, sending sample)	None	None	None	No	None
Testing drop out included in the model? (Yes/No)	No	No	No	Yes	No
Diagnostic Care Setting	Urban health Clinic	Hospital	Reference case: local laboratories; Culture: central laboratory (municipal)	Lab service dedicated to TB	Reference Laboratory
Adverse Event of Treatment Modeled (Yes/No) (**if treatment is consider as the impact of diagnosis)*	N/A	No	No	No	N/A

Author<br>Information	**Bonnet *et al.***[19]	**Scherer *et al***[20]	**Dowdy *et al.* (1)**[21]	**Dowdy *et al.* (2)**[22]	**Guerra *et al.***[23]
**Data Source**					
Pathogenic/epidemiologic	Demonstration study	Trial Data (parent study) (not specified in the report)	Trial Data	Published literature - epidemiological study	Retrospective review of data from laboratory
Test Characteristic	Demonstration study	Trial Data (parent study)	Trial Data and Published Literature	Published literature- modelling study, meta-analysis, demonstration study in the study setting, demonstration study from other settings	Retrospective review of data from laboratory
Effectiveness Measures (e.g. utility weight, etc.)	Demonstration study	Model generated (number of TB diagnosed, etc.)	Published Literature	Published literature - WHO report	Retrospective review of data from laboratory
Cost	Demonstration study	Trial Data (parent study)	Trial Data (lab budget and staff interview), published literature	National TB program data	Retrospective review of data from laboratory

S2 Table 5. Data Extraction of General Information (5)

Information		Author: **Mueller *et al.***[24]	**Rajalahti *et al.***[25]	**Dowdy *et al.***[26]	**Roos *et al.***[27]
Type of PE Analysis		Cost Effectiveness Analysis	Cost Effectiveness Analysis	Cost Effectiveness Analysis	Cost Effectiveness Analysis
Publication Year		2008	2004	2003	1998
Country		Zambia	Finland	USA	Kenya
Objective		To examine the cost and cost effectiveness of homemade and commercially produced Löwenstein-Jensen culture (HLJ and CLJ) as well as automated and manually read liquid culture (AMGIT and MMGIT) in resource poor setting (Zambian National TB Reference Laboratory)	To perform an economic evaluation of two different TB diagnosis strategies, i.e. PCR and No PCR, in a low burden settings	To determine whether the cost for routine implementation of Amplified Mycobacterium Tuberculosis Direct test (MTD) in clinical settings was offset by savings on isolation room and medication expense	To assess the potential cost-effectiveness of PCR for the diagnosis of TB in an urban setting of a developing country
Type of Model (as stated in the study)		Not mentioned (method implied the use of Decision Analytic Model)	Decision Tree Model	Decision Analytic Model	Not mentioned (method implied the use of Decision Analytic Model)
Perspective		Health service providers	Not clearly stated, inputs indicated health service provider perspective	Health service providers or health care system	Not clearly stated, inputs indicated societal perspective
Base/Reference Case			Currently applied diagnosis: No PCR strategy (diagnosis based on sputum smear microscopy and culture)	Smear positive patient received confirmation testing using sputum culture	Routine diagnostic procedure: Sputum smear microscopy performed to 3 sputum specimens collected on different times, followed by Chest X-ray (sometimes empirical antibiotic or referred to other clinic) when negative result was obtained
Comparative Strategies	1	Sputum Culture Using Home-made LJ Media (HJL)	PCR strategy: addition of PCR to smear and culture testing	Smear positive patient received confirmation testing using Amplified Mycobacterium Tuberculosis Direct test (MTD)	Anticipated PCR strategy: PCR testing was conducted to a single sputum sample, no follow up for negative result
	2	Sputum Culture Using Commercial LJ Media (CJL)			
	3	Sputum Culture Using Manual Mycobacteria Growth Indicator Tube (MMGIT)			
	4	Sputum Culture Using Automatic Mycobacteria Growth Indicator Tube (AMGIT)			
Study population		Population was not mentioned explicitly, but could be implied from the model that population was adult TB suspect from whom sample was collected and tested by Zambian National TB Reference Laboratory	Patients with presumptive TB referred for sputum smear examination from primary care or hospital	Hospitalized person with symptoms consistent with active TB and AFB smear positive in at least one of the sputum specimen	TB suspect presenting in major out-patient TB clinic in Nairobi, Kenya
Type of Cost Incorporated	Direct	Diagnosis cost	Diagnosis and treatment cost (including isolation, hospitalization, TB treatment, as well as antibiotic treatment for suspected respiratory infection (levofloxacin))	Diagnosis and treatment cost (including isolation room)	Diagnostic and treatment cost (including antibiotic treatment for smear negative patients in base case, standard TB, and false positive treatment), cost incur by patients (travel, examinations, and other expenditures)
	Indirect	Overhead cost (maintenance, capital cost, utilities, management, cleaning etc.)			Income loss
Health Outcomes Measures		Identified *M. tuberculosis* specimen	Probability of correct decision to treat and to isolate TB suspect	Early TB exclusion	Correctly diagnosed TB

Author<br>Information	**Mueller *et al.*[24]**	**Rajalahti *et al.*[25]**	**Dowdy *et al.*[26]**	**Roos *et al.*[27]**
Discount Rate (per year)	Undiscounted	Undiscounted	5% (discount is employed to calculate annual cost for isolation room)	Undiscounted
Time horizon (as stated in the report)	N/A	N/A	N/A	N/A
Analysis of Parameter uncertainty	Univariate (difference in two culture methods yield)	Univariate and Bivariate	Univariate (all parameters, except for cost) and Multivariate (Three-way sensitivity analysis)	Univariate
Main Result	1. Cost per identified *M. tuberculosis* varied greatly for the four methods due to the different *M. tuberculosis yield*.<br>2. Demonstration study showed that the *M. tuberculosis* yield for solid media and liquid media were 9% and 16% respectively.<br>3. Higher testing throughput resulted in lower cost for all method 4.lowering the yield of liquid media culture cause higher cost	1. Under base case scenario (PCR available in 4 days, and culture results was obtained in 2 weeks) the cost/patient for No PCR strategy was 12 % lower.<br>2. When PCR testing was only employed to smear positive sputum specimen, the strategy dominated No PCR strategy, due to higher effectiveness (higher probability of correct decision to treat and isolate patients).<br>3. When PCR is performed daily and the culture time is 3 weeks, PCR strategy would cost less compared to No PCR strategy.	1. In a base case, a routine MTD testing program costed $494 per early exclusion of TV/early exclusion of TB.<br>2. The cost was not offset by the saving from averted isolation which was $210.<br>3. Sensitivity analysis showed that the result was influenced by prevalence of TB in smear positive population.	1. In smear microscopy strategy, the cost incurred by patient was almost 33% of the total screening budget, while in PCR strategy, it was only accounted for 12%. This was due to multiple sampling needed in the smear strategy. The number of missed diagnosis was higher for smear strategy. 2. PCR strategy resulted in higher total cost and number of correctly diagnosed TB cases.<br>3. Lower burden/lower prevalence, drove the cost effectiveness ratio for PCR higher (due to the small number of true positive generated)
Conclusion	Both MGIT methods were more superior compare to LJ in term of cost-effectiveness due to the higher yield of MGIT. Regardless the technique, culture method was associated with high cost which could limit the expansion of culture for TB diagnosis. Furthermore, the use of culture in decentralized setting with low testing throughput was related to even higher costs.	Routine PCR testing of all specimens from suspected TB patients in a low-prevalence population was not cost-saving. PCR strategy dominated base-case strategy when it was applied specifically to smear-positive sputum specimen.	Routine MTD testing of smear-positive specimen was not cost saving for most individual hospitalized in a low burden setting. Centralized reference laboratories may be able to implement MTD in a cost-effective manner across a wide range of situations.	The PCR method for TB detection was potentially cost effective compared to routine diagnosis. However, further study was needed to investigate major components which could influence cost effectiveness.
Study sponsored by manufacturer? (Yes/No)	No	No	No	No
**Modeling Details**				
Impact of diagnostic test included in the model	Generate correct diagnosis (no treatment is modeled)	Correct decision to treat and to isolate	Early confirmation of Non TB mycobacterium infection could avert isolation and unnecessary medication.	Generate correct diagnosis (no treatment is modeled)
Impact of diagnostic test towards Health System included in the model (e.g. additional personnel, sending sample)	None	None	None	None
Testing drop out included in the model? (Yes/No)	No	No	No	No
Diagnostic Care Setting	Reference Laboratory	Inpatient & Outpatient	Inpatient	Outpatient clinic
Adverse Event of Treatment Modeled (Yes/No) (**if treatment is consider as the impact of diagnosis)*	N/A	N/A	N/A	N/A
**Data Source**				
Pathogenic/epidemiologic	Not detailed	Hospital record, Retrospective patient data	observational data from setting's clinical microbiology laboratory	Operational data from the clinic
Test Characteristic	Parent study	Published literature (demonstration study)	Published studies (Medline literature review)	Assumptions
Effectiveness Measures (e.g. utility weight, etc.)	Parent study (correct diagnosis)	Published literature (demonstration study)	Published studies (Medline literature review)	Calculation and assumptions

Author / Information	**Mueller *et al.***[24]	**Rajalahti *et al.***[25]	**Dowdy *et al.***[26]	**Roos *et al.***[27]
Cost	Laboratories expenditures, quotation of distributors	Hospital record, Retrospective patient data	Observational data	Interview (data incurred by patient), published data (IDA, National Leprosy and TB Program), Operational data from the clinic

Please see S1 Table for list of references
