# Supplementary material for: Disparities in model-based cost-effectiveness analyses of tuberculosis diagnosis: A systematic review
Source: PLoS One. 2018 May 9;13(5):e0193293. doi: 10.1371/journal.pone.0193293 (PMC5942841; doi:10.1371/journal.pone.0193293)
Supplement: S3 Table — (PDF) [file pone.0193293.s005.pdf]

S1 Table. Data Extraction for Main Outcomes

**Authors**	**Diagnosis**	**QALY**	**DALY averted**	**Other health Outcome**	**Cost**	**ACER**	**ACER adjusted to current value (2015)**	**ICER**	**ICER adjusted to current value (2015)**	**Conclusion**
**Kelly *et al.*, 2015**[1]	ZN microscopy		583992		$2,864,685					
	LED microscopy		588811		$2,935,235			$14.64	$15.11	Cost Effective
**Little *et al.*, 2015**[2]	Clinical Exam+ sputum smear vs. Clinical Exam		451200		$9,769,000			$21.65	$22.03	
	Clinical Exam + Xpert vs. Clinical Exam		100600		$34,721,100			$345.14	$351.16	
	Clinical Exam + IGRA vs. Clinical Exam		-70400		$14,625,000			-$207.74	-$211.36	Clinical Exam + IGRA is dominated by base case
**Suen *et al.*, 2015**[3]	Status quo-No Intervention	24.740			$506.87					
	Xpert for DST	24.743			$507.27			Dominated		
	PPM	24.754			$507.87			$71.43	$72.67	PPM should be prioritized
	PPM+Xpert for DST	24.756			$508.12			$125.00	$127.18	
	Xpert for all Diagnosis	24.764			$523.61			Dominated		
	PPM+Xpert for all diagnosis	24.771			$524.57			$1,096.67	$1,115.78	
**You *et al.*,2015**[4]	AFB smear + Xpert	8.148			$246					
	Xpert	8.289			$260			$99	$99.12	Cost Effective
	Conventional	7.992			$417			-$529	-$529.63	Dominated

Authors	Diagnosis Approach	QALY	DALY averted	Other health Outcome	Cost	ACER	ACER adjusted to current value (2015)	ICER	ICER adjusted to current value (2015)	Conclusion
**Zwerling *et al.*, 2015**[5]										
LED	No of test/year:									
	50		0.064		$116	$1,812.50	$1,970.11	Reference		Cost effective in moderate patient volume and TB prevalence
	100		0.064		$78	$1,218.75	$1,324.73	Reference		
	1000		0.064		$45	$703.13	$764.27	Reference		
Xpert	No of test/year:									
	50		0.122		$343	$2,811.48	$3,055.95	$3,913.79	$4,254.11	Cost effective in high patient volume and TB prevalence
	100		0.122		$199	$1,631.15	$1,772.99	$2,086.21	$2,267.62	
	1000		0.122		$69	$565.57	$614.75	$413.79	$449.77	
**Langley *et al.*, 2014**[6]	A2 LED Fluorescence Microscopy		57900		$1,700,000	$29.36	$30.31	$29.36	$30.31	
	A3 Same day LED Microscopy		151700		$5,700,000	$37.57	$38.78	$42.64	$44.02	
	B1 Full Xpert rollout		346000		$36,900,000	$106.65	$110.10	$160.58	$165.77	Cost-effective (Below WTP threshold)
	B2 Xpert for known HIV + cases		84800		$13,100,000	$154.48	$159.47			Dominated
	B3 Xpert for HIV + cases with additional HIV testing		162800		$22,100,000	$135.75	$140.14			Dominated
	C1 Xpert for smear - and HIV + cases		68800		$12,600,000	$183.14	$189.06			Dominated

Authors	Diagnosis	QALY	DALY averted	Other health Outcome		Cost	ACER	ACER adjusted to current value (2015)	ICER	ICER adjusted to current value (2015)	Conclusion
	C2 Xpert for smear - and HIV + cases with additional HIV testing		94700			$19,600,000	$206.97	$213.66			Dominated
**Schmid *et al.*, 2014**[7]	Smear			26	Detected case	$0.22	$0.01	$0.01			
	Culture			51	Detected case	$19.28	$0.38	$0.48			Dominated
	Detect			41	Detected case	$1.60	$0.04	$0.05			Dominated
	Smear + Detect			48	Detected case	$1.59	$0.03	$0.04	$0.06	$0.08	Cost-effective
	Smear + Culture			53	Detected case	$17.15	$0.32	$0.40	$3.11	$3.90	
**Choi *et al.*, 2013**[8]	Algorithm 1: No Molecular	22.086				$2,727.68					
	Algorithm 2: Selective MTD	22.088				$2,479.63					
	Algorithm 3: Intensive MTD	22.091				$2,653.08			$47,914	$49,463.11	
	Algorithm 4: Selective Xpert	22.088				$2,481.71			$48,547	$50,116.57	
	Algorithm 5: Intensive Xpert	22.093				$2,672.79			$40,312	$41,615.33	Cost Effective
**Guerra *et al.*,2013**[9]	E1: Smear for those with CXR suggestive of TB			74	Correct TB diagnosis	$5,368	$72.55	$74.90			
	E2: CXR for those with 1 smear positive or 2 smears negative			96	Correct TB diagnosis	$5,944	$61.92	$63.92			

Authors	Diagnosis	QALY	DALY averted	Other health Outcome	Cost	ACER	ACER adjusted to current value (2015)	ICER	ICER adjusted to current value (2015)	Conclusion
	E3: Smear and CXR as first test for all			96 Correct TB diagnosis	$5,442	$56.69	$58.52			Cost Effective
**Shah *et al.*, 2013**[10]	Smear		Reference		$707,600			Reference		
	Smear + LF-Lam		3191		$813,200			$33.09	$33.67	Cost Effective
	Xpert		4757		$983,100			$57.91	$58.92	
	Xpert + LF LAM		5982		$1,046,900			$56.72	$57.71	Cost Effective
	Xpert+ LF LAM vs Xpert		1225		$63,800			$52.08	$52.99	
**Sun *et al.*, 2013**[11]										
South Africa	Current Practice		Reference		$299,000					
	Current Practice + LAM		224		$378,000			$353	$383.70	Cost Effective
Uganda	Current Practice		Reference		$64,000					
	Current practice + LAM		224		$83,000			$85	$92.39	Cost Effective
**van't Hoog, *et al.*, 2013**[12]										
Uganda	**Triage test:** 95% sensitivity, 75% specificity, Cost $5		12102		$309,250	$26	$27.40			Triage test improve affordability of Xpert for TB diagnosis
	**Xpert for all presumptive TB**		12306		$408,895	$33	$34.77	$488	$514.20	

Authors	Diagnosis	QALY	DALY averted	Other health Outcome	Cost	ACER	ACER adjusted to current value (2015)	ICER	ICER adjusted to current value (2015)	Conclusion
	**Triage test:** 85% sensitivity, 85% specificity, Cost $5		11566		$280,460	$24	$25.29			Triage test improve affordability of Xpert for TB diagnosis
	**Xpert for all presumptive TB**		12306		$408,895	$33	$34.77	$174	$183.34	
India	**Triage test:** 95% sensitivity, 75% specificity, Cost $5		9904		$345,754	$35	$36.88			Triage test improve affordability of Xpert for TB diagnosis
	**Xpert for all presumptive TB**		10009		$415,720	$42	$44.26	$666	$701.76	
	**Triage Test:** 85% sensitivity, 85% specificity, Cost $5		9568		$321,493	$34	$35.83			Triage test improve affordability of Xpert for TB diagnosis
	**Xpert for all presumptive TB**		10009		$415,720	$42	$44.26	$214	$225.49	
South Africa	**Triage test:** 95% sensitivity, 75% specificity, Cost $5		10176		$864,481	$85	$89.56			Triage test improve affordability of Xpert for TB diagnosis
	**Xpert for all presumptive TB**		10351		$985,767	$95	$100.10	$693	$730.21	

Authors	Diagnosis	QALY	DALY averted	Other health Outcome		Cost	ACER	ACER adjusted to current value (2015)	ICER	ICER adjusted to current value (2015)	Conclusion
	**Triage test:** 85% sensitivity, 85% specificity, Cost $5		9719			$807,940	$83	$87.46			Triage test improve affordability of Xpert for TB diagnosis
	**Xpert for all presumptive TB**		10351			$985,767	$95	$100.10	$281	$296.09	
**Abimbola *et al.*, 2012**[13]	Recommended practice with Xpert vs. Current Practice			1.04	Incremental death averted	-$41,128			-$39,546.15	-$42,984.87	Current Practice Dominated
	Recommended practice with Culture vs. Xpert			1.16	Incremental death averted	$69,990			$60,336.21	$65,582.73	Cost Effective
**Menzies *et al.*, 2012**[14]											
**South Africa**											Introduction to Xpert is cost effective (below WTP threshold)
10 years	Xpert Price $20 vs. Status quo		480000			$401,000,000			$835.42	$880.28	
	Xpert Price $ 30 vs. Status quo		480000			$460,000,000			$958.33	$1,009.79	
	Xpert Price $40 vs. Status quo		480000			$520,000,000			$1,083.33	$1,141.50	
20 years	Xpert Price $20 vs. Status quo		1550000			$1,103,000,000			$711.61	$749.82	

Authors	Diagnosis	QALY	DALY averted	Other health Outcome	Cost	ACER	ACER adjusted to current value (2015)	ICER	ICER adjusted to current value (2015)	Conclusion
	Xpert Price $ 30 vs. Status quo		1550000		$1,217,000,000			$785.16	$827.32	
	Xpert Price $40 vs. Status quo		1550000		$1,330,000,000			$858.06	$904.13	
**Dowdy *et al.*, 2011**[15]	*Diagnosis is performed with no other microbiological test*									
	Sputum smear		623000		$11,900,000	$19.10	$20.65			
	anda-tb		520000		$47,500,000	$91	$98.91	-$345.63	-$375.68	Dominated
	Diagnosis is added to sputum smear microscopy									
	MGIT culture		130000		$27,600,000	$212.31	$230.43			
	anda-tb		110000		$39,000,000	$355	$385.87	-$570.00	-$619.56	Dominated
**Hughes *et al.*, 2011**[16]	SSM followed by culture when SSM +	0.842			£233.65					
	SSM followed by culture every time	0.844			£258.85			£12,600	$19,795	Cost Effective
	SSM followed by culture when SSM -	0.844			£260.51			Dominated		
	SSM and NAAT when SSM +, otherwise culture	0.844			£273.67			Dominated		

Authors	Diagnosis	QALY	DALY averted	Other health Outcome	Cost	ACER	ACER adjusted to current value (2015)	ICER	ICER adjusted to current value (2015)	Conclusion
	SSM and NAAT when SSM -, otherwise culture	0.845			£349.57			£90,720	$142,522	
	NAAT and Culture when NAAT +	0.844			£350.66			Dominated		
	NAAT only	0.844			£352.88			Dominated		
	SSM and NAAT, culture when discrepancy between result	0.845			£364.41			Dominated		
	NAAT followed by culture every time	0.846			£374.56			£24,990	$39,260	
	NAAT and Culture when NAAT -	0.846			£376.79			Dominated		
	SSM and NAAT followed by culture every time	0.846			£390.50			Dominated		
**Vassal *et al*., 2011**[17]										
India	Base case		17133		$513,698	$29.98	$32.59			
	Xpert as addition		19887		$664,191	$33.40	$36.30	$54.65	$59.40	
	Replacement with Xpert		20019		$709,248	$35.43	$38.51	$67.76	$73.65	

Authors	Diagnosis	QALY	DALY averted	Other health Outcome	Cost	ACER	ACER adjusted to current value (2015)	ICER	ICER adjusted to current value (2015)	Conclusion
	(compare to base case)									
	Replacement with Xpert (compare to Xpert as addition)		20019		$709,248	$35.43	$38.51	$341.34	$371.02	Cost-effective (Below WTP threshold)
South Africa	Base case		15805		$1,084,698	$68.63	$74.60			
	Xpert as addition		20420		$1,594,276	$78.07	$84.86	$110.42	$120.02	
	Replacement with Xpert (compare to base case)		20702		$1,758,467	$84.94	$92.33	$137.59	$149.55	
	Replacement with Xpert (compare to Xpert as addition)		20702		$1,758,467	$84.94	$92.33	$582.24	$632.87	Cost-effective (Below WTP threshold)
Uganda	Base case		22182		$544,499	$24.55	$26.68			
	Xpert as addition		24570		$643,172	$26.18	$28.46	$41.32	$44.91	
	Replacement with Xpert (compare to base case)		24611		$670,137	$27.23	$29.60	$51.72	$56.21	
	Replacement with Xpert (compare to Xpert as addition)		24611		$670,137	$27.23	$29.60	$657.68	$714.87	Cost-effective (Below WTP threshold)
**Chihota *et al.*, 2010**[18]	**MGIT vs LJ**									Higher yield and higher cost

Authors	Diagnosis	QALY	DALY averted	Other health Outcome	Cost	ACER	ACER adjusted to current value (2015)	ICER	ICER adjusted to current value (2015)	Conclusion
	Identification method:									
	Standard biochemical			*M. tuberculosis* isolate identified				$504.08	$576.22	
	Anti-MPB64			*M. tuberculosis* isolate identified				$397.67	$454.58	
	Cording			*M. tuberculosis* isolate identified				$328.10	$375.06	
	**MGIT+LJ vs LJ**									Higher yield and higher cost
	Identification method:									
	Standard biochemical			*M. tuberculosis* isolate identified				$160.80	$183.81	
	Anti-MPB64			*M. tuberculosis* isolate identified				$129.53	$148.07	
	Cording			*M. tuberculosis* isolate identified				$109.07	$124.68	
**Bonnet *et al.*, 2010**[19]	B1			0.23 % of smear positive case	€ 2.01					
B: bleach sedimentation	D1 + D2			0.21 % of smear positive case	€ 2.70			Dominated		
D: Direct smear	D1 + B1 + D2			0.24 % of smear positive case	€ 2.93			€ 184.00	$250.31	
1/2: Sample number	D1 + B1			0.24 % of smear positive case	€ 2.94			Dominated		
	B1 + D2			0.24 % of smear positive case	€ 3.03			Dominated		
	D1 + B2			0.25 % of smear positive case	€ 3.01			€ 8.89	$12.09	
	B1 + B2			0.26 % of smear positive	€ 3.33			€ 26.67	$36.28	Cost Effective

Authors	Diagnosis	QALY	DALY averted	Other health Outcome		Cost	ACER	ACER adjusted to current value (2015)	ICER	ICER adjusted to current value (2015)	Conclusion
					case						
	D1 + D2 + B2			0.25	% of smear positive case	€ 3.96			Dominated		
	D1 + B1 + B2			0.26	% of smear positive case	€ 4.00			Dominated		
	B1 + D2 + B2			0.25	% of smear positive case	€ 4.25			Dominated		
**Scherer *et al.*,2009**[20]	AFB smear + culture			111	No. of accurately diagnosed TB	$5,635,760	$50,772.61	$63,642.78			
	AFB smear + dot blot			109	No. of accurately diagnosed TB	$1,498,660	$13,749.17	$17,251.39			Cost Effective
**Dowdy *et al.*, 2008 (1)**[21]											
Low throughput	Baseline (No TB culture)		Reference			$130,000					
	TB Culture: Solid LJ		37			$166,000			$973	$1,112.26	
	TB Culture: MGIT		45			$188,000			$2,750	$3,143.58	Cost-effective (Below WTP threshold)
High throughput	Baseline (No TB culture)		Reference			$130,000					
	TB Culture: Solid LJ		37			$145,000			$405	$462.96	
	TB Culture: MGIT		45			$161,000			$2,000	$2,286.24	Cost-effective (Below WTP threshold)
**Dowdy *et al.*,2008 (2)**[22]											
New test: sensitivity 90%, specificity 95%, price $1/test											

Authors	Diagnosis	QALY	DALY averted	Other health Outcome	Cost	ACER	ACER adjusted to current value (2015)	ICER	ICER adjusted to current value (2015)	Conclusion
South Africa	Sputum smear alone		215		$18,596	$86.49	$101.68	Reference		
	New test alone		257		$50,899	$198.05	$232.84	$769.12	$904.24	
	Smear+ Culture		280		$39,648	$141.60	$166.48	$323.88	$380.78	
	Smear + New test		321		$62,441	$194.52	$228.69	$413.63	$486.30	Potentially cost effective
Brazil	Sputum smear alone		166		$21,768	$131.13	$154.17	Reference		
	New test alone		194		$53,552	$276.04	$324.53	$1,135.14	$1,334.56	
	Smear+ Culture		213		$41,045	$192.70	$226.55	$410.15	$482.20	
	Smear + New test		243		$70,185	$288.83	$339.57	$628.79	$739.26	Potentially cost effective
Kenya	Sputum smear alone		531		$20,075	$37.81	$44.45	Reference		
	New test alone		631		$52,731	$83.57	$98.25	$326.56	$383.93	
	Smear+ Culture		687		$41,738	$60.75	$71.42	$138.87	$163.27	
	Smear + New test		790		$63,911	$80.90	$95.11	$169.25	$198.98	Potentially cost effective
**Guerra *et al.*, 2008**[23]	i "CDC"			447 No. of correct diagnosis	$27,759	$62.13	$71.02			
	ii "Simultaneous"			471 No. of correct diagnosis	$46,518	$98.69	$112.81			
	iii "Smear positive dilution"			462 No. of correct diagnosis	$23,259	$50.39	$57.60			Cost Effective
	iv "Sequential"			471 No. of correct diagnosis	$41,070	$87.13	$99.60			
**Mueller *et al.*, 2008**[24]	Home-Made LJ			*positive M. Tuberculosis specimen*		$340	$399.73			
	Commercial LJ			*positive M. Tuberculosis*		$312	$366.81			

Authors	Diagnosis	QALY	DALY averted	Other health Outcome	Cost	ACER	ACER adjusted to current value (2015)	ICER	ICER adjusted to current value (2015)	Conclusion
				specimen						
	Manual MGIT			*positive M. Tuberculosis specimen*		$197	$231.61			Cost Effective
	Automatic MGIT			*positive M. Tuberculosis specimen*		$202	$237.49			Cost Effective
**Rajalahti *et al.*, 2004**[25]	Routine PCR for all vs No PCR			additional correct treatment			€ 1,970	$2,729.88		Not cost saving
				additional correct isolation			€ 2,011	$2,786.69		Not cost saving
	Routine PCR only for smear positive			0.97 Probability of correct treatment	€ 130.00					Cost Effective
	PCR			0.96 Probability of correct treatment	€ 225.40			-€ 9,540	-€ 13,219.81	Dominated
	Routine PCR only for smear positive			0.03 Probability of correct isolation	€ 130					Cost Effective
	PCR			0.02 Probability of correct isolation	€ 225.40			-€ 9,540	-€ 13,219.81	Dominated
**Dowdy *et al.*, 2003**[26]	MTD			68 Early Exclusion per 100 patients	$33,800	$497.06	$665.23			Not cost saving
**Roos *et al.*, 1998**[27]	Routine Diagnostic			347 Total number of correctly diagnosed	$13,985	$40	$59.07			

Authors	Diagnosis	QALY	DALY averted	Other health Outcome	Cost	ACER	ACER adjusted to current value (2015)	ICER	ICER adjusted to current value (2015)	Conclusion
	Procedure			*M. Tuberculosis*						
	PCR			374 Total number of correctly diagnosed *M. Tuberculosis*	$26,697	$71	$104.85			Cost Effective

Please see S1 Table for list of references
