# Supplementary material for: Disparities in model-based cost-effectiveness analyses of tuberculosis diagnosis: A systematic review
Source: PLoS One. 2018 May 9;13(5):e0193293. doi: 10.1371/journal.pone.0193293 (PMC5942841; doi:10.1371/journal.pone.0193293)
Supplement: S4 Table — (PDF) [file pone.0193293.s006.pdf]

S4 Table 1. Data Extraction of Tuberculosis Progression Modeling Approaches (1)

No.		Authors	**Kelly *et al*., 2015**[1]		**Little *et al*., 2015**[2]		**Suen *et al*., 2015**[3]		**You *et al*., 2015**[4]		**Zwerling *et al*., 2015**[5]	
		Questions	Is it modeled?	Details	Is it modeled?	Details	Is it modeled?	Details	Is it modeled?	Details	Is it modeled?	Details
**1**		**Initial Prevalence**										
	A	Is the initial prevalence data taken from the appropriate population?	Yes		Yes		Yes		Yes		Yes	
	B	Is there any stratification of TB sates based on certain risk factor, such as age, social, and/or spatial heterogeneity? (YES/NO)	No		No		Yes	TB states were stratified by age and gender	No		No	
**2**		**Progression from susceptible state following TB infection**										
	A	Is acute and chronic progression to active disease modeled? (YES/NO)	No		No		Yes		No		No	
	B	How is the acute and chronic progression modeled?										
	i	A proportion of patients may progress directly to active disease (acute), while the other progress to latency/chronic state (no stratification of latency state)										
	ii	All patients progress to early latent state, and from the state they can progress to active disease (acute) or late latency (chronic)										
	iii	A proportion of patients may progress to fast progression state (acute), while other progresses to slow progression state (chronic)										
	iv	Other						Patient occupied latent states (drug sensitive or resistant) following an infection and could progress to active TB (drug sensitive or resistant) or stay in latency. Two types of progression rates were applied, i.e. fast and slow progression rate				
**3**		**Latency/Chronic State Progression**										
	A	Is accelerated progression from latency/chronic disease to active disease modeled? (YES/NO)	No		No		Yes		No		No	
	B	What is the determinant of latency/chronic accelerated progression to active disease?										
	i	Progression is due to exogenous reinfection										
	ii	Progression is due to reactivation of the latent infection (e.g. in less immunocompetent patient)					√	Re-infection was not modeled explicitly. Progression rate captured re-infection process with the same infecting strain. Re-infection with different TB strain was not possible.				
	iii	Both i and ii										
	iv	Other										
	v	No clear determinant is explained										
**4**		**Active Disease**										

No.	Authors	**Kelly *et al.*, 2015**[1]		**Little *et al.*, 2015**[2]		**Suen *et al.*, 2015**[3]		**You *et al.*, 2015**[4]		**Zwerling *et al.*, 2015**[5]	
	Questions	Is it modeled?	Details	Is it modeled?	Details	Is it modeled?	Details	Is it modeled?	Details	Is it modeled?	Details
A	Are the patients with active disease categorized based on sputum smear results ? (YES/NO)	No		Yes	Smear status affected diagnostic accuracy, and probability of generating secondary cases	No		Yes	Smear status influenced the necessity for additional diagnosis and diagnostic accuracy	Yes	Smear status affected diagnostic accuracy
B	Is transmission of active pulmonary disease modeled? (YES/NO)	No		No	The active process of transmission was not modeled. However, number of secondary cases were calculated from numbers of undiagnosed and untreated TB cases	Yes		No		No	
C	Is the transmission probability stratified based on sputum smear result (negative smear has lower transmission probability)? (YES/NO)	No		Yes	Smear negative cases generated less secondary cases	No		No		No	
D	<u>What is the mode of transmission?</u>					-	-				
i	Contact transmission					√	Contact pattern was age specific				
ii	Household transmission										
iii	Other										
**5**	**MDR TB**										
A.	Is MDR TB incorporated in the model? (YES/NO)	No		Yes	Prevalence of MDR TB was accounted in the model. Cost and consequences of MDR TB was measured only in Xpert arm	Yes		Yes		Yes	
B	<u>How is MDR TB modeled?</u>					-	-				
i	Prevalence of MDR TB is considered			√				√		√	
ii	An exclusive state of MDR TB is modeled					√					
iii	Other										
C	<u>What are the impacts of MDR TB?</u>					-	-				
i	Higher treatment cost (second line)			√		√		√		√	
ii	Higher rate of failure					√				√	Second line treatment has higher rate of failure compare to first line treatment for smear positive TB
iii	Lower transmission rate										
iv	Other					√	Higher rate of treatment default and mortality rate				
D	Is diagnosis/screening strategy impact detection of MDR TB			Yes		√		Yes		Yes	
E	How does the diagnostic/screening strategy impact the detection of MDR TB				Only Xpert was able to detect drug resistance. Hence, cost and consequences were only observed in this arm. Details of the consequences were not found in the report		Xpert detected drug resistant faster, thus it allowed faster treatment with correct second line drug		Xpert detected drug resistant faster, thus it allowed faster treatment with correct second line drug and avoid fatality		Xpert detected drug resistant faster, thus it allowed faster treatment with correct second line drug and avoid fatality due to treatment failure
**6**	**Natural Recovery**										

No.	Authors	Kelly *et al.*, 2015[1]		Little *et al.*, 2015[2]		Suen *et al.*, 2015[3]		You *et al.*, 2015[4]		Zwerling *et al.*, 2015[5]	
	Questions	Is it modeled?	Details	Is it modeled?	Details	Is it modeled?	Details	Is it modeled?	Details	Is it modeled?	Details
A	Is natural recovery from TB active disease modeled? (YES/NO)	No		No		No		No		No	
B	What are the consequences of TB natural recovery?						Not detailed . In general, cured patient could progress to susceptible or latent TB states				
i	Completely clear infection (return to susceptible)										
ii	Return to latent state										
iii	Combination of a and b										
iv	Other										
**7**	**Treatment**										
	Active Disease										
A	Is treatment of active disease modeled? (YES/NO)	Yes		Yes		Yes			Yes	Yes	
B	Which of the following treatment outcomes are incorporated to the model?					-	-				
i	Not receiving treatment	√	A proportion of diagnosed patients did not receive treatment	√	Undiagnosed and untreated cases resulted in secondary cases	√		√	Patients who did not receive early treatment based on initial test, would receive late/delayed treatment following positive culture result	√	Undiagnosed and untreated cases resulted in fatality
ii	Completion/treated successfully	√		√		√		√		√	Probability of treatment success depended on type of drug (1st or 2nd line), smear status, as well as resistance status
iii	Failure					√				√	
iv	Lost to Follow Up (Non-compliance)					√				√	
C	What are the consequences of the treatment?					-	-				
C.1	Complete Treatment:					-	-				
i	Long-life Protection, out from the model	√	No re-infection or relapse cases were modeled. Those who started treatment were assumed to be cured after a year	√	No re-infection or relapse cases were modeled			√	No re-infection or relapse cases were modeled	√	No re-infection or relapse was modeled
ii	Back to susceptible, rate of reinfection considered the same with primary infection										
iii	Progress to recovered state with partial immunity against reinfection										
iv	Return to latency										
v	Other					√	Cured patients could progress to susceptible or latent TB states (no longer infectious)	√	Patients receiving early and late treatment were subjected to survival or death		
C.2	Failure					-	-				
i	Return to latency										
ii	MDR TB					√					
iii	Category II										
iv	Other					√	Return to active				Fatality

No.	Authors	**Kelly *et al.*, 2015**[1]		**Little *et al.*, 2015**[2]		**Suen *et al.*, 2015**[3]		**You *et al.*, 2015**[4]		**Zwerling *et al.*, 2015**[5]	
	Questions	Is it modeled?	Details	Is it modeled?	Details	Is it modeled?	Details	Is it modeled?	Details	Is it modeled?	Details
							disease				
C.3	Lost to Follow Up (Non-compliance)					-	-				
i	Return to latency										
ii	MDR TB					√					
iii	Category II										
iv	Other					√	Return to active disease				Patients could be re-diagnosed and re-treated. Those who were not re-diagnosed remained untreated and assumed to be fatal.
**8**	**BCG Vaccination**										
A	Is BCG vaccinated population incorporated to the model? (YES/NO)	No		No		No		No		No	
B	What is the impact of BCG vaccination towards infection probability?						-				
i	Partial protection against acquiring disease										
ii	Reduced probability in disease progression										
iii	Combination of a and b										
iv	Other										
C	Is BCG vaccination status affecting diagnosis/screening result?										
**9**	**Comorbidity**										
A	Is comorbidity incorporated into the model? (e.g. HIV, Diabetes) (YES/NO)	No		Yes		No		No		Yes	
B	How is the comorbidity incorporated into the model?						-				
i	Select only the population with the specific comorbidity (e.g. patients with HIV)									√	Newly diagnosed HIV positive patients
ii	Natural progression of comorbidity is modeled alongside TB and integration is depicted in several states										
iii	Comorbidity is presented as integrated states with TB										
iv	Comorbidity incidence/prevalence considered as input parameters in the model			√							
v	Other										
C	What are the impact of comorbidity towards TB disease?						-				
i	Alter rate of infection										
ii	Alter rate/probability of progression to active disease										
iii	Promote latency reactivation										
iv	Influence diagnosis accuracy (higher probability detected as smear negative)									√	
v	Alter probability of acquiring MDR TB										
vi	Other			√	HIV status affected mortality rate					√	HIV status affected mortality rate
**10**	**Mortality**					-	-				
A	Is mortality incorporated as one of the endpoints in the model? (YES/NO)	No		Yes		Yes		Yes		Yes	
B	What type of mortality incorporated into the model?					-	-				
i	TB-specific death			√				√	Monthly mortality rate for hospitalized adult with TB was incorporated in the model. Late treatment	√	

No.	Authors	Kelly *et al.*, 2015[1]		Little *et al.*, 2015[2]		Suen *et al.*, 2015[3]		You *et al.*, 2015[4]		Zwerling *et al.*, 2015[5]	
	Questions	Is it modeled?	Details	Is it modeled?	Details	Is it modeled?	Details	Is it modeled?	Details	Is it modeled?	Details
									was associated with higher mortality rate		
ii	TB-specific death and other background mortality (as well as death due to comorbidity)					√	Background mortality was stratified by age and gender				
**11**	**Extra pulmonary Tuberculosis**										
A	Is extra pulmonary TB incorporated into the model? (YES/NO)	No		No		No		No		No	

S4 Table 2. Data Extraction of Tuberculosis Progression Modeling Approaches (2)

No.	Authors	**Langley *et al.*, 2014**[6]		**Schmid *et al.*, 2014**[7]		**Choi *et al.*, 2013**[8]		**Guerra *et al.*, 2013**[9]		**Shah *et al.*, 2013**[10]		**Sun *et al.*, 2013**[11]	
	Questions	Is it modeled?	Details	Is it modeled?	Details	Is it modeled?	Details	Is it modeled?	Details	Is it modeled?	Details	Is it modeled?	Details
**1**	**Initial Prevalence**												
A	Is the initial prevalence data taken from the appropriate population?	Yes		Yes		Yes		Yes		Yes		Yes	
B	Is there any stratification of TB sates based on certain risk factor, such as age, social, and/or spatial heterogeneity? (YES/NO)	Yes	TB states were stratified by age	No		No		No		No		No	
**2**	**Progression from susceptible state following TB infection**												
A	Is acute and chronic progression to active disease modeled? (YES/NO)	Yes		No		No		No		No		No	
B	How is the acute and chronic progression modeled?												
i	A proportion of patients may progress directly to active disease (acute), while the other progress to latency/chronic state (no stratification of latency state)												
ii	All patients progress to early latent state, and from the state they can progress to active disease (acute) or late latency (chronic)	√											
iii	A proportion of patients may progress to fast progression state (acute), while other progresses to slow progression state (chronic)												
iv	Other												
**3**	**Latency/Chronic State Progression**												
A	Is accelerated progression from latency/chronic disease to active disease modeled? (YES/NO)	Yes		No		No		No		No		No	
B	What is the determinant of latency/chronic accelerated progression to active disease?												
i	Progression is due to exogenous reinfection												
ii	Progression is due to reactivation of the latent infection (e.g. in less immunocompetent patient)												
iii	Both i and ii	√											
iv	Other												
v	No clear determinant is explained												
**4**	**Active Disease**												
A	Are the patients with active disease categorized based on sputum smear results ? (YES/NO)	Yes	Smear status affected diagnostic accuracy	No		Yes	Smear status affected diagnostic accuracy	Yes	Sputum smear status influenced type of secondary test	Yes	Smear status was influenced by HIV status and affected diagnostic accuracy	Yes	Smear status influenced the necessity for additional diagnosis
B	Is transmission of active pulmonary disease modeled? (YES/NO)	yes		No		No		No		No		No	
C	Is the transmission probability stratified based on sputum smear result (negative smear has lower transmission probability)? (YES/NO)	Yes				No		No		No		No	
D	What is the mode of transmission?												
i	Contact transmission	√											
ii	Household transmission												
iii	Other												

No.	Authors	Langley *et al.*, 2014[6]		Schmid *et al.*, 2014[7]		Choi *et al.*, 2013[8]		Guerra *et al.*, 2013[9]		Shah *et al.*, 2013[10]		Sun *et al.*, 2013[11]	
	Questions	Is it modeled?	Details	Is it modeled?	Details	Is it modeled?	Details	Is it modeled?	Details	Is it modeled?	Details	Is it modeled?	Details
**5**	**MDR TB**												
A.	Is MDR TB incorporated in the model? (YES/NO)	Yes	Active transmission of MDR TB was not modeled	No		Yes		No		Yes		No	
B	<u>How is MDR TB modeled?</u>												
i	Prevalence of MDR TB is considered	√				√				√			
ii	An exclusive state of MDR TB is modeled												
iii	Other												
C	<u>What are the impacts of MDR TB?</u>												
i	Higher treatment cost (second line)	√				√				√			
ii	Higher rate of failure	√	Higher rate of failure In patients who were incorrectly treated with first line treatment							√	Higher rate of failure In patients who were incorrectly treated with first line treatment		
iii	Lower transmission rate												
iv	Other					√	Higher mortality rate and different utility weight for patient receiving MDR treatment			√	Higher mortality rate, especially in those who received incorrect treatment		
D	Is diagnosis/screening strategy impact detection of MDR TB	Yes				√				Yes			
E	How does the diagnostic/screening strategy impact the detection of MDR TB		Xpert detected drug resistant faster, thus it allowed faster treatment with correct second line drug				Xpert detected drug resistant faster, thus it allowed faster treatment with correct second line drug				Xpert detected drug resistant faster, thus it allowed faster treatment with correct second line drug		
**6**	**Natural Recovery**												
A	Is natural recovery from TB active disease modeled? (YES/NO)	Yes		No		No		No		No		No	
B	What are the consequences of TB natural recovery?												
i	Completely clear infection (return to susceptible)												
ii	Return to latent state												
iii	Combination of a and b												
iv	Other	√	Patients progress to recovered state, which has partial immunity against re-infection										
**7**	**Treatment**												
	Active Disease												

No.	Authors	**Langley *et al.*, 2014**[6]		**Schmid *et al.*, 2014**[7]		**Choi *et al.*, 2013**[8]		**Guerra *et al.*, 2013**[9]		**Shah *et al.*, 2013**[10]		**Sun *et al.*, 2013**[11]	
	Questions	Is it modeled?	Details	Is it modeled?	Details	Is it modeled?	Details	Is it modeled?	Details	Is it modeled?	Details	Is it modeled?	Details
A	Is treatment of active disease modeled? (YES/NO)	Yes		Yes		Yes		No		Yes		Yes	
B	Which of the following treatment outcomes are incorporated to the model?												
i	Not receiving treatment	√				√	Undiagnosed patients had higher mortality rate and different utility weight			√	Undiagnosed patients could be re-diagnosed. Undiagnosed patients were subjected to higher mortality rate. A proportion of these patients could convert to smear positive upon re-diagnosis	√	
ii	Completion/treated successfully	√		√		√	All patients receiving treatment were presumed to successfully completed the treatment			√		√	
iii	Failure	√								√		√	
iv	Lost to Follow Up (Non-compliance)	√					Non-adherence was assumed to be negligible			√			
C	What are the consequences of the treatment?			Treatment consequences were not modeled, however the cost for treatment was accounted									
C.1	Complete Treatment:												
i	Long-life Protection, out from the model					√	No re-infection or relapse cases were modeled			√	No re-infection or relapse cases were modeled	√	No re-infection or relapse cases were modeled
ii	Back to susceptible, rate of reinfection considered the same with primary infection												
iii	Progress to recovered state with partial immunity against reinfection	√											
iv	Return to latency												
v	Other												
C.2	Failure												
i	Return to latency										Not detailed		
ii	MDR TB												
iii	Category II												
iv	Other	√	Patients re-enter the treatment cycle										
C.3	Lost to Follow Up (Non-compliance)										Not detailed		

**No.**	Authors	**Langley *et al.*, 2014**[6]		**Schmid *et al.*, 2014**[7]		**Choi *et al.*, 2013**[8]		**Guerra *et al.*, 2013**[9]		**Shah *et al.*, 2013**[10]		**Sun *et al.*, 2013**[11]	
	Questions	Is it modeled?	Details	Is it modeled?	Details	Is it modeled?	Details	Is it modeled?	Details	Is it modeled?	Details	Is it modeled?	Details
i	Return to latency												
ii	MDR TB												
iii	Category II												
iv	Other	√	Patients could experience mortality, natural recovery, or remained infectious										
**8**	**BCG Vaccination**												
A	Is BCG vaccinated population incorporated to the model? (YES/NO)	No		No		No		No		No		No	
B	<u>What is the impact of BCG vaccination towards infection probability?</u>												
i	Partial protection against acquiring disease												
ii	Reduced probability in disease progression												
iii	Combination of a and b												
iv	Other												
C	Is BCG vaccination status affecting diagnosis/screening result?												
D	How does BCG affect the diagnosis/screening result?												
**9**	**Comorbidity**												
A	Is comorbidity incorporated into the model? (e.g. HIV, Diabetes) (YES/NO)	Yes		No	Patients were reported to be infected with HIV or not; but no further elaboration on HIV prevalence and their influence on TB	Yes		No	Not directly incorporated in the model's structure. Only stated that 12% of the population was assumed to be HIV positive. However, the influence of HIV towards TB progression was not elaborated.	Yes		Yes	
B	<u>How is the comorbidity incorporated into the model?</u>												
i	Select only the population with the specific comorbidity (e.g. patients with HIV)									√		√	
ii	Natural progression of comorbidity is modeled alongside TB and integration is depicted in several states	√											
iii	Comorbidity is presented as integrated states with TB												
iv	Comorbidity incidence/prevalence considered as input parameters in the model					√							
v	Other												
C	<u>What are the impact of comorbidity towards TB disease?</u>												
i	Alter rate of infection												
ii	Alter rate/probability of progression to active disease	√											
iii	Promote latency reactivation	√											
iv	Influence diagnosis accuracy (higher probability detected as smear negative)	√				√				√			
v	Alter probability of acquiring MDR TB												

No.	Authors	Langley *et al.*, 2014[6]		Schmid *et al.*, 2014[7]		Choi *et al.*, 2013[8]		Guerra *et al.*, 2013[9]		Shah *et al.*, 2013[10]		Sun *et al.*, 2013[11]	
	Questions	Is it modeled?	Details	Is it modeled?	Details	Is it modeled?	Details	Is it modeled?	Details	Is it modeled?	Details	Is it modeled?	Details
vi	Other	√	HIV status affected partial immunity, natural cure rate, mortality rate, and relapse rate.			√	HIV status affected mortality rate and utility weight			√	HIV status affected mortality rate	√	Lower life expectancy for HIV patients acutely infected with TB. Different utility weight was assigned for HIV patients receiving and not receiving ART treatment
**10**	<u>**Mortality**</u>												
A	Is mortality incorporated as one of the endpoints in the model? (YES/NO)	Yes		No		Yes		No		Yes		Yes	
B	<u>What type of mortality incorporated into the model?</u>												
i	TB-specific death					√							
ii	TB-specific death and other background mortality (as well as death due to comorbidity)	√								√	Following TB treatment completion, life expectancy of patients was determined by HIV status	√	Following TB treatment completion, life expectancy of patients was determined by HIV status
**11**	**Extra pulmonary Tuberculosis**												
A	Is extra pulmonary TB incorporated into the model? (YES/NO)	No		No		No		No		Yes	Extra pulmonary TB cases are categorized as sputum smear negative patients	No	

S4 Table 3. Data Extraction of Tuberculosis Progression Modeling Approaches (3)

No.		Authors	van't Hoog *et al.*, 2013[12]		Abimbola *et al.*, 2012[13]		Menzies *et al.*, 2012[14]		Dowdy *et al.*, 2011[15]		Hughes *et al.*, 2011[16]
		**Questions**	Is it modeled?	Details	Is it modeled?	Details	Is it modeled?	Details	Is it modeled?	Details	Is it modeled?	Details
**1**		**Initial Prevalence**										
	A	Is the initial prevalence data taken from the appropriate population?	Yes		Yes		Yes		Yes		Yes	
	B	Is there any stratification of TB sates based on certain risk factor, such as age, social, and/or spatial heterogeneity? (YES/NO)	No		No		No		No		No	
**2**		**Progression from susceptible state following TB infection**										
	A	Is acute and chronic progression to active disease modeled? (YES/NO)	No		No		Yes		No		No	
	B	How is the acute and chronic progression modeled?										
	i	A proportion of patients may progress directly to active disease (acute), while the other progress to latency/chronic state (no stratification of latency state)					√					
	ii	All patients progress to early latent state, and from the state they can progress to active disease (acute) or late latency (chronic)										
	iii	A proportion of patients may progress to fast progression state (acute), while other progresses to slow progression state (chronic)										
	iv	Other										
**3**		**Latency/Chronic State Progression**										
	A	Is accelerated progression from latency/chronic disease to active disease modeled? (YES/NO)	No		No		Yes		No		No	
	B	What is the determinant of latency/chronic accelerated progression to active disease?										
	i	Progression is due to exogenous reinfection										
	ii	Progression is due to reactivation of the latent infection (e.g. in less immunocompetent patient)										
	iii	Both i and ii					√					
	iv	Other										
	v	No clear determinant is explained										
**4**		**Active Disease**										
	A	Are the patients with active disease categorized based on sputum smear results ? (YES/NO)	Yes	Smear status affected diagnostic accuracy	Yes	Smear status influenced the necessity for additional diagnosis	Yes	Smear status affected diagnostic accuracy	Yes	Smear status influenced the necessity for additional diagnosis and diagnostic accuracy	Yes	Smear status influenced diagnostic accuracy
	B	Is transmission of active pulmonary disease modeled? (YES/NO)	No		No		Yes		No	The active process of transmission was not modeled. However, the number of secondary cases were calculated from numbers of undiagnosed and untreated TB cases	No	The active process of transmission was not modeled. However, the number of secondary cases were calculated from delayed treatment cases (false negative)

**No.**	**Authors**	**van't Hoog *et al.*, 2013**[12]		**Abimbola *et al.*, 2012**[13]		**Menzies *et al.*, 2012**[14]		**Dowdy *et al.*, 2011**[15]		**Hughes *et al.*, 2011**[16]	
	**Questions**	Is it modeled?	Details	Is it modeled?	Details	Is it modeled?	Details	Is it modeled?	Details	Is it modeled?	Details
C	Is the transmission probability stratified based on sputum smear result (negative smear has lower transmission probability)? (YES/NO)	No		No		Yes		Yes	Smear negative cases generated less secondary cases	No	
D	What is the mode of transmission?										
i	Contact transmission					√					
ii	Household transmission										
iii	Other										
**5**	**MDR TB**										
A.	Is MDR TB incorporated in the model? (YES/NO)	Yes		No		Yes		No		Yes	
B	How is MDR TB modeled?										
i	Prevalence of MDR TB is considered	√								√	
ii	An exclusive state of MDR TB is modeled					√					
iii	Other										
C	What are the impacts of MDR TB?										
i	Higher treatment cost (second line)	√				√				√	
ii	Higher rate of failure	√	High rate of failure in patients correctly treated with second line treatment; and higher rate for those incorrectly treated with first line treatment			√	High rate of failure in patients correctly treated with second line treatment; and higher rate for those incorrectly treated with first line treatment				
iii	Lower transmission rate					√					
iv	Other										
D	Is diagnosis/screening strategy impact detection of MDR TB	√				Yes				Yes	
E	How does the diagnostic/screening strategy impact the detection of MDR TB		All investigated strategies used Xpert which identified drug resistant strain rapidly				Xpert identify resistant strain faster. However, treatment decision was based on Drug Sensitivity Testing (DST)				NAAT detected drug resistant faster, thus it allowed faster treatment with correct second line drug. All treatment decision based on initial test could be corrected by culture-based drug sensitivity testing result (gold standard)
**6**	**Natural Recovery**										
A	Is natural recovery from TB active disease modeled? (YES/NO)	Yes	Only possible for HIV negative patients	No		Yes		No		No	
B	What are the consequences of TB natural recovery?										
i	Completely clear infection (return to susceptible)	√									
ii	Return to latent state					√	Progress to the same state (latent or recovered with partial immunity from prior infection) as those				

No.	Authors	**van't Hoog *et al.*, 2013**[12]		**Abimbola *et al.*, 2012**[13]		**Menzies *et al.*, 2012**[14]		**Dowdy *et al.*, 2011**[15]		**Hughes *et al.*, 2011**[16]	
	**Questions**	Is it modeled?	Details	Is it modeled?	Details	Is it modeled?	Details	Is it modeled?	Details	Is it modeled?	Details
							recovered through treatment				
iii	Combination of a and b										
iv	Other										
**7**	**Treatment**										
	Active Disease										
A	Is treatment of active disease modeled? (YES/NO)	Yes		Yes		Yes		Yes		Yes	
B	Which of the following treatment outcomes are incorporated to the model?										
i	Not receiving treatment	√	Undiagnosed patients could be re-diagnosed after 3 months. Before re-diagnosis, patients were subjected to natural recovery or death. A proportion of the patients also converted into smear positive upon re-diagnosis	√	Undiagnosed patients had higher rate of mortality	√	Undiagnosed patients remain infectious	√	Undiagnosed and untreated cases resulted in secondary cases	√	False negative cases would be diagnosed after 99 days and received delayed treatment
ii	Completion/treated successfully	√			Treatment result was not modeled. Patients who received treatment had lower rate of mortality	√		√		√	Treatment efficacy and adherence were assumed to be 100%
iii	Failure	√	Failure category could include defaulters/loss to follow up			√		√			
iv	Lost to Follow Up (Non-compliance)					√					
C	What are the consequences of the treatment?										
C.1	Complete Treatment:										
i	Long-life Protection, out from the model	√	No re-infection or relapse cases were modeled					√	No re-infection or relapse cases were modeled	√	No re-infection or relapse cases were modeled
ii	Back to susceptible, rate of reinfection considered the same with primary infection										
iii	Progress to recovered state with partial immunity against reinfection					√	Patients progressed to latent/recovered state with partial immunity				
iv	Return to latency										
v	Other										
C.2	Failure	Not detailed									
i	Return to latency										
ii	MDR TB					√					
iii	Category II					√	Return to treatment cycle				
iv	Other					√	Return to active disease (remain infectious)	√	Die or remain infectious after year 1		
C.3	Lost to Follow Up (Non-compliance)										

No.	Authors	van't Hoog *et al.*, 2013[12]		Abimbola *et al.*, 2012[13]		Menzies *et al.*, 2012[14]		Dowdy *et al.*, 2011[15]		Hughes *et al.*, 2011[16]	
	**Questions**	Is it modeled?	Details	Is it modeled?	Details	Is it modeled?	Details	Is it modeled?	Details	Is it modeled?	Details
i	Return to latency										
ii	MDR TB					√					
iii	Category II										
iv	Other					√	Return to active disease (remain infectious)				
**8**	**BCG Vaccination**										
A	Is BCG vaccinated population incorporated to the model? (YES/NO)	No		No		No		No		No	
B	<u>What is the impact of BCG vaccination towards infection probability?</u>										
i	Partial protection against acquiring disease										
ii	Reduced probability in disease progression										
iii	Combination of a and b										
iv	Other										
C	Is BCG vaccination status affecting diagnosis/screening result?										
D	How does BCG affect the diagnosis/screening result?										
**9**	**Comorbidity**										
A	Is comorbidity incorporated into the model? (e.g. HIV, Diabetes) (YES/NO)	Yes	HIV comorbidity impact was explored in sensitivity analysis	Yes		Yes		Yes		No	
B	<u>How is the comorbidity incorporated into the model?</u>										
i	Select only the population with the specific comorbidity (e.g. patients with HIV)			√							
ii	Natural progression of comorbidity is modeled alongside TB and integration is depicted in several states					√					
iii	Comorbidity is presented as integrated states with TB										
iv	Comorbidity incidence/prevalence considered as input parameters in the model	√						√			
v	Other										
C	<u>What are the impact of comorbidity towards TB disease?</u>										
i	Alter rate of infection					√					
ii	Alter rate/probability of progression to active disease					√					
iii	Promote latency reactivation					√					
iv	Influence diagnosis accuracy (higher probability detected as smear negative)	√				√	Due to lower probability of smear negative in HIV				
v	Alter probability of acquiring MDR TB										
vi	Other	√	HIV status affected treatment success rate and mortality rate	√	Higher mortality in patients who did not receive TB treatment during ART initiation	√	HIV status affected mortality rate	√	HIV influence TB dynamics (mortality, smear status), and DALY weights		
**10**	**<u>Mortality</u>**										
A	Is mortality incorporated as one of the endpoints in the model? (YES/NO)	Yes		Yes		Yes		Yes		No	
B	<u>What type of mortality incorporated into the model?</u>	Not detailed									
i	TB-specific death			√				√			

No.	Authors	van't Hoog *et al.*, 2013[12]		Abimbola *et al.*, 2012[13]		Menzies *et al.*, 2012[14]		Dowdy *et al.*, 2011[15]		Hughes *et al.*, 2011[16]	
	**Questions**	Is it modeled?	Details	Is it modeled?	Details	Is it modeled?	Details	Is it modeled?	Details	Is it modeled?	Details
ii	TB-specific death and other background mortality (as well as death due to comorbidity)					√					
**11**	**Extra pulmonary Tuberculosis**										
A	Is extra pulmonary TB incorporated into the model? (YES/NO)	No		No		No		No		No	

S4 Table 4. Data Extraction of Tuberculosis Progression Modeling Approaches (4)

No.	Authors	**Vassall *et al.*, 2011**[17]		**Chihota *et al.*, 2010**[18]		**Bonnet *et al.*, 2010**[19]		**Scherer *et al.*, 2009**[20]		**Dowdy *et al.*, 2008 (1)**[21]		**Dowdy *et al.*, 2008 (2)**[22]	
	**Questions**	Is it modeled?	Details	Is it modeled?	Details	Is it modeled?	Details	Is it modeled?	Details	Is it modeled?	Details	Is it modeled?	Details
**1**	**Initial Prevalence**												
A	Is the initial prevalence data taken from the appropriate population?	Yes		Yes		Yes		Yes		Yes		Yes	
B	Is there any stratification of TB sates based on certain risk factor, such as age, social, and/or spatial heterogeneity? (YES/NO)	No		No		No		No		No		No	
**2**	**Progression from susceptible state following TB infection**												
A	Is acute and chronic progression to active disease modeled? (YES/NO)	No		No		No		No		No		No	
B	How is the acute and chronic progression modeled?												
i	A proportion of patients may progress directly to active disease (acute), while the other progress to latency/chronic state (no stratification of latency state)												
ii	All patients progress to early latent state, and from the state they can progress to active disease (acute) or late latency (chronic)												
iii	A proportion of patients may progress to fast progression state (acute), while other progresses to slow progression state (chronic)												
iv	Other												
**3**	**Latency/Chronic State Progression**												
A	Is accelerated progression from latency/chronic disease to active disease modeled? (YES/NO)	No		No		No		No		No		No	
B	What is the determinant of latency/chronic accelerated progression to active disease?												
i	Progression is due to exogenous reinfection												
ii	Progression is due to reactivation of the latent infection (e.g. in less immunocompetent patient)												
iii	Both i and ii												
iv	Other												
v	No clear determinant is explained												
**4**	**Active Disease**												
A	Are the patients with active disease categorized based on sputum smear results ? (YES/NO)	Yes	Smear status affected diagnostic accuracy	No	Cost effectiveness analysis was only performed to smear negative specimens	Yes	Smear status in the initial test determined the follow up test	No		Yes	Smear status influenced the necessity for additional diagnosis and diagnostic accuracy	Yes	Smear status affected diagnostic accuracy
B	Is transmission of active pulmonary disease modeled? (YES/NO)	No		No		No		No	The active process of transmission was not modeled. However, the number of secondary cases	No	The active process of transmission was not modeled. However, the number of secondary cases were calculated	No	The active process of transmission was not modeled. However, the number of secondary cases were calculated

No.	Authors	Vassall *et al.*, 2011[17]		Chihota *et al.*, 2010[18]		Bonnet *et al.*, 2010[19]		Scherer *et al.*, 2009[20]		Dowdy *et al.*, 2008 (1)[21]		Dowdy *et al.*, 2008 (2)[22]	
	**Questions**	Is it modeled?	Details	Is it modeled?	Details	Is it modeled?	Details	Is it modeled?	Details	Is it modeled?	Details	Is it modeled?	Details
									were calculated from numbers of undiagnosed and untreated TB cases		from numbers of undiagnosed and untreated TB cases		from numbers of undiagnosed and untreated TB cases
C	Is the transmission probability stratified based on sputum smear result (negative smear has lower transmission probability)? (YES/NO)	No		No		No		No		No		Yes	Smear negative cases generated less secondary cases
D	<u>What is the mode of transmission?</u>												
i	Contact transmission												
ii	Household transmission												
iii	Other												
**5**	**MDR TB**												
A.	Is MDR TB incorporated in the model? (YES/NO)	Yes		No		No		No		No		No	
B	<u>How is MDR TB modeled?</u>												
i	Prevalence of MDR TB is considered	√											
ii	An exclusive state of MDR TB is modeled												
iii	Other												
C	<u>What are the impacts of MDR TB?</u>												
i	Higher treatment cost (second line)	√											
ii	Higher rate of failure	√	High rate of failure in patients correctly treated with second line treatment; and higher rate for those incorrectly treated with first line treatment										
iii	Lower transmission rate												
iv	Other												
D	Is diagnosis/screening strategy impact detection of MDR TB	Yes											
E	How does the diagnostic/screening strategy impact the detection of MDR TB		Xpert detected drug resistant faster, thus it allowed faster treatment with correct second line drug. However, all diagnosis strategy would be confirmed										

No.	Authors	Vassall *et al.*, 2011[17]		Chihota *et al.*, 2010[18]		Bonnet *et al.*, 2010[19]		Scherer *et al.*, 2009[20]		Dowdy *et al.*, 2008 (1)[21]		Dowdy *et al.*, 2008 (2)[22]	
	**Questions**	Is it modeled?	Details	Is it modeled?	Details	Is it modeled?	Details	Is it modeled?	Details	Is it modeled?	Details	Is it modeled?	Details
			by Drug Sensitivity Testing (DST)										
**6**	**Natural Recovery**												
A	Is natural recovery from TB active disease modeled? (YES/NO)	Yes	Only possible for HIV negative patients	No		No		No		No		No	
B	What are the consequences of TB natural recovery?												
i	Completely clear infection (return to susceptible)	√											
ii	Return to latent state												
iii	Combination of a and b												
iv	Other												
**7**	**Treatment**												
	Active Disease												
A	Is treatment of active disease modeled? (YES/NO)	Yes		No		No		Yes		Yes		Yes	
B	Which of the following treatment outcomes are incorporated to the model?												
i	Not receiving treatment	√	Undiagnosed patients could be re-diagnosed after 3 months. Before re-diagnosis, patients were subjected to natural recovery or death. A proportion of these patients also converted into smear positive upon re-diagnosis					√	Undiagnosed and untreated cases resulted in secondary cases	√	Undiagnosed and untreated cases resulted in secondary cases	√	Undiagnosed and untreated cases resulted in secondary cases
ii	Completion/treated successfully	√						√		√		√	
iii	Failure	√	Failure category included defaulters/loss to follow up										
iv	Lost to Follow Up (Non-compliance)												
C	What are the consequences of the treatment?												
C.1	Complete Treatment:												

No.	Authors / Questions	Vassall *et al.*, 2011[17] Is it modeled?	Details	Chihota *et al.*, 2010[18] Is it modeled?	Details	Bonnet *et al.*, 2010[19] Is it modeled?	Details	Scherer *et al.*, 2009[20] Is it modeled?	Details	Dowdy *et al.*, 2008 (1)[21] Is it modeled?	Details	Dowdy *et al.*, 2008 (2)[22] Is it modeled?	Details
i	Long-life Protection, out from the model	√	No re-infection or relapse cases were modeled					√	No re-infection or relapse cases were modeled	√	No re-infection or relapse cases were modeled	√	No re-infection or relapse cases were modeled until the end of the observation (1 year)
ii	Back to susceptible, rate of reinfection considered the same with primary infection												
iii	Back to susceptible, but receive partial immunity (lower rate of reinfection)												
iv	Return to latency												
v	Other												
C.2	<u>Failure</u>	Not detailed											
i	Return to latency												
ii	MDR TB												
iii	Category II												
iv	Other												
C.3	<u>Lost to Follow Up (Non-compliance)</u>												
i	Return to latency												
ii	MDR TB												
iii	Category II												
iv	Other												
**8**	**BCG Vaccination**												
A	Is BCG vaccinated population incorporated to the model? (YES/NO)	No		No		No		No		No		No	
B	<u>What is the impact of BCG vaccination towards infection probability?</u>												
i	Partial protection against acquiring disease												
ii	Reduced probability in disease progression												
iii	Combination of a and b												
iv	Other												
C	Is BCG vaccination status affecting diagnosis/screening result?												
D	How does BCG affect the diagnosis/screening result?												
**9**	**Comorbidity**												
A	Is comorbidity incorporated into the model? (e.g. HIV, Diabetes) (YES/NO)	Yes		No		No		Yes	HIV testing was performed and prevalence of TB in HIV infected patients was known.	Yes		Yes	
B	<u>How is the comorbidity incorporated into the model?</u>												
i	Select only the population with the specific comorbidity (e.g. patients with HIV)									√			
ii	Natural progression of comorbidity is modeled alongside TB and integration is depicted in several states												
iii	Comorbidity is presented as integrated states with TB												

No.	Authors	**Vassall *et al.*, 2011**[17]		**Chihota *et al.*, 2010**[18]		**Bonnet *et al.*, 2010**[19]		**Scherer *et al.*, 2009**[20]		**Dowdy *et al.*, 2008 (1)**[21]		**Dowdy *et al.*, 2008 (2)**[22]	
	**Questions**	Is it modeled?	Details	Is it modeled?	Details	Is it modeled?	Details	Is it modeled?	Details	Is it modeled?	Details	Is it modeled?	Details
iv	Comorbidity incidence/prevalence considered as input parameters in the model	√										√	
v	Other								Input parameters for the model were taken from population with known HIV status				
C	<u>What are the impact of comorbidity towards TB disease?</u>								Not detailed				
i	Alter rate of infection												
ii	Alter rate/probability of progression to active disease												
iii	Promote latency reactivation												
iv	Influence diagnosis accuracy (higher probability detected as smear negative)	√								√		√	
v	Alter probability of acquiring MDR TB												
vi	Other	√	HIV status affected treatment success rate and mortality rate						The patients' HIV status was known and recorded. However impact of HIV on TB progression was not detailed			√	HIV status affected mortality rate, smear status, and utility weight
**10**	**<u>Mortality</u>**												
A	Is mortality incorporated as one of the endpoints in the model? (YES/NO)	Yes		No		No		No				Yes	
B	<u>What type of mortality incorporated into the model?</u>	Not detailed											
i	TB-specific death									√		√	
ii	TB-specific death and other background mortality (as well as death due to comorbidity)												
**11**	**Extra pulmonary Tuberculosis**												
A	Is extra pulmonary TB incorporated into the model? (YES/NO)	No		No		No		No		No		No	

S4 Table 5. Data Extraction of Tuberculosis Progression Modeling Approaches (5)

No.	Authors	**Guerra *et al.*, 2008**[23]		**Mueller *et al.*, 2008**[24]		**Rajalahti *et al*,2004**[25]		**Dowdy *et al.*, 2003**[26]		**Roos *et al.*, 1998**[27]	
	**Questions**	Is it modeled?	Details	Is it modeled?	Details	Is it modeled?	Details	Is it modeled?	Details	Is it modeled?	Details
**1**	**Initial Prevalence**										
A	Is the initial prevalence data taken from the appropriate population?	Yes		Yes		Yes		Yes		Yes	
B	Is there any stratification of TB sates based on certain risk factor, such as age, social, and/or spatial heterogeneity? (YES/NO)	No		No		No		No		No	
**2**	**Progression from susceptible state following TB infection**										
A	Is acute and chronic progression to active disease modeled? (YES/NO)	No		No		No		No		No	
B	How is the acute and chronic progression modeled?										
i	A proportion of patients may progress directly to active disease (acute), while the other progress to latency/chronic state (no stratification of latency state)										
ii	All patients progress to early latent state, and from the state they can progress to active disease (acute) or late latency (chronic)										
iii	A proportion of patients may progress to fast progression state (acute), while other progresses to slow progression state (chronic)										
iv	Other										
**3**	**Latency/Chronic State Progression**										
A	Is accelerated progression from latency/chronic disease to active disease modeled? (YES/NO)	No		No		No		No		No	
B	What is the determinant of latency/chronic accelerated progression to active disease?										
i	Progression is due to exogenous reinfection										
ii	Progression is due to reactivation of the latent infection (e.g. in less immunocompetent patient)										
iii	Both i and ii										
iv	Other										
v	No clear determinant is explained										
**4**	**Active Disease**										
A	Are the patients with active disease categorized based on sputum smear results ? (YES/NO)	Yes	Smear status influenced diagnostic accuracy	No		Yes	Smear status influenced diagnostic accuracy	No	Study was performed exclusively in smear positive specimens	Yes	Smear status influenced the necessity for additional (follow up) diagnosis
B	Is transmission of active pulmonary disease modeled? (YES/NO)	No		No		No		No		No	
C	Is the transmission probability stratified based on sputum smear result (negative smear has lower transmission probability)? (YES/NO)	No		No		No		No		No	
D	<u>What is the mode of transmission?</u>										
i	Contact transmission										
ii	Household transmission										
iii	Other										
**5**	**MDR TB**										
A.	Is MDR TB incorporated in the model? (YES/NO)	No		No		No	MDR TB was considered in the study design. However no drug resistant cases were detected in the parent observation	No		No	

No.	Authors	Guerra *et al.*, 2008[23]		Mueller *et al.*, 2008[24]		Rajalahti *et al*,2004[25]		Dowdy *et al.*, 2003[26]		Roos *et al.*, 1998[27]	
	**Questions**	Is it modeled?	Details	Is it modeled?	Details	Is it modeled?	Details	Is it modeled?	Details	Is it modeled?	Details
							study, hence it was not added to the model				
B	How is MDR TB modeled?										
i	Prevalence of MDR TB is considered										
ii	An exclusive state of MDR TB is modeled										
iii	Other										
C	What are the impacts of MDR TB?										
i	Higher treatment cost (second line)										
ii	Higher rate of failure										
iii	Lower transmission rate										
iv	Other										
D	Is diagnosis/screening strategy impact detection of MDR TB										
E	How does the diagnostic/screening strategy impact the detection of MDR TB										
**6**	**Natural Recovery**										
A	Is natural recovery from TB active disease modeled? (YES/NO)	No		No		No		No		No	
B	What are the consequences of TB natural recovery?										
i	Completely clear infection (return to susceptible)										
ii	Return to latent state										
iii	Combination of a and b										
iv	Other										
**7**	**Treatment**										
	Active Disease										
A	Is treatment of active disease modeled? (YES/NO)	No		No		Yes	TB treatment and the related cost were included in the model	Yes	TB treatment and the related cost were included in the model	No	
B	Which of the following treatment outcomes are incorporated to the model?					Treatment outcome was not detailed		Treatment outcome was not observed			
i	Not receiving treatment										
ii	Completion/treated successfully										
iii	Failure										
iv	Lost to Follow Up (Non-compliance)										
C	What are the consequences of the treatment?										
C.1	Complete Treatment:										
i	Long-life Protection, out from the model										
ii	Back to susceptible, rate of reinfection considered the same with primary infection										
iii	Progress to recovered state with partial immunity against reinfection										

No.		Authors	**Guerra *et al.*, 2008**[23]		**Mueller *et al.*, 2008**[24]		**Rajalahti *et al*,2004**[25]		**Dowdy *et al.*, 2003**[26]		**Roos *et al.*, 1998**[27]	
		**Questions**	Is it modeled?	Details	Is it modeled?	Details	Is it modeled?	Details	Is it modeled?	Details	Is it modeled?	Details
	iv	Return to latency										
	v	Other										
	C.2	<u>Failure</u>										
	i	Return to latency										
	ii	MDR TB										
	iii	Category II										
	iv	Other										
	C.3	<u>Lost to Follow Up (Non-compliance)</u>										
	i	Return to latency										
	ii	MDR TB										
	iii	Category II										
	iv	Other										
**8**		**BCG Vaccination**										
	A	Is BCG vaccinated population incorporated to the model? (YES/NO)	No		No		No		No		No	
	B	<u>What is the impact of BCG vaccination towards infection probability?</u>										
	i	Partial protection against acquiring disease										
	ii	Reduced probability in disease progression										
	iii	Combination of a and b										
	iv	Other										
	C	Is BCG vaccination status affecting diagnosis/screening result?										
	D	How does BCG affect the diagnosis/screening result?										
**9**		**Comorbidity**										
	A	Is comorbidity incorporated into the model? (e.g. HIV, Diabetes) (YES/NO)	No		No		No		No		No	
	B	<u>How is the comorbidity incorporated into the model?</u>										
	i	Select only the population with the specific comorbidity (e.g. patients with HIV)										
	ii	Natural progression of comorbidity is modeled alongside TB and integration is depicted in several states										
	iii	Comorbidity is presented as integrated states with TB										
	iv	Comorbidity incidence/prevalence considered as input parameters in the model										
	v	Other										
	C	<u>What are the impact of comorbidity towards TB disease?</u>										
	i	Alter rate of infection										
	ii	Alter rate/probability of progression to active disease										
	iii	Promote latency reactivation										
	iv	Influence diagnosis accuracy (higher probability detected as smear negative)										
	v	Alter probability of acquiring MDR TB										
	vi	Other										
**10**		**<u>Mortality</u>**										
	A	Is mortality incorporated as one of the endpoints in the model? (YES/NO)	No		No		No		No		No	
	B	<u>What type of mortality incorporated into the model?</u>										

No.	Authors	**Guerra *et al.*, 2008**[23]		**Mueller *et al.*, 2008**[24]		**Rajalahti *et al*,2004**[25]		**Dowdy *et al.*, 2003**[26]		**Roos *et al.*, 1998**[27]	
	**Questions**	Is it modeled?	Details	Is it modeled?	Details	Is it modeled?	Details	Is it modeled?	Details	Is it modeled?	Details
i	TB-specific death										
ii	TB-specific death and other background mortality (as well as death due to comorbidity)										
**11**	**Extra pulmonary Tuberculosis**										
A	Is extra pulmonary TB incorporated into the model? (YES/NO)	No		No		No		No		No	

Please see S1 Table for list of references
